# Next-Generation Lipid Prodrugs Orally Deliver Tenofovir via Enhanced Chylomicron Incorporation

**DOI:** 10.1021/acsptsci.5c00237

**Published:** 2025-08-22

**Authors:** Hannah B. Gold, Nicole Pribut, Esther L. Outtrim, Priscilla Davidson, Christopher M. Monaco, August Myers, Carrie Qi Sun, Goknil Pelin Coskun, Andrea Mancia, Yanli Yang, Samantha Burton, Areeb Aftab, Cynthia A. Derdeyn, Rebecca S. Arnold, John A. Petros, Ken Liu, Eric J. Miller, Dennis C. Liotta

**Affiliations:** † Department of Chemistry, 1371 Emory University College of Arts & Sciences, Atlanta, Georgia 30322, United States; ‡ Department of Urology, 12239 Emory University School of Medicine, Atlanta, Georgia 30322, United States; § Department of Laboratory Medicine & Pathology, 7284 University of Washington School of Medicine, Seattle, Washington 98195, United States; ∥ Washington National Primate Research Center, Infectious Diseases & Translational Medical Unit, Seattle, Washington 98195, United States; ⊥ Department of Pharmacology & Chemical Biology, Emory University School of Medicine, Atlanta, Georgia 30322, United States

**Keywords:** lipids, lymphatic system, lipoproteins, prodrugs, absorption, distribution, chylomicrons, tenofovir, HIV

## Abstract

Lipid-derived prodrugs can enhance the oral bioavailability of therapeutic agents by promoting uptake into the lymphatic system. Chylomicrons (CMs) are lipoproteins that transport dietary lipids into the lymphatics. However, their role in the systemic distribution of orally administered lipid prodrugs remains understudied. We developed an *in vitro* assay demonstrating that human intestinal enterocyte-like cell-derived CMs incorporate lipid prodrugs, and that the efficiency of this incorporation varies as a function of the lipid promoiety. We synthesized a series of lipid prodrugs of tenofovir (TFV), an important but poorly bioavailable anti-HIV agent. These lipid prodrugs, featuring a benzyloxyglycerol (BOG) motif and/or an ω-CF_3_ group, demonstrated improved metabolic stability and favorable antiviral activity *in vitro*, relative to unfunctionalized lipid conjugate TFV exalidex (TXL). Additionally, the ω-CF_3_ and BOG modifications significantly increased prodrug uptake into CMs *in vitro*. Subsequent mouse pharmacokinetic (PK) studies revealed higher systemic drug levels of orally dosed ω-CF_3_ BOG prodrugs relative to TXL, as well as substantially enhanced lung distribution. This study is the first to quantify drug incorporation into human intestinal enterocyte-like cell-derived CMs using LC-MS/MS. In conclusion, highly lipophilic TFV prodrugs efficiently incorporate into CMs *in vitro*, and mouse PK data is consistent with lymphatic absorption *in vivo*, providing a framework for the rational design and screening of lipid-based prodrugs for optimization of drug distribution.

## Graphical abstract

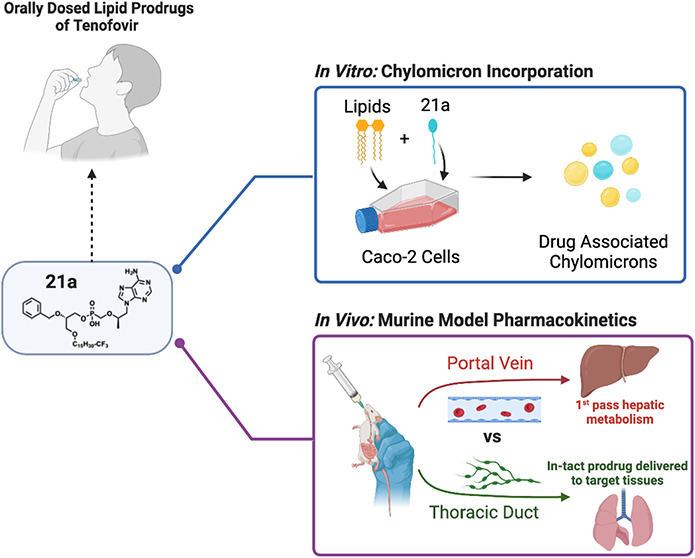

All orally ingested fats, whether they are consumed through food, supplements, or medication, require absorption from the gastrointestinal (GI) lumen into intestinal enterocytes, a monolayer of polarized epithelial cells that sort incoming nutrients, before distribution to target tissues.^1–6^ While dietary fats, such as triglycerides (TG), phospholipids (PL), and cholesterol (CHOL) esters, are not readily taken up by enterocytes, catabolic metabolism in the GI tract facilitates conversion to the corresponding fatty acids, monoacylglycerols, and CHOL, which are much more cell-permeable. Upon absorption into enterocytes, these lipids, which have limited solubility in the cytosolic compartment to begin with, are anabolized back to TG, PL, and CHOL esters, which aggregate together in endoplasmic reticula (ER) and Golgi. These insoluble lipid aggregates are then packaged into chylomicrons (CMs, Figure 1), which are large lipoproteins that facilitate physiological lipid transport through hydrophilic environments^7,8^ (*e.g*., plasma and interstitial fluid). Upon secretion across basolateral enterocyte membranes into lamina propria, CMs are taken up by intestinal lymphatic capillaries. While most dietary nutrients and orally administered medications are hepatically absorbed into circulation through intestinal blood capillaries to mesenteric venous capillaries, which drain into the portal vein and funnel directly to the liver, CMs are too large (75–1200 nm) to penetrate through the tight intercellular junctions of hepatic venous capillaries.^9^ Instead, they are lymphatically absorbed into mesenteric lymph capillaries with fenestrations large enough to accommodate CMs. These lymphatic vessels converge into the thoracic duct, which releases its contents into systemic circulation at the left venous angle, just before the blood returns to the heart and subsequently enters pulmonary circulation. As a consequence, orally ingested xenobiotics capable of associating with CMs and following this lymphatic absorption pathway may avoid direct transport to and first-pass metabolism by the liver.

**Figure 1:**
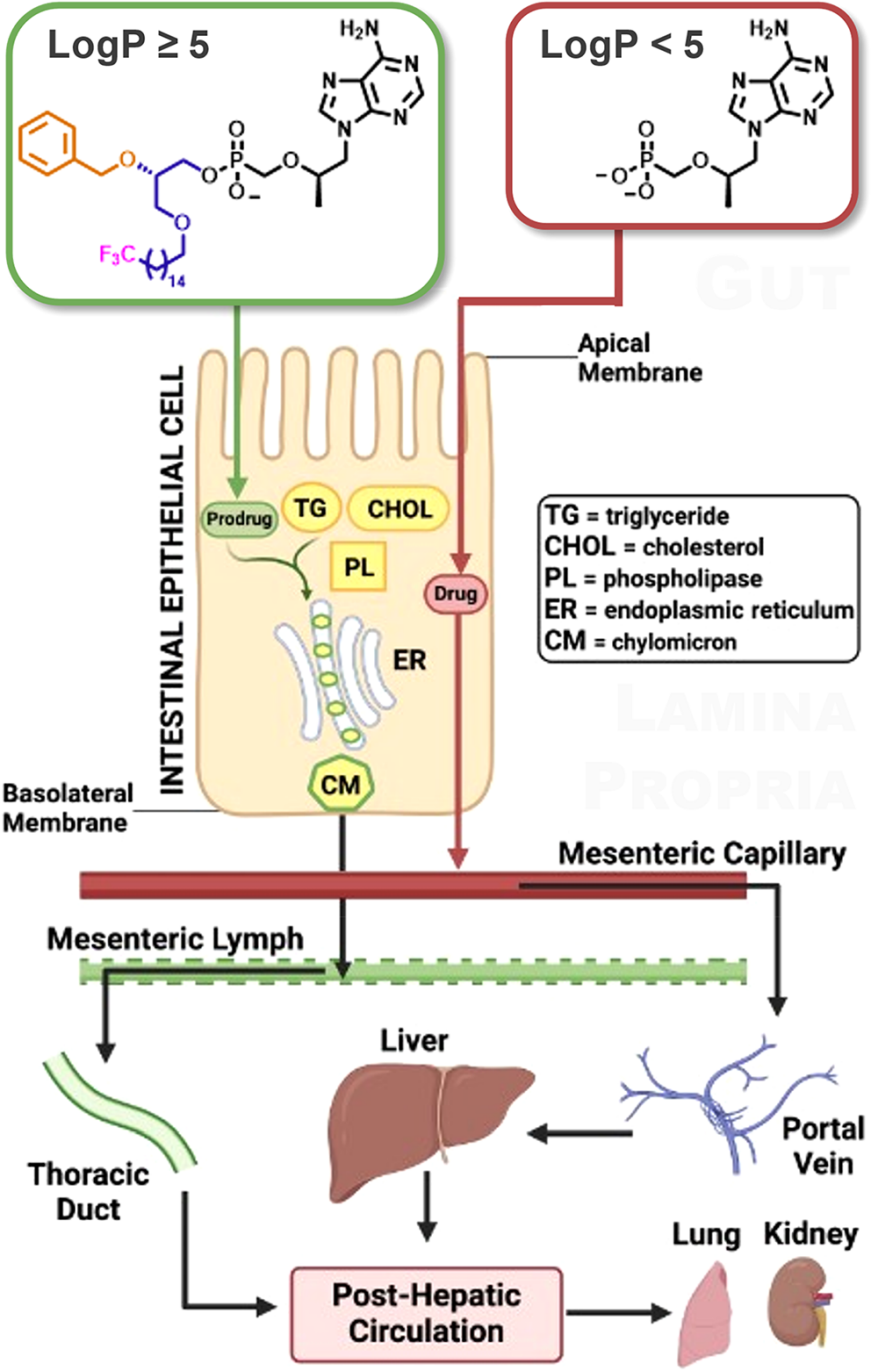
Preferential packaging of lipophilic drugs into CMs for lymphatic delivery. Lipophilic drugs with Log *P* ≥ 5 can be packaged into CMs within intestinal epithelial cells and taken up by the lymphatic system, bypassing the portal venous system and circumventing first-pass metabolism in the liver. Graphic created using Biorender.

Several strategies are in development to deliver poorly orally bioavailable drugs via the lymphatic system.^6^ One approach involves conjugating chemotherapeutic, antiviral, or immunomodulatory agents to dietary lipid motifs, the hypothesis being that these prodrugs will follow the same absorption pathway as a lipid.^10–13^ Several existing lipid prodrugs and their properties are described in Table 1. The Porter group has made significant contributions to the field through the development of triglyceride-mimetic prodrugs of poorly bioavailable medications including mycophenolic acid (MPA),^14^ testosterone,^15^ and buprenorphine (BUP).^16^ These prodrugs were shown to enhance oral bioavailability, resulting in increased plasma exposure compared to the parent drug. Importantly, several groups have demonstrated, using lymph duct cannulation and mesenteric lymph node dissection, that drug-lipid conjugates achieve significantly higher concentrations in the lymphatics than the unmodified drug. For example, testosterone undecanoate (TU), an ester prodrug of the poorly bioavailable testosterone hormone, was shown to concentrate in the lymphatics following oral administration, with lymphatic transport accounting for 91.5–99.7% of its systemic availability.^17^ Complementarily, the Hostetler group found that 1-*O*-octadecyl-2-*O*-benzyl-*sn*-glycero-3-cidofovir (**ODBG-CDV**), a glycerolipid conjugate of antiviral agent cidofovir (**CDV**), preferentially distributed into lung over liver after oral dose. Together, these results support the hypothesis that drug association with CMs in intestinal enterocytes can (at least partially) circumvent the portal venous system via the lymphatic absorption pathway.^18^

**Table 1:**
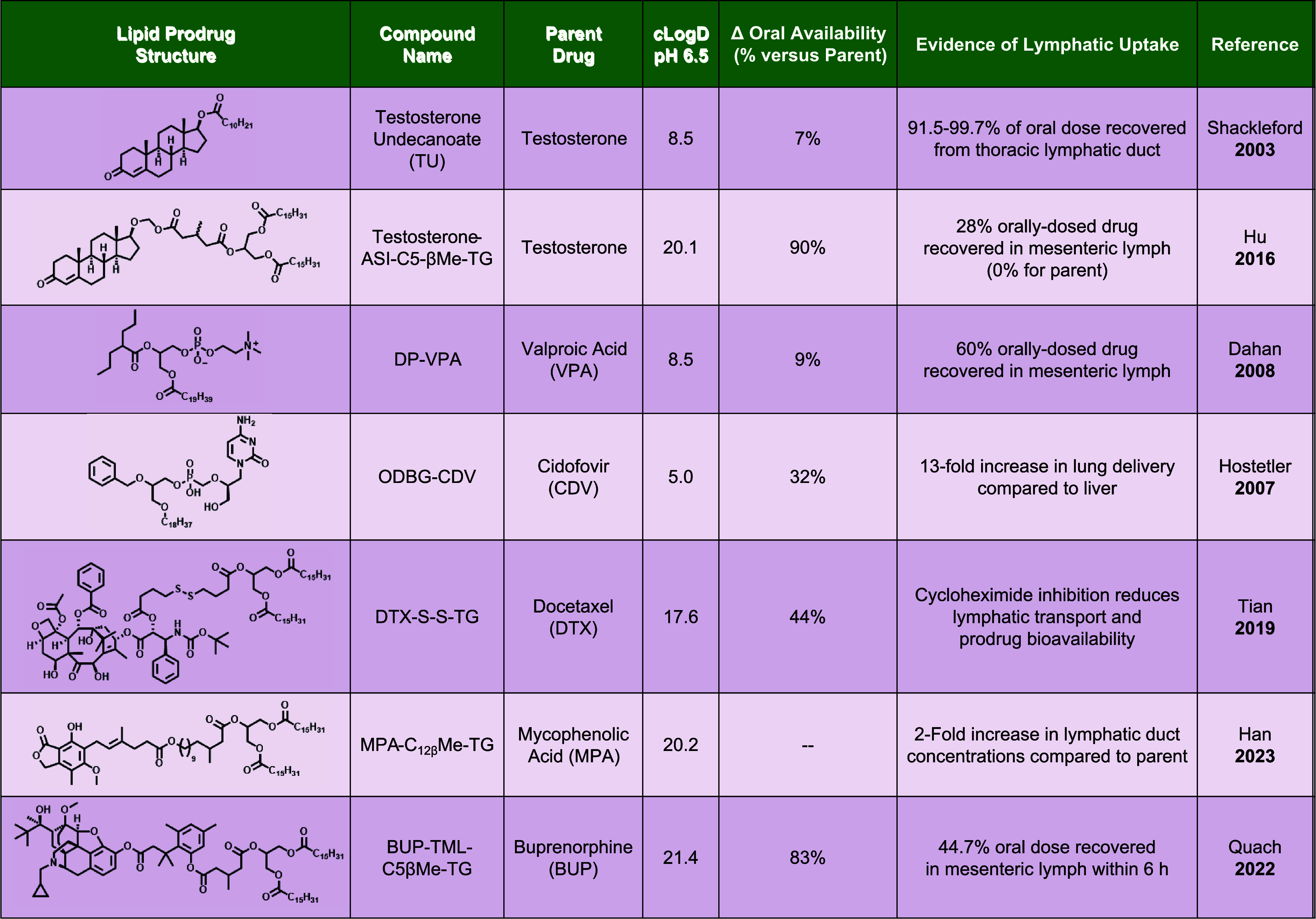
Literature Examples of Lipid Prodrugs and Evidence of Lymphatic Uptake^a^

a Summary of reported lipid prodrugs, *c* Log *D* values at intra-intestinal pH 6.5, relative oral bioavailability, and evidence of enhanced lymphatic uptake compared to the parent drug. DP = D Pharma. ODBG-CDV = 1-*O*-octadecyl-2-*O*-benzyl-*sn*-glycero-3-cidofovir. TG = triglyceride. MPA = mycophenolic acid. TML = trimethyl lock.

Because the lymphatic absorption of orally ingested xenobiotics depends on CM incorporation, we were interested in measuring the partitioning of highly lipophilic prodrugs in intestinal enterocytes *in vitro* and characterizing oral absorption and distribution *in vivo*. Reviewing the literature on CM biogenesis and the role of CMs in physiological lipid trafficking revealed that much of what is known about the role of CMs in lipid transport, processing, and related diseases stems from studies involving isolation and characterization of endogenous CMs from human serum.^19–22^ Synthetic CMs are also being employed as nanocarriers for drug delivery. For example, CM mimics called emulsomes have been shown to efficiently encapsulate poorly soluble drugs and improve bioavailability.^23^ Other *in vitro* approaches involve the use of artificial CMs to quantify drug incorporation as a predictor of lymphatic uptake.^24^ Alternatively, the Nauli group demonstrated that Caco-2 cells, a readily available human intestinal enterocyte-like cell line, can independently produce CMs and very low-density lipoproteins (VLDLs) *in vitro*.^25^ This model more closely mimics the *in vivo* production of CMs within the human intestinal epithelium. To build on this assay protocol, we were interested to explore the incorporation of highly lipophilic lipid prodrugs into Caco-2 cell-derived CMs during CM biogenesis. We envisioned that CM production could be stimulated using Nauli’s protocol, but with prodrug-spiked media instead of prodrug-free media, followed by isolation and characterization of prodrug-loaded CMs using high-speed ultracentrifugation and liquid chromatography-tandem mass spectrometry (LC-MS/MS), respectively.^26,27^

As a formidable testing ground, we leveraged a series of lipid prodrugs of tenofovir (**TFV**), an HIV nucleotide reverse transcriptase inhibitor (NtRTI) that is widely recognized for its efficacy in the treatment of HIV and chronic HBV infections (Figure 2a).^28^ At physiologic pH, **TFV** exists as a dianion, which significantly limits cell permeability and oral bioavailability.^28^ **TFV** is thus administered exclusively in prodrug form.^29^ The two FDA-approved **TFV** prodrugs are TFV disoproxil fumarate (**TDF**, Figure 2b) and TFV alafenamide (**TAF**, Figure 2c). By masking the negatively charged phosphonate of **TFV**, **TDF**, and **TAF** each improve cell permeability and enable oral delivery of **TFV**. However, **TDF** is cleaved readily by circulating plasma esterases, and both **TDF** and **TAF** undergo significant metabolism in the liver. This directly compromises the benefits of the prodrug moiety, as the release of **TFV** into liver substantially limits drug availability to extra-hepatic tissues, while **TFV** release in plasma causes accumulation in the kidney, resulting in nephrotoxicity^30^ and bone mineral density depletion, among other severe adverse side effects.^28,31,32^ As such, there is a need for next-generation **TFV** prodrugs with improved metabolic stability in circulation that can be much more efficiently delivered to HIV-infected cells. One avenue under exploration is the conjugation of nucleotide-based therapeutics to lysoglycerophospholipids (Figure 3), as exemplified by hexadecyloxypropyl (HDP)-TFV (Figure 3b), also known as CMX-157 or TFV exalidex (**TXL**), and HDP-CDV (Figure 3a), also known as CMX-001, brincidofovir (**BCV**), or Tembexa. Both **TXL** and **BCV** have demonstrated improved oral bioavailability and antiviral activity *in vivo*, relative to their unconjugated parent drugs, *i.e*., **TFV** and **CDV**.^33,34^ Mechanisms of action for these lipid prodrugs depend on their ability to undergo intracellular cleavage by phospholipase C,^35^ sphingomyelinase,^36,37^ or other membrane-associated lipid hydrolase. Enzymatic activation at the inner leaflet of the plasma membrane releases the parent nucleotide, **TFV** or **CDV**, inside target cells, thus avoiding premature cleavage in plasma.^35^

**Figure 2:**
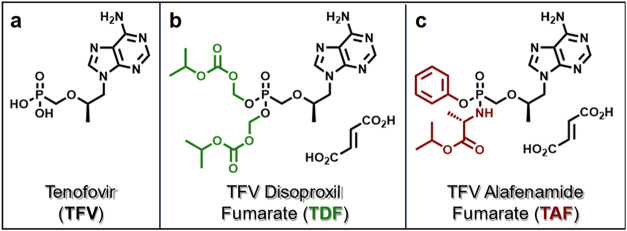
Structures of (a) antiretroviral agent tenofovir (**TFV**) and its FDA-approved prodrugs (b) **TFV** disoproxil fumarate (**TDF**) and (c) **TFV** alafenamide fumarate (**TAF**).

**Figure 3:**
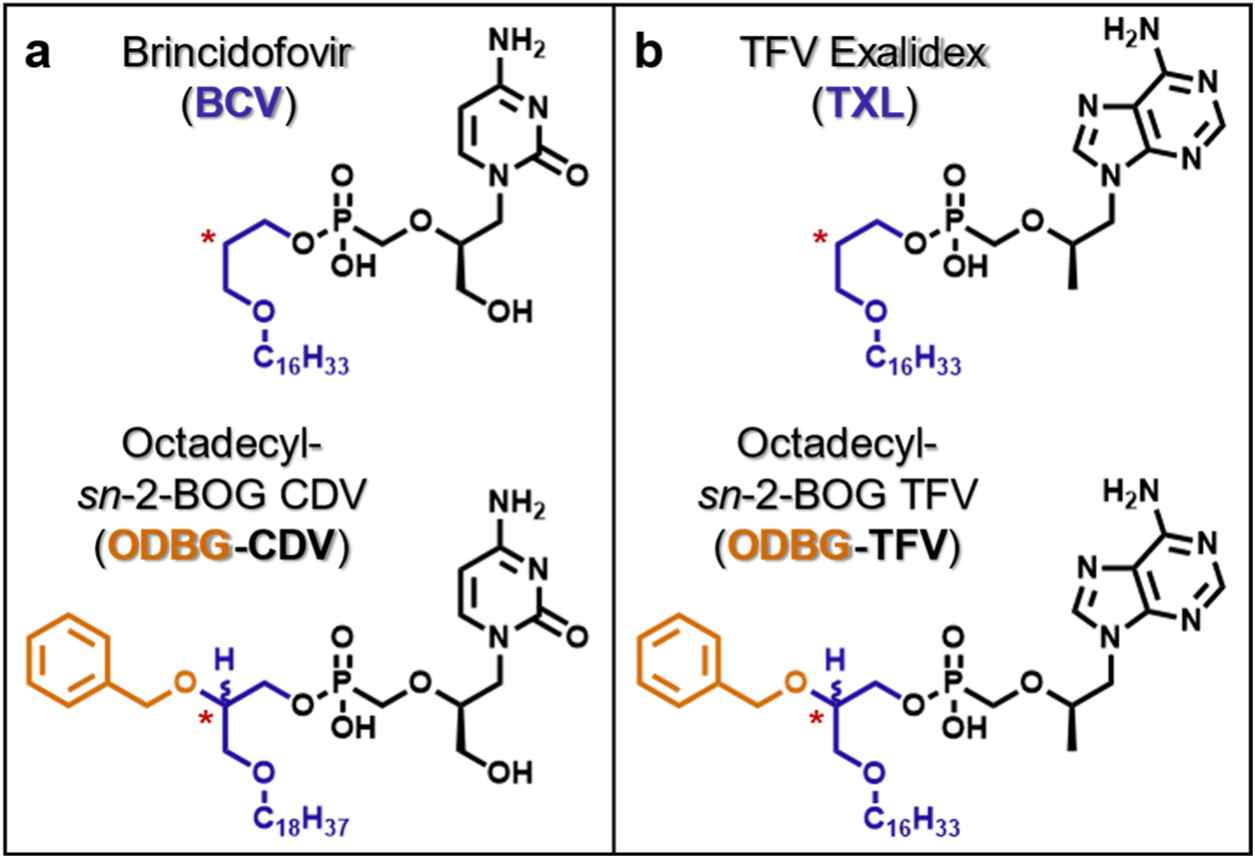
Structures of lysoglycerophospholipid-derived antiviral prodrugs with and without *sn*-2 benzyloxyglycerol (BOG) modifications. Comparing (a) **CDV** and (b) **TFV** prodrugs with and without the BOG moiety. *Indicates the *sn*-2 glycerol position.

As mentioned above, Hostetler and colleagues advanced the concept of lysoglycerophospholipid conjugation to nucleotide-based therapeutics by characterizing the tissue distribution profiles of **BCV** relative to its more lipophilic congener **ODBG-CDV** (Table 1 and Figure 3a).^18^ This approach introduces a benzyloxyglycerol (BOG) linker featuring a benzyl appendage on the *sn*-2 glycerol hydroxyl group. After a single oral dose, **ODBG-CDV** and **BCV** showed distinct tissue distribution patterns, as characterized by measuring prodrug concentrations in lung and liver over 72 h. **ODBG-CDV** (lung/liver AUC_0–72h_ = 2.05) demonstrated substantially enhanced extra-hepatic exposure compared to its unfunctionalized counterpart **BCV** (lung/liver AUC_0–72h_ = 0.04). This dynamically shifted and preferential distribution to the lung is proposed to result from increased lymphatic uptake of **ODBG-CDV** relative to **BCV**.^18^ This strategy could be particularly important for treating respiratory diseases like COVID-19, where effective antiviral concentrations in the lung are essential for controlling viral replication. In fact, this approach has been applied to GS-441524, the parent nucleoside of remdesivir, leading to increased oral bioavailability, enhanced extra-hepatic tissue distribution, and improved SARS-CoV-2 antiviral efficacy in preclinical models.^38,39^ These literature reports highlight BOG-functionalization of lysophospholipid prodrugs as a useful tool to improve antiviral drug delivery through intestinal lymphatic absorption.

However, given that **ODBG-CDV** features 2 extra carbons in the lipid chain relative to **BCV**, in addition to the BOG group, stereochemistry for which is not defined, the exact structural determinants of **ODBG-CDV**’s unique tissue distribution profile remain unclear. Furthermore, previous research from our interdisciplinary team demonstrated significant susceptibility of the lipid tail ω-position to cytochrome P450 (CYP)-mediated oxidative metabolism in the liver, as well as an ω-functionalization strategy to mitigate this undesired and premature lipid metabolism.^40^ Replacing the ω-CH_3_ group with, for example, an ω-CF_3_ group dramatically improved metabolic stability by thermodynamically and electronically disfavoring ω-oxidation, which otherwise initiates the typical endogenous catabolic lipid β-oxidation cascade.^40,41^ It is important to highlight here that all orally ingested xenobiotics undergo partitioning within intestinal enterocytes between cytosolic compartments and lipoprotein-generating compartments. Accordingly, while a very large fraction of a highly hydrophilic drug (*e.g*., metformin, **MET**) follows the hepatic absorption pathway, a very small fraction undergoes lymphatic uptake. Similarly, even though a large fraction of a highly lipophilic prodrug (*e.g*., **ODBG-TFV**) designed to target lymphatic absorption will incorporate into CM, some fraction will inevitably reach the liver during first-pass, as will all intact prodrug molecules during second-pass and third-pass and so on. Therefore, mitigating premature hepatic metabolism remains an important consideration, even for lipid prodrugs.

Accordingly, we synthesized a preliminary series of ω-unsubstituted, BOG-functionalized lipid prodrugs of **TFV**, as well as a second-generation series of ω-CF_3_, BOG-functionalized lipid prodrugs of **TFV**. In principle, the ω-CF_3_, group should, not only confer metabolic stability by rendering the prodrugs resistant to ω-oxidation,^42^ but will also itself effect lipophilicity. One study in particular showed that substituting a C–H bond for a C–F bond increased Log *P* values from 0.6–1.4 units.^43^ Thus, to enable accurate comparison and isolate the specific influence of the BOG motif, the lipid tail, and the lipid terminus, we also synthesized ω-CF_3_-functionalized and unfunctionalized HDP and octadecyloxypropyl (ODP) lipid prodrugs lacking the BOG modification. All synthesized lipid prodrugs, both with and without BOG and lipid terminal modifications, were evaluated for their ability to associate with Caco-2 cell-derived CMs *in vitro*. This assay, the details of which are reported herein, allowed us to characterize, and therefore to differentiate, lipid prodrugs base on their partitioning preferences in intestinal enterocytes. Following initial *in vitro* profiling and quantifying lipid prodrug packaging into CMs, we designed a pharmacokinetics (PK) experiment to determine whether observations made using our *in vitro* CM assay would be predictive of lymphatic delivery *in vivo*. To this end, we administered a single oral dose of **TFV** lipid prodrug to mice and analyzed their tissue-specific distribution over 18–24 h. We were particularly interested in liver, which is primarily responsible for drug metabolism, and lung, which is the first postcardiac organ exposed to lymphatically distributed prodrug after oral dose. These data highlight the superior stability and tissue-targeting potential of ω-CF_3_ BOG prodrugs, particularly in overcoming rapid hepatic clearance and enhancing delivery to extra-hepatic target tissues. Taken together, our findings provide insight into the dynamic process of intracellular drug packaging into lipoproteins that undergo lymphatic uptake. As highlighted by the significant impact of even subtle lipid modifications on drug distribution patterns, this opens avenues for novel prodrug design and optimization for systemic drug delivery.

## Results and Discussion

### Validation of CM Isolation Using TEM and ApoB ELISA

The methodology used in this study for the production and isolation of CMs (Scheme 1) was adapted from an *in vitro* model developed by the Nauli group.^25^ They reported that fully differentiated (13–17 days postconfluence) human intestinal enterocyte-like (Caco-2) cells can produce CMs of physiologic size when stimulated with a lipid mixture. According to the reported methods, Caco-2 cells were cultured to confluence and then differentiated under 14-day incubation. After this time, differentiated Caco-2 cells were stimulated to produce CMs via incubation in a precisely proportioned lipid mixture (2:1.36:1 OA/lecithin/NaTC) comprised of oleic acid (OA), lecithin, and sodium taurocholate (NaTC), as optimized by the Nauli group for maximal CM production. After 20 h of incubation, the culture media was collected, and CMs were isolated by adding a 1.182 g/mL NaCl solution, followed by density-gradient ultracentrifugation at 10,950 RCF, according to Nauli’s protocol.^25^ Due to their low density (<0.94 g/mL),^44^ CMs migrated to the top layer of the gradient, as indicated by staining with Red Oil O (Sigma #O0625), and the supernatant was carefully collected using a fine-tip pipet.

**Scheme 1:**
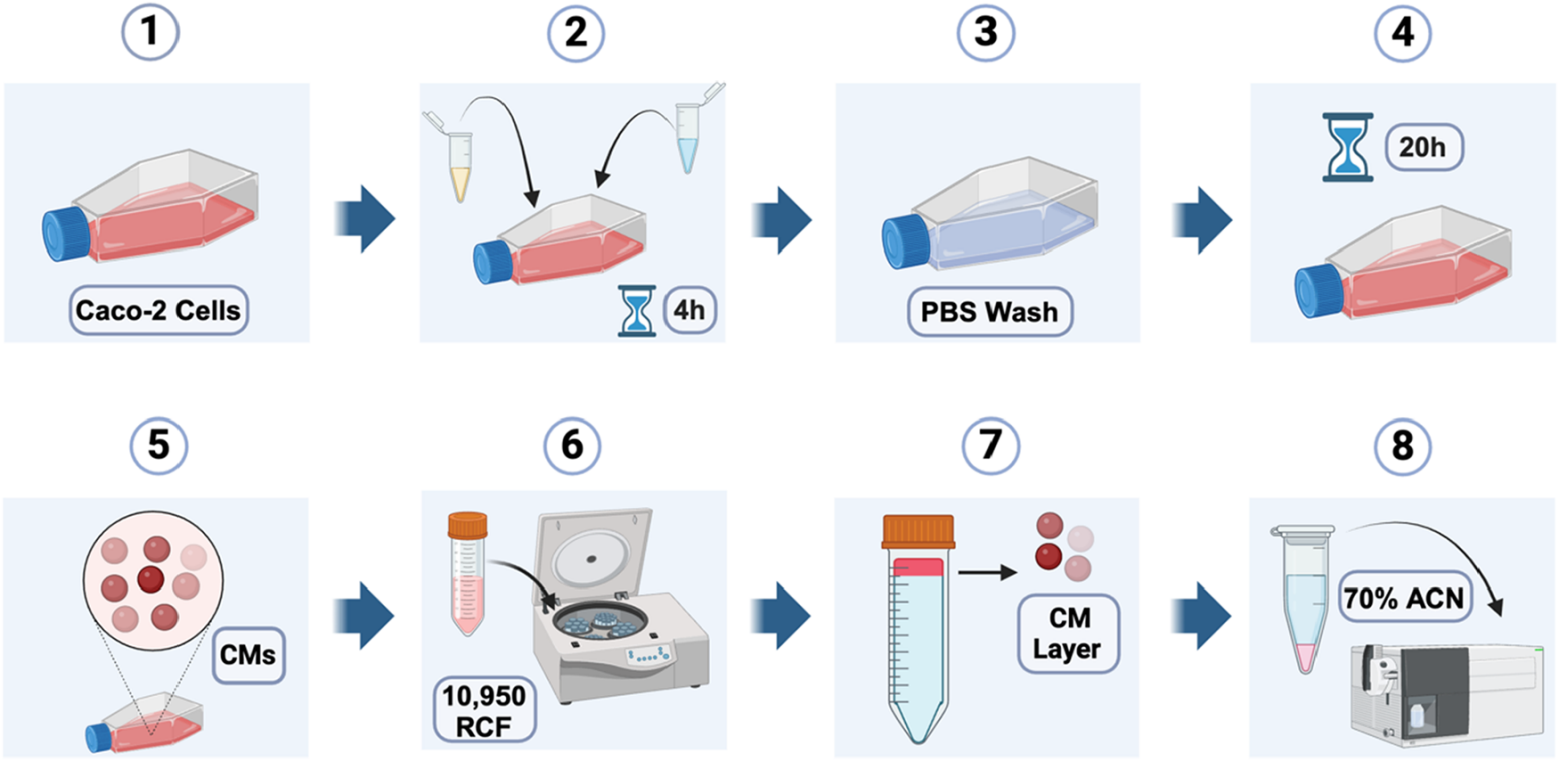
Chylomicron (CM) Isolation and Drug Quantification Methods^a^

a (1) Caco-2 cells were grown to confluence. (2) Lipid mix was added to media (final drug concentration of 10 μM), and cells were incubated for 4 h. (3) Cells were washed 3× with PBS. (4) Cells were incubated in fresh media overnight. (5) CM-containing media was collected. (6) Media was combined with a 1.20 g/mL NaCl solution in an ultracentrifugation tube, 500 μL H_2_O was carefully layered on top, and the mixture was centrifuged at 10,950 RCF for 3 h. (7) The CM-containing upper layer was carefully isolated with a fine-tip pipette. (8) Test drug was then extracted from the CM layer with 70% ACN and quantified using LC-MS/MS.

The presence of CMs was confirmed using two orthogonal experimental techniques, the results of which are shown in Figure 4. First, transmission electron microscopy (TEM) with negative staining revealed uniform spherical particles ranging from 250 to 500 nm in size (Figure 4a), consistent with the expected size range of CMs.^9^ To confirm these findings, we used a commercially available ELISA kit targeting ApoB-48, a protein unique to the CM surface and a definitive marker for these lipoproteins.^45^ Results from this assay provided quantitative data on the concentration of ApoB-48, serving as a surrogate marker for CMs in the sample. Concentrations in test samples (Figure 4c) were calculated based on a standard calibration curve (Figure 4b) that correlated ApoB concentration with absorbance.

**Figure 4:**
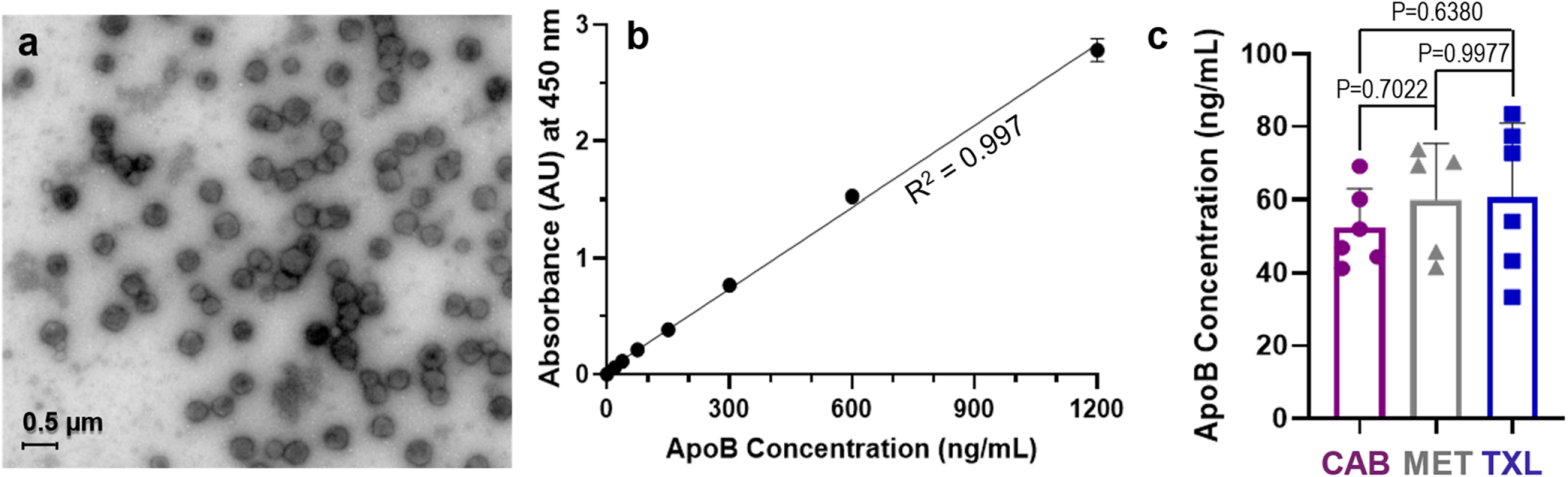
Detecting CMs using TEM and ApoB ELISA. (a) Transmission electron microscopy (TEM) images show spherical particles ranging from 250 to 500 nm, characteristic of CMs. (b) Standard curve absorbance values were obtained from Apolipoprotein B (ApoB) ELISA standards (*n* = 2) and plotted with the concentration of ApoB on the *x*-axis linearly correlated with absorbance at 450 nm on the *y*-axis. (c) Concentration of ApoB-48 in isolated CM samples was consistent across three test compounds was quantified (*n* = 6 per compound). Statistical comparisons between groups using 1-way ANOVA (*P* > 0.05) indicated insignificant differences in ApoB levels (as a surrogate for CM levels) between different compounds. CAB = cabazitaxel. MET = metformin. TXL = tenofovir exalidex.

Results show a consistent ApoB-48 concentration of 60–70 ng/mL across replicates from different CM experiments, including in the presence of representative low (**MET**, Figure 5b) and high (**TXL**, Figure 5c) lipophilicity compounds. While it is worth noting that enterocytes can produce low amounts of small ApoB-expressing VLDLs that are not necessarily size-excluded from intestinal and hepatic blood capillaries, the large majority of ApoB-48 is expressed by definitively size-excluded CM and VLDL. These results confirmed the reproducibility of the assay, thus enabling direct comparison between studies across different drugs.

**Figure 5:**
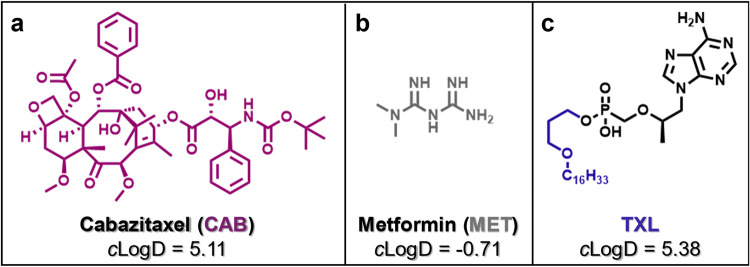
Screening compounds used to validate the CM assay. Structures of (A) cabazitaxel (**CAB**, positive control), (B) metformin (**MET**, negative control), and (C) **TXL** with their respective *c* Log *D* values at intestinal pH 6.5. *c* Log *D* calculated using Schrodinger Maestro (LigPrep and QikProp).

### CMs as Carriers for Lipophilic Drugs

With the presence of CMs in isolated Caco-2 cell extracts confirmed and reproducibility validated across replicates and different drugs, we were eager to investigate drug incorporation during CM biogenesis *in vitro*. Accordingly, we added test compound (Figure 5) to differentiated Caco-2 cells along with the lipid mix to a final test drug concentration of 10 μM. While 10 μM was selected as a standard concentration for these studies, higher concentrations could allow detection of other compounds with low CM association in future experiments. In either case, CMs secreted by Caco-2 cells can be isolated postincubation, and drug concentrations within CMs can be assessed using highly sensitive LC-MS/MS methodology. Positive (high *c* Log *D*) and negative (low *c* Log *D*) control compounds were selected to validate this assay. Cabazitaxel (**CAB**, Figure 5a), a taxol derivative used in the treatment of prostate cancer, was chosen as a positive control due to its high lipophilicity at intestinal pH 6.5 (*c* Log *D* = 5.11), as well as its reported accumulation in mesenteric lymph^46^ and liposomes.^47^ Given these properties, we expected **CAB** to readily package into CMs *in vitro*. In contrast, **MET**, a widely used biguanide for type 2 diabetes mellitus treatment, was selected as a negative control due to its high hydrophilicity at pH 6.5 (*c *Log *D* = −0.71) rendering it unlikely to associate with lipid-rich CMs. LC-MS/MS confirmed the presence of **CAB** (*n* = 6 biological replicates) and the absence of **MET** (*n* = 9 biological replicates) in CM extract under these experimental conditions (Figure 6), and quantitation of the respective concentrations in CMs established a working assay window for this approach. Following assay validation with these positive and negative controls, we screened **TXL**, the key reference standard, which was successfully detected in CM isolates. This sets the stage for quantitative comparisons of **TXL** and its functionalized analogs, which are detailed below.

**Figure 6:**
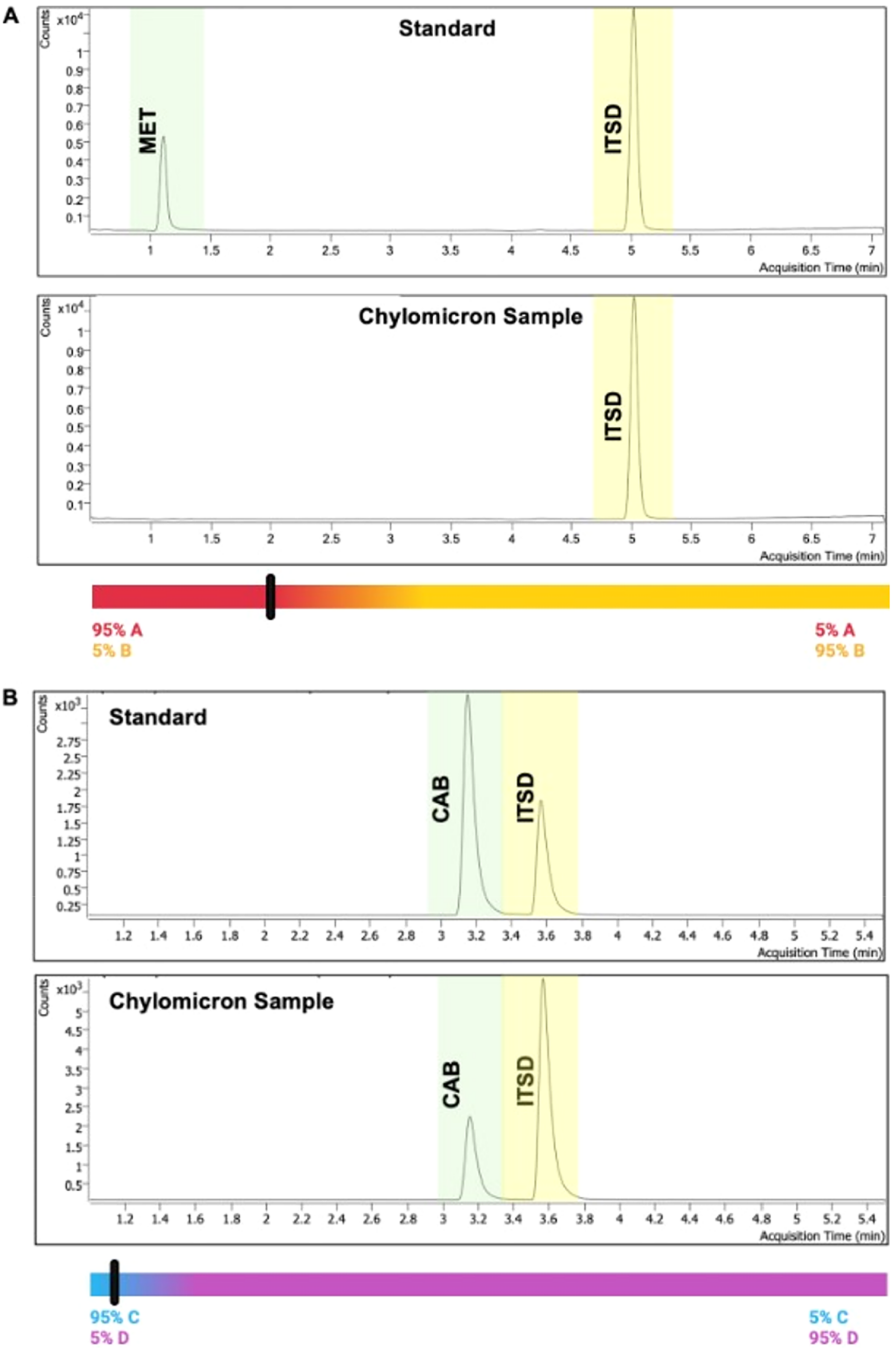
Reverse phase (C18) LC-MS/MS chromatograms of standard test compounds and CM extracts after incubation with these test compounds. (A) Chromatogram (using the indicated gradient of mobile phase A and mobile phase B) of metformin (**MET**) and (B) chromatogram (using the indicated gradient of mobile phase C and mobile phase D) of cabazitaxel (**CAB**). Mobile phase A: 2 mM ammonium acetate (AA) in H_2_O; Mobile phase B: 100% ACN; Mobile phase C: 40% ACN + 1 mM AA in H_2_O. Mobile phase D: 10% ACN + 1 mM AA in *i*PrOH. ITSD = internal standard, *d*_5_-ethoxycoumarin.

### ω-Modified and Unmodified BOG Lipid Prodrug Synthesis

Once we confirmed standard drug association with CMs, we set out to screen a new series of compounds to compare their propensity for CM association *in vitro* as a potential predictor of intestinal lymphatic uptake following oral dose *in vivo*. The Hostetler group demonstrated that a lysoglycerophospholipid prodrug **ODBG-CDV** (Figure 3a) exhibits enhanced lung distribution compared to the less lipophilic **BCV**, presumably due to improved CM-mediated lymphatic uptake. Given that the lung is both an early target for HIV and a known anatomical reservoir for the virus, there is a need for **TFV** prodrugs with similar properties.^48^ **CDV** and **TFV** share similarities as acyclic nucleotide phosphonates that act as viral DNA chain terminators, making them highly effective antiviral agents. However, both compounds exist as dianions under physiological pH, necessitating covalent functionalization (or other nanoformulation strategy) to improve their bioavailability.

Building upon Hostetler’s findings, we synthesized analogous phospholipid prodrugs of **TFV** to investigate how structural modifications to the lipid promoiety influence CM packaging and lymphatic absorption. We set out to specifically examine how varying the stereochemistry of the BOG group and the length of the lipid chain affect lymphatic uptake. Additionally, one known shortcoming of lipid prodrugs such as **TXL** is their susceptibility to metabolic oxidation at the terminal methyl group.^40^ To mitigate premature metabolism, we sought to functionalize the ω position of the lipid moiety with a CF_3_ group, to bolster stability against ω-oxidases. The success of this strategy was reported previously by our collaborative research groups.^40^ Consequently, we synthesized four representative BOG prodrugs of **TFV** featuring either HDP or ODP lipid tails in both stereoisomeric BOG forms.

As shown in Scheme 2, synthesis of BOG lipid moieties began with a Williamson etherification, under phase-transfer conditions, between commercially available (*S*)-(+) or (*R*)-(−)-2,2-dimethyl-1,3-dioxolane-4-methanol **1** and 1-bromohexadecane or 1-bromooctadecane to form etherified intermediates **2a**–**d**. Subsequent deprotection of the acetonide group under acidic conditions efficiently yielded 1,2-diols **3a**–**d**. The resultant primary alcohol was then protected with TBSCl in regioselective fashion to cleanly afford **4a**–**d**, followed by benzylation of the remaining secondary alcohol to produce **5a**–**d**. Subsequent fluoride-mediated removal of the TBS protecting group generated the desired BOG-functionalized lipid motifs **6a**–**d**, which were ultimately coupled to **TFV** utilizing DCC in the presence of catalytic DMAP to afford **8a**–**d** featuring HDP or ODP lipid chains.

**Scheme 2:**
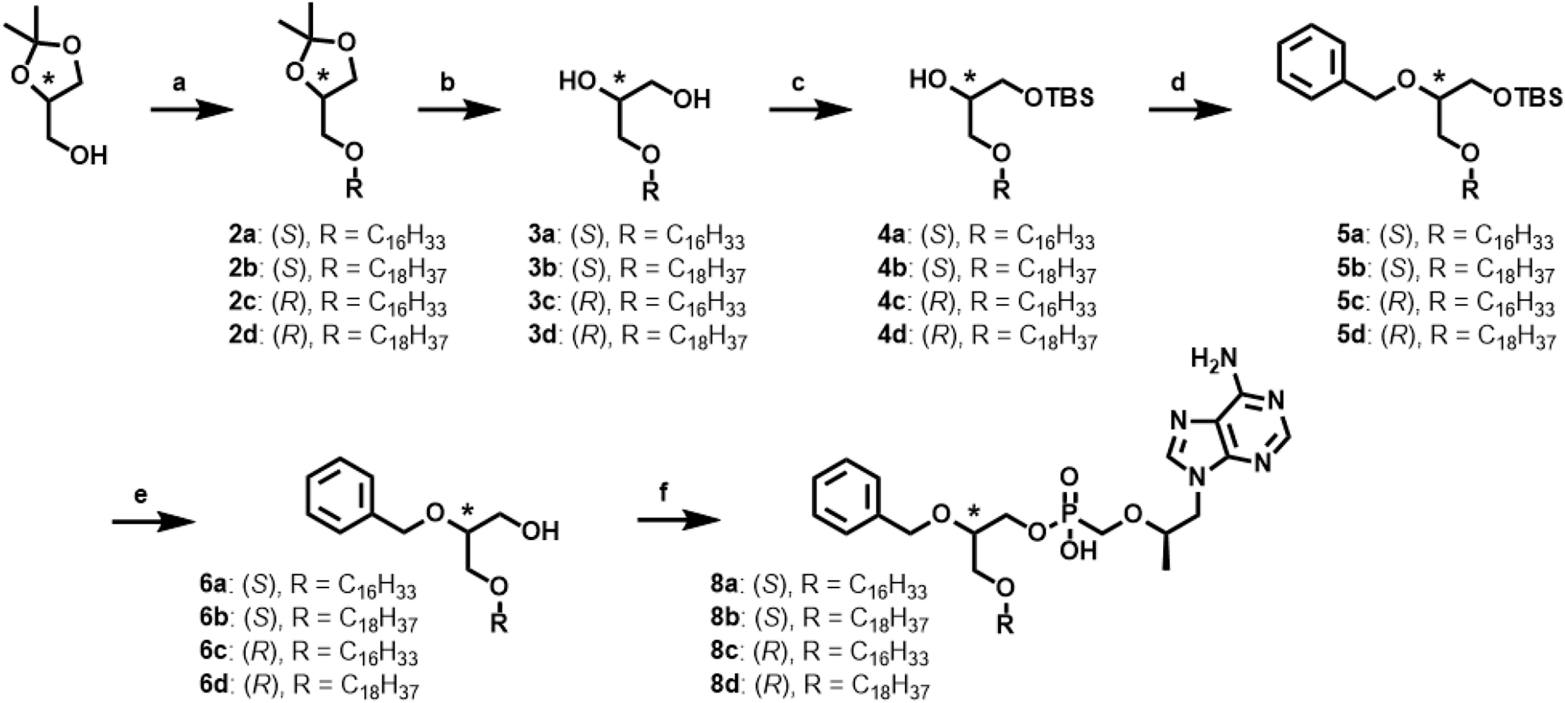
Synthesis Strategy of BOG TFV Prodrugs^a^

a Reagents and conditions: (a) 1-bromoalkane, TBAB, 50% NaOH (aq), THF, 75 °C, 20–24 h, 74–88% yield; (b) 80% AcOH (aq), 60 °C, 24–72 h, 79–90% yield; (c) TBSCl, Et_3_N, DMAP, DCM, rt, 19–24 h, 67–82% yield; (d) BnBr, NaH, THF, 0 °C to rt, 20–24 h, 56–72% yield; (e) HF-pyridine, pyridine, THF, rt, 20–24 h, 65–83%; (f) **7**, DCC, DMAP, NMP, 100°C, 20–24 h, 27–42% yield.

We next focused on the synthesis of second-generation **TFV** prodrugs incorporating a terminal CF_3_ group. Early *in vitro* profiling of **8a**–**d** suggested minimal differences between the (*R*) and (*S*) BOG stereoisomers. Therefore, for the next series of compounds, we focused only on the (*S*) form. In addition, we remained curious about the effect of lipid chain length, and thus, we included ω-functionalized analogs with both 16- and 18-carbon linear chains, matching the previously described ω-unfunctionalized analogs. We also wanted to understand the role of the BOG motif in driving the favorable tissue distribution profile observed for ODBG-CDV. Therefore, we sought to directly compare ω-functionalized **TFV** prodrugs with and without the BOG group. While the synthesis of **15a** was previously reported by our interdisciplinary research team,^40^ we describe below a slightly modified route, along with the synthetic methods used to prepare **15a**–**b** (Scheme 3) and **21a**–**b** (Scheme 4).

**Scheme 3:**
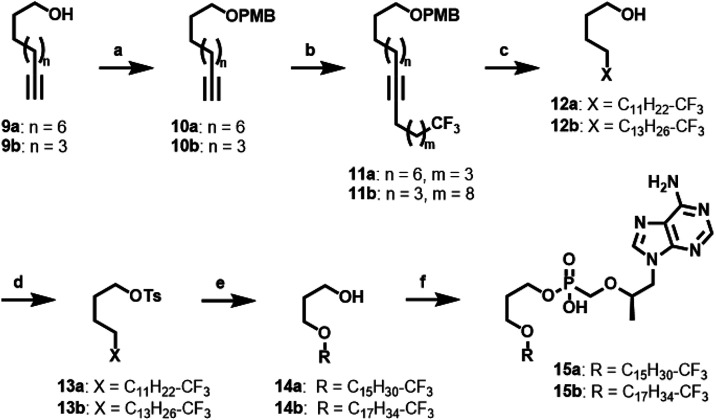
Synthetic Strategy to Produce ω-Modified **TFV** Prodrugs without BOG Motif^a^

a Reagents and conditions: (a) PMBCl, NaH, THF, rt, 22–24 h, 57–60% yield; (b) *n*BuLi, DMPU, THF, 1,1,1-trifluoro-5-iodopentane or 1,1,1-trifluoro-10-bromodecane, −78 °C to rt, 18–20 h, 41–62% yield; (c) Pd­(OH)_2_ on carbon, H_2_, EtOAc, rt, 18–22 h, 65-83% yield; (d) TsCl, Et_3_N, DMAP, DCM, 0 °C to rt, 16–18 h, 75–88% yield; (e) 1,3-propanediol, NaH, DMF, 0 °C to rt, 18 h; 84% yield; (f) **7**, DCC, DMAP, NMP, 100 °C, 20–24 h, 49% yield.

**Scheme 4:**
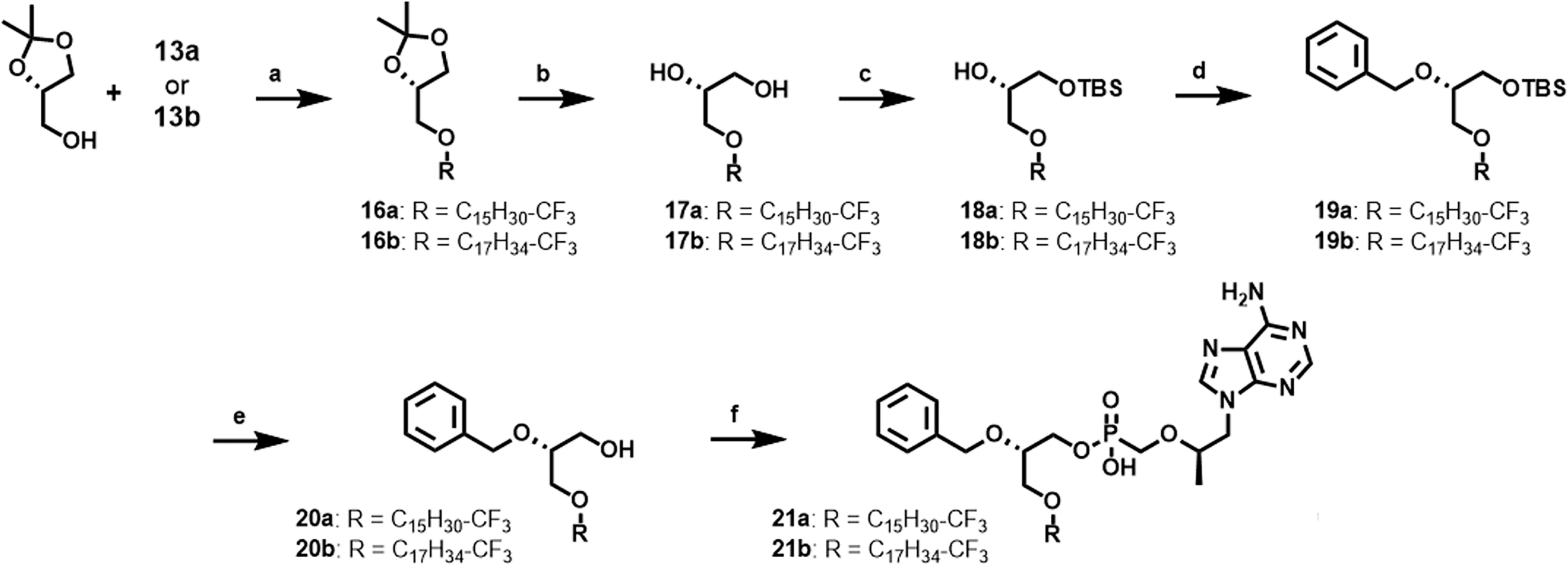
Synthesis Strategy of ω-Modified BOG **TFV** Prodrugs^a^

a Reagents and conditions: (a) TBAB, 50% NaOH (aq), THF, 75 °C, 21–24 h, 56–59% yield; (b) 80% AcOH (aq), 60 °C, 24–72 h, 58–64% yield; (c) TBSCl, Et_3_N, DMAP, DCM, rt, 22–24 h, 55–73% yield; (d) BnBr, NaH, THF, 0 °C to rt, 18–21 h, 52–69% yield; (e) HF-pyridine, pyridine, THF, rt, 22–24 h, 49-63% yield; (f) 7, DCC, DMAP, NMP, 100 °C, 20–24 h, 26–34% yield.

To generate intermediates **13a**–**b**, we began by protecting the primary alcohols of commercially available synthons **9a**–**b** to afford corresponding PMB ethers **10a**–**b**. *n*-Butyllithium-mediated alkylation of the terminal alkyne with a commercially available ω-CF_3_-functionalized alkyl halide yielded **11a**–**b**. Hydrogenation of the resulting internal alkyne was achieved in the presence of Pearlman’s catalyst under an atmosphere of H_2_ gas. These conditions also attractively enabled simultaneous removal of the PMB protecting group, resulting in ω-CF_3_-functionalized alcohols **12a**–**b**. In preparation for the penultimate step, the primary alcohol of synthon **12** was activated with TsCl to yield **13a,b**. To form **15a**–**b**, which lack the BOG moiety, **13a**–**b** underwent Williamson etherification with 1,3 propanediol to afford **14a**–**b**. Final DCC-mediated condensation reaction between **14a**–**b** and **TFV** yielded the desired lipid prodrugs **15a**-**b** (Scheme 3). As shown in Scheme 4, to generate ω-CF_3_ prodrugs incorporating the BOG motif, **13a**–**b** instead underwent Williamson etherification with starting chiral synthons (**1**), generating intermediates **16a**–**b** in 56–59% yield. **16a**–**b** were then carried through the same synthetic sequence outlined in Scheme 2 to produce the desired ω-functionalized BOG lipid prodrugs **21a**–**b**.

### *In Vitro* Profiling of Modified and Unmodified BOG Prodrugs Revealed Improved Metabolic Stability and Antiviral Activity Compared to TXL

With these prodrugs synthesized, we proceeded to evaluate their metabolic stability, cellular toxicity, and anti-HIV activity *in vitro*. Table 2 provides a comparative analysis of the pharmacological properties of **TXL** and our terminally modified and unmodified BOG derivatives. *c* Log *D* (pH 6.5) is a particularly useful metric with which to compare the lipophilicities of these phosphonic acids at intestinal pH. To measure cytotoxicity, HeLa-derived TZM-bl cells^49,50^ were treated with serial dilutions of each drug, and viability was assessed after 48 h. Two methods were used: an ATP luminescence assay, which measures cellular ATP levels as an indicator of metabolic activity (compounds **8a**–**d**), and an MTT assay, which evaluates cell viability via mitochondrial enzymatic activity (compounds **15b**, **21a**, and **21b**). Compounds **15a** and **TXL** were evaluated in both assays and produced similar results, representing nice internal controls for the different methods. All compounds showed no cytotoxicity up to 1 or 10 μM, depending on the maximum concentrations tested for the ATP luminescence and MTT assays, respectively. Metabolic stability of these prodrugs was evaluated in human (HLM) and mouse (MLM) liver microsomes. In HLM, all functionalized lipid prodrugs demonstrated a significant improvement in metabolic stability compared to **TXL**. This is consistent with our previous reports of mitigated ω-oxidation with ω-CF_3_-functionalized prodrugs^40,42^ as well as with results from the Hostetler group demonstrating reduced ω-oxidation of terminally unfunctionalized BOG prodrugs in monkey liver S9 fractions.^73^ All prodrugs including **TXL** were found to be stable in MLM, providing important contextual insights for subsequent mouse fPK studies. Antiviral activity was measured using TZM-bl cells infected with an HIV-1 pseudovirus expressing Env proteins from the Z1800 M transmitted/founder clone E4.02 in the presence of varying drug concentrations, as previously reported.^40^ Viral inhibition was assessed by measuring luciferase activity, with lower luciferase levels indicating reduced HIV infectivity. Since this assay showed reasonably high variability across different cultures, **TXL** used as an internal assay control (Tables S1–S3). The experiments were conducted in two groups: compounds **8a**–**d** were tested alongside **TXL** in one assay, while compounds **15a**–**b** and **21a**–**b** were tested with **TXL** in a separate assay. To account for this variability, **TXL** is reported as a range, and the fold-change for each compound is calculated using the **TXL** value from the corresponding experiment. It is notable that this variability is unrelated to the serum albumin formulation, as shown in Table S4. Additional details on these experiments can be found in the Supporting Information. All tested compounds were found to have similar potency to **TXL** in their respective assays. Although compounds **15a** and **15b** did not exhibit strong potency in our assay (IC_50_ = 1.6 μM and 0.6 μM, respectively), a previous study using HEK293T cells reported a significantly better antiviral activity for **15a** (IC_50_ = 0.05 μM).^40^ The discrepancy between results may be attributed to differences between HEK293T and TZM-bl cells. TZM-bl cells express the HIV coreceptors CCR5 and CXCR4, providing a model that closely mimics HIV entry into human cells, whereas HEK293T cells lack these receptors and rely on alternative pathways for viral entry.^51^ These differences likely account for the observed variability between assays. Finally, in our pseudoviral assay, compounds **21a** and **21b** were 5- and 3-fold more potent than **TXL**, respectively. These results confirm that our synthesized compounds retain anti-HIV activity and demonstrate a favorable safety and metabolic stability profile in these *in vitro* systems.

**Table 2:**
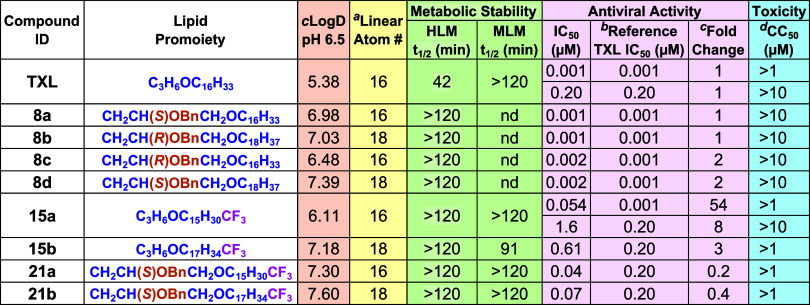
*In Vitro* Activity Profiles and *c* Log *D* (pH 6.5) of ω-Modified and Unmodified BOG Prodrugs of **TFV** ^e^

a Linear Atom # refers to the length of the lipid chain bound to the glycerol (**8a**–**d**, **21a**–**b**) or the oxypropyl (**TXL**, **15a**-**b**) backbone.

b Compounds were evaluated for antiviral activity across 2 different batches of cells that gave variable potencies across replicates (see Tables S1–S3), **as** highlighted here by **TXL** and **15a**.

c Fold change relative to TXL was accordingly used as the antiviral metric of interest.

d Cytotoxicity was evaluated using either an ATP luminescence assay or MTT assay, each using different test concentration limits across experiments. HLM = human liver microsomes. MLM = mouse liver microsomes.

e *c* Log *D* values were calculated using Schrödinger software (LigPrep and QikProp).

### Membrane Dynamics Influence Prodrug Uptake into CMs

Having completed this initial prodrug screening, we next sought to evaluate their ability to incorporate into CMs *in vitro*. For a lysoglycerolipid prodrug (*e.g*., TXL and BCV) to be packaged into CMs, it must first be transported into intestinal enterocytes, like Caco-2 cells, by intercalating into the outer leaflet of the plasma membrane before flipping (either actively or passively) to the inner leaflet. Subsequent membrane desorption facilitates intraenterocyte diffusion to the ER and Golgi, where CM are produced. Accordingly, we hypothesized that, even though high lipophilicity should drive prodrug association with CMs, these low hydrophilicity compounds first need to get through the plasma membrane, which could create a kinetic barrier to CM incorporation. This concept aligns with findings from Hostetler’s work, where the superior antiviral activity of BCV compared to CDV *in vitro* could be attributed to enhanced persistence of active metabolites in human MRC-5 lung fibroblasts due at least in part to a membrane depot effect.^52^ As such, we designed an assay to investigate the distribution dynamics between cellular membranes and CMs of compound **8d**, the most lipophilic of the terminally unmodified prodrugs (*c* Log *D* pH 6.5 = 7.39). Proceeding with the CM production and isolation assay as described (Scheme 1), cells were harvested in parallel, and the total membrane fraction was separated from other cellular components via centrifugation through a specialized filter provided with the Minute Plasma Membrane Protein Isolation and Cell Fractionation Kit from Invent Biotechnologies (cat No. SM-005). Drug concentration in total membrane and CM fractions were analyzed after extraction at 2, 8, and 20 h postincubation initiation (*n* = 6 biological replicates). Results shown in Figure 7b indicate that at 2 h, most of the drug remained sequestered in the cellular membranes with minimal presence in CMs. By the 8 h time point, there was a noticeable shift in drug distribution, with more drug moving into the CMs. And by 20 h, drug was undetectable in the membrane fraction, coinciding with a peak in CM concentration, as later measurements at 24 and 30 h demonstrated a decline in CM drug levels (Figure S2).

**Figure 7:**
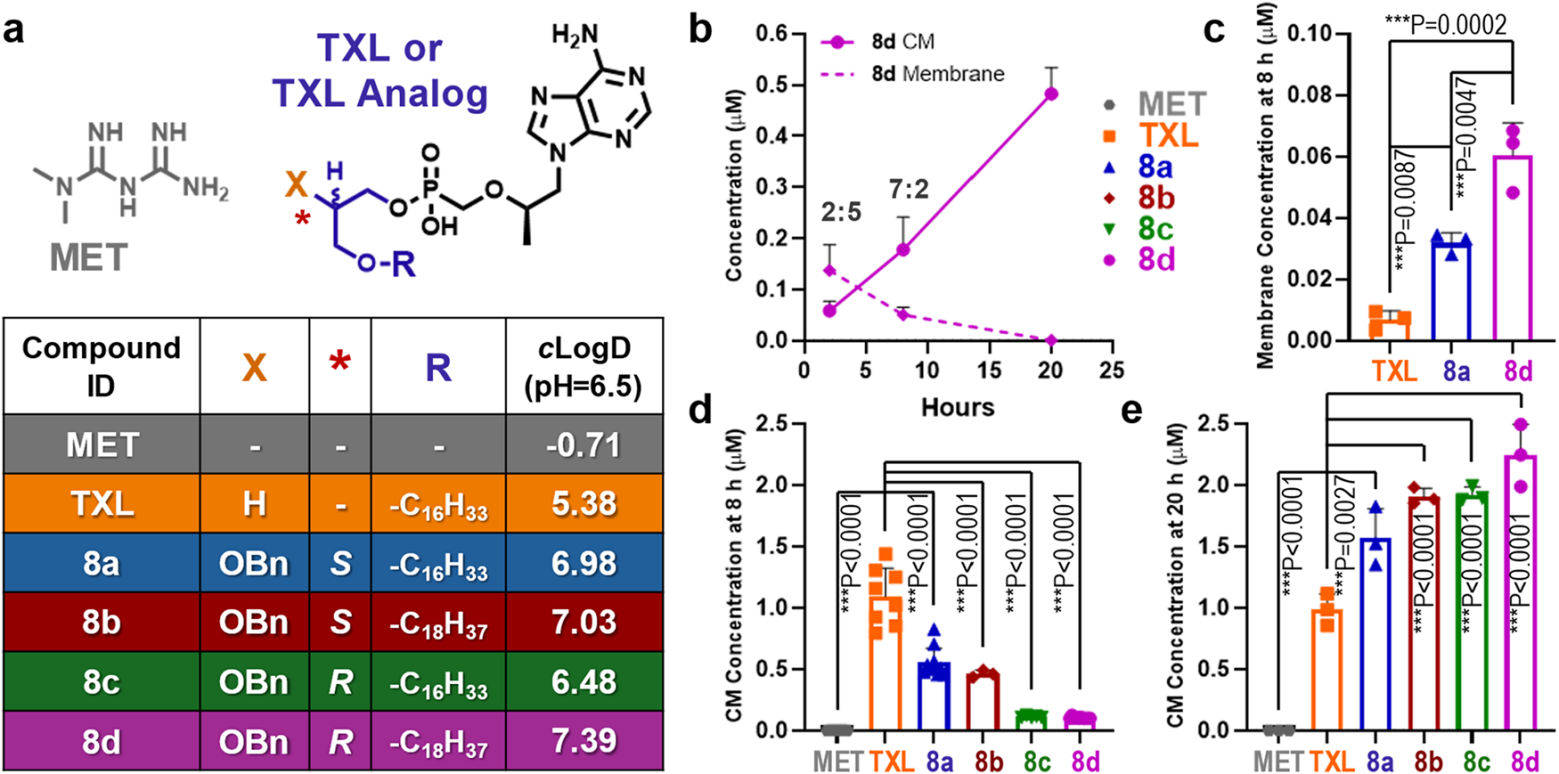
Time-dependent diffusion of prodrugs into CM. (a) Structures and *c* Log *D* values of negative control **MET** and TFV prodrugs with variable **X**, *, and **R** motifs that were tested in the CM assay. (b) Concentration of compound **8d** in Caco-2 cell total membrane and CM fractions at 2, 8, and 20 h (*n* = 3). Ratios represent drug concentration in CM relative to membrane. (c) Membrane concentrations of HDP and ODP BOG prodrugs compared to **TXL** at 8 h (*n* = 3). (d) CM packaging of **TXL** and BOG prodrugs at 8 h; **8a** (*n* = 10), **8b** (*n* = 3), **8c** (*n* = 5), **8d** (*n* = 10), **TXL** (*n* = 8), **MET** (*n* = 8). (e) Concentration of all prodrugs at 20 h following complete diffusion out of the membrane. Data represent the mean ± SD. Statistical comparisons were made using 1-way ANOVA. *c* Log *D* values were calculated using Schrödinger software (LigPrep and QikProp). Graphics were created with GraphPad Prism v10.

This observation indicates significant membrane retention, which is another key feature of these lipid conjugates. To investigate this further, we used three compounds with varying lipophilicities to assess their relative membrane sequestration. We selected the 8 h time point based on the time course assay, which showed measurable drug in both membrane and CM compartments. Two BOG prodrugs featuring different lipid chain lengths (**8a**, **8d**) were compared with **TXL** (*n* = 3 biological replicates per compound). Results showed that at the 8 h time point, the highest lipophilicity compound **8d** had significantly greater concentrations in the membrane than **TXL** (Figure 7c). This finding suggests that sequestration in the membrane hindered the immediate incorporation of the drug into CMs, highlighting dependence on time. While the *in vivo* implications of this membrane depot effect certainly require further investigation, it is tempting to speculate that these dynamics could be optimized for maximum systemic exposure over time with repeat oral dosing.

### Lipophilicity Trends with CM Incorporation Overtime

At 8 h, our initial analyses indicated that the more lipophilic prodrug was retained in the membrane, while at 20 h, no detectable drug remained in this compartment. Extending the time course to 30 h confirmed that drug concentration in CMs had peaked at 20 h (Figure S2). Accordingly, we screened all terminally unmodified BOG prodrugs for uptake into CMs at 8 and 20 h. Each experiment included **MET** as a negative control. Drug quantities in CM extract from Caco-2 cells exposed to **TXL** (*n* = 8), **8a** (*n* = 10), **8b** (*n* = 4), **8c** (*n* = 5), **8d** (*n* = 10), and **MET** (*n* = 8) are shown in Figure 7d,e. At 8 h (Figure 7d), **TXL**, the least lipophilic of the test compounds, exhibited significantly greater concentrations in CMs than all BOG substituted prodrugs. This indicates that increased prodrug lipophilicity enhances membrane retention, which may delay initial incorporation into CMs. By 20 h, however, a shift in the distribution was observed, with BOG variants showing higher concentrations in CMs compared to **TXL** (Figure 7e). Plotting the prodrug’s *c* Log *D* against the observed concentration in CMs at the 20 h time point revealed a strong positive correlation (*R* ^2^ = 0.80, Figure 8c), consistent with the hypothesis that increasing lipophilicity enhances prodrug incorporation into CMs.

**Figure 8:**
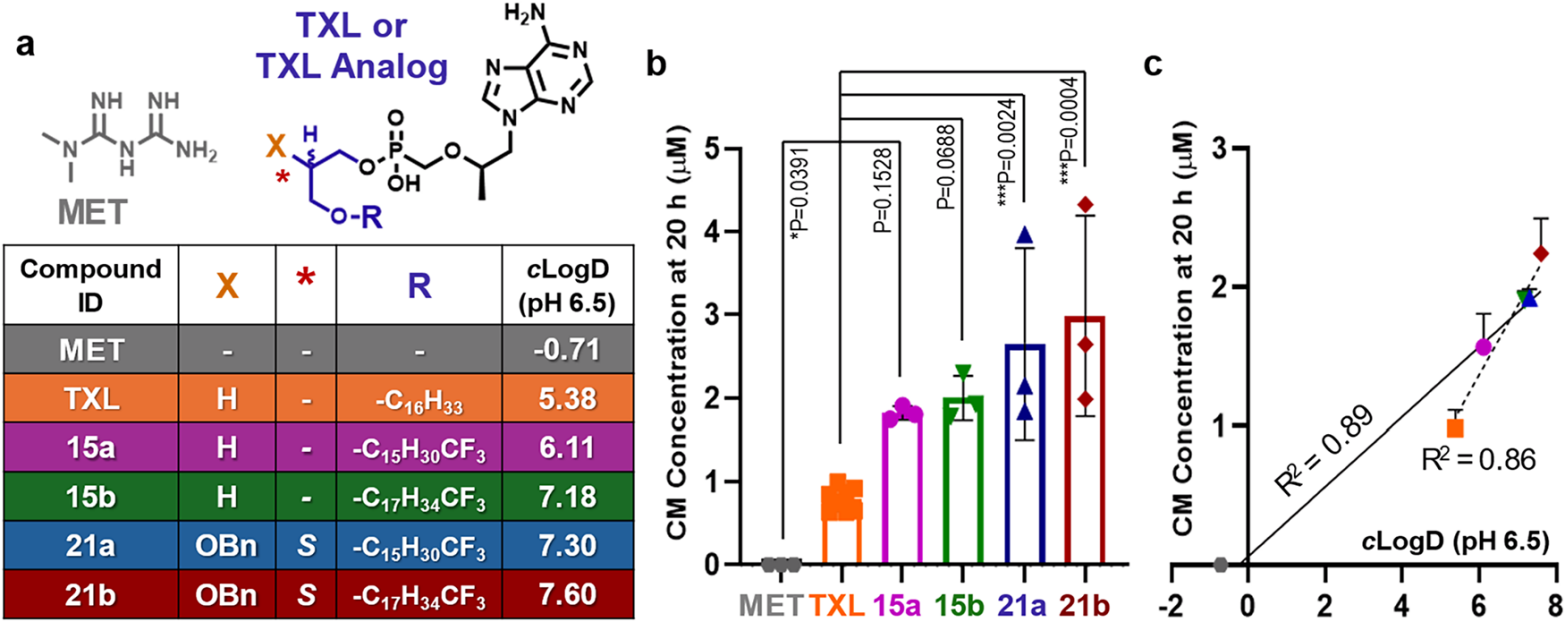
Terminally modified lipid prodrugs exhibit superior CM packaging *in vitro*. (a) Structures and *c* Log *D* values of negative control **MET** and TFV prodrugs with variable **X**, *, and **R** motifs that were tested in the CM assay. (b) **MET** and prodrug quantities in CM extracts from cells exposed to **TXL** (*n* = 8), TXL analogs (*n* = 3), and **MET** (*n* = 3) after 20 h. (c) Correlations between *c* log *D* and prodrug concentration in CMs at 20 h. Data represent the mean ± SD. Statistical comparisons were made using 1-way ANOVA. *c* Log *D* values were calculated using Schrödinger software (LigPrep and QikProp). Graphics were created with GraphPad Prism v10.

### Of the ω-Functionalized Lipids, BOG-Modified Variants Demonstrate Superior Packaging into CMs at 20 h Relative to Those Lacking the BOG Modification

After characterizing the impact of membrane dynamics on the packaging of ω-unfunctionalized prodrugs into CMs, we proceeded to screen the ω-CF_3_-functionalized BOG lipid prodrugs in our CM assay. All collections were done at the 20 h time point to allow full diffusion of prodrug out of the membrane. Results are shown in Figure 8. Consistent with trends observed in the ω*-*unmodified prodrugs, ω-CF_3_–BOG prodrugs **21a** and **21b** exhibited significantly higher CM concentrations at the 20 h time point, with **21a** showing a 3.3-fold and **21b** a 3.8-fold increase compared to **TXL** across 3 biological replicates. As expected, **21a** and **21b** showed higher CM concentrations than **15a** and **15b**, which lack the BOG motif, although this increase did not reach statistical significance. However, CM concentration strongly correlated with *c* Log *D* values (*R* ^2^ = 0.89, Figure 8c), consistent with trends observed for ω-unfunctionalized prodrugs and highlighting the role of lipophilicity on CM incorporation. Interestingly, the BOG-modified prodrugs **21a** and **21b** exhibited CM concentrations that were approximately 1.8-fold and 1.6-fold higher than **8a** and **8b**, respectively. This observation aligns with the increased lipophilicity conferred by fluorination, suggesting the combination of BOG and CF_3_ functionalities work synergistically to enhance CM uptake. This is further supported by the 2.4-fold increase in CM concentration observed for **15a** compared to **TXL**, where the only difference is the inclusion of the terminal CF_3_ group. With these results established, we wanted to ensure that prodrug association with CM was not an artifact of the CM production and isolation process. Therefore, as an important control experiment, Caco-2 cells were incubated with 10 μM of TXL or compound **21a** (*n* = 6 per group) in the presence of the lipid mixture for 15 min at 37 °C. After incubation, the cells were washed three times with PBS, and fresh drug-free growth media was added. Aliquots of the media were collected at 0, 5, and 15 min postwash. Samples were analyzed by LC-MS/MS for the presence of TXL and **21a**. At all time points, prodrug concentrations in the media were below the limit of detection, indicating that passive diffusion or release of membrane-associated drug into the media did not occur as an artifact under these conditions. Overall, these findings suggest that ω-CF_3_ and BOG functionalities serve complementary roles in promoting CM uptake *in vitro*, motivating further investigation into how these results translate *in vivo*.

### Terminally Modified BOG Prodrugs Exhibit Superior Tissue Distribution Compared to TXL

Given the increased packaging of the CF_3_-modified lipids into CMs compared to their ω-unmodified congeners, we selected these compounds for PK studies in mice. We hypothesized that, following oral administration, our modified prodrugs would favor CM-mediated transport into the lymphatics over absorption into mesenteric veins feeding into the portal system. By circumventing direct transport to the liver, these prodrugs are expected to achieve elevated levels in the lung relative to the liver, as lymph empties into the jugular venous angle and mixes with blood en route to the pulmonary arteries (Figure 1). For all mouse PK experiments, prodrugs were formulated in a 9:1 mixture of olive oil and ethanol, then administered to male C57BL/6 mice via oral gavage at a dose of 10 mg/kg. Organs (liver, lung, and plasma) were harvested at 1, 2, 4, 8, and 18 h postdose, per the protocol described by our groups previously.^40^ Concentrations of prodrug were quantified using LC-MS/MS after tissue homogenization, extraction, and sample preparation. Results are shown in Figure 9 and Table 3.

**Figure 9:**
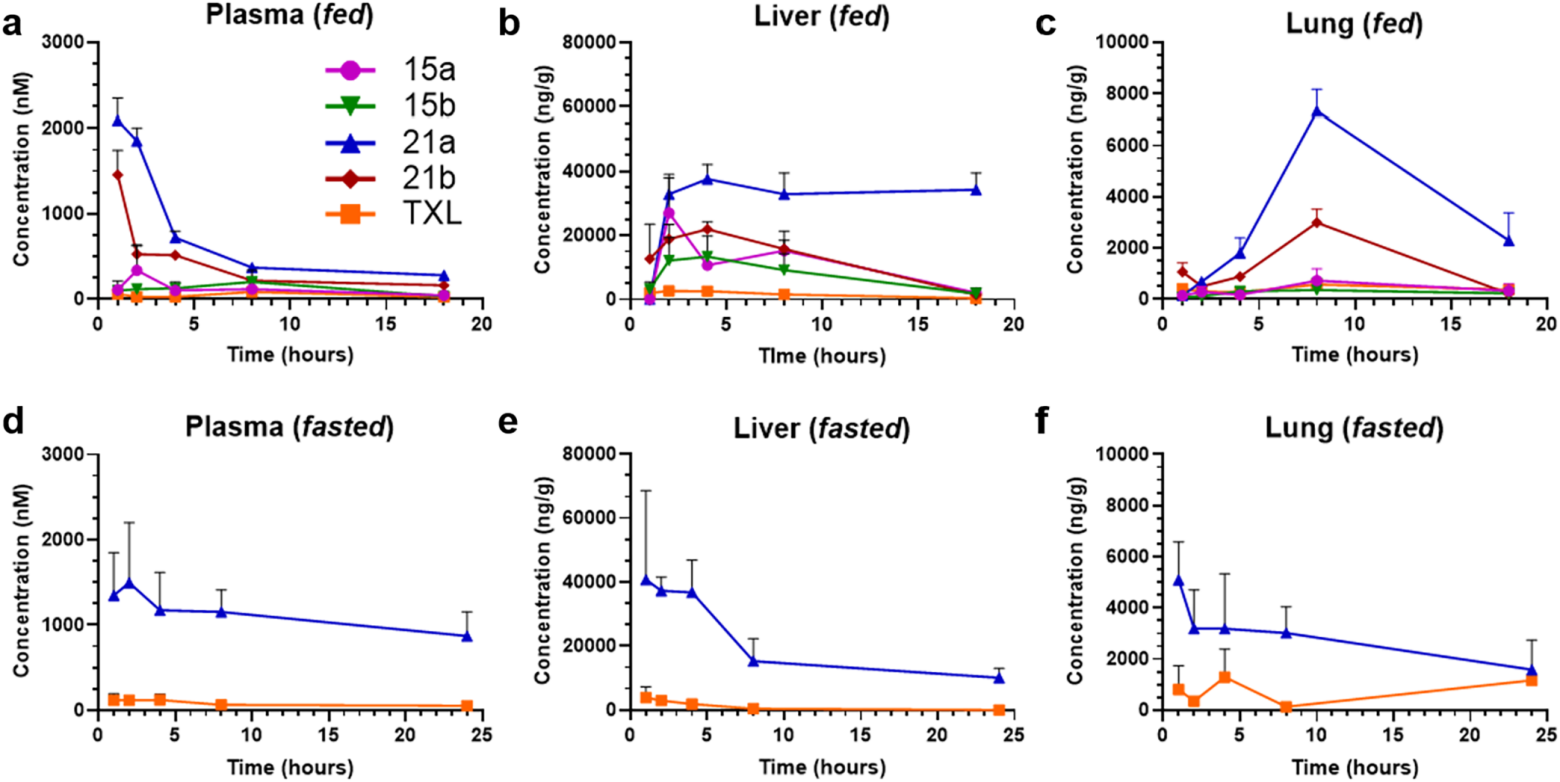
Terminally modified prodrugs show enhanced tissue distribution and PK *in vivo* in both fasted and unfasted mice. (a–c) Concentrations of prodrugs in (a) plasma, (b) liver, and (c) lung following a single oral dose in fed mice (*n* = 4). (d–f) Comparison of tissue distribution between compound **21a** and **TXL** in fasted mice, showing levels in (d) plasma, (e) liver, and (f) lung (*n* = 4). All mice were dosed at 10 mg/kg using a vehicle composed of 9:1 olive oil to EtOH. Data are means ± SEM. Figures were created with GraphPad Prism v10.

**Table 3:**
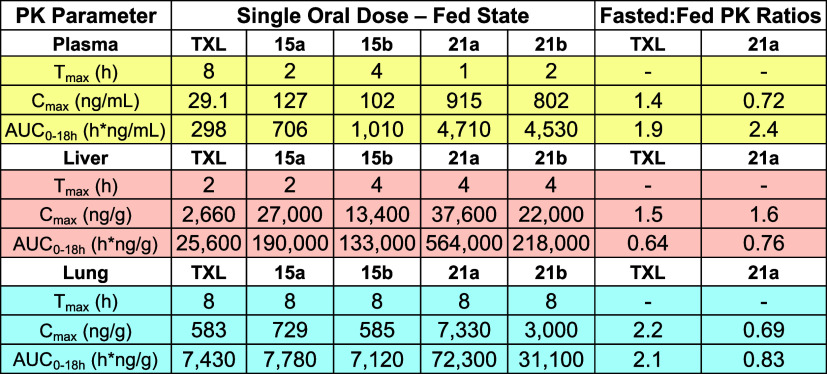
Mouse Oral PK Profiles^a^

a PK properties of ω-functionalized prodrugs after a single oral dose were compared with **TXL** in both the fasted and fed state. Fasted:fed represents the ratios of AUCs in tissues obtained in the fasted compared to the fed state.

The ω-CF_3_–BOG-lipid prodrugs (**21a** and **21b**) demonstrated superior absorption and systemic exposure compared to **TXL** (Table 3). Specifically, **21a** exhibited a 16-fold increase in plasma AUC and a 32-fold increase in *C*_max_, while **21b** showed a 15-fold increase in AUC and a 28-fold increase in *C*_max_, relative to **TXL**. A similar trend was observed when comparing the ω-CF_3_–BOG prodrugs (**21a** and **21b**) to their non-BOG counterparts (**15a** and **15b**). For **21a** versus **15a**, which lacks the BOG group, plasma AUC and *C*_max_ increased 7-fold. Similarly, **21b** showed a 5-fold increase in plasma AUC and 8-fold increase in *C*_max_ compared to **15b**, suggesting the BOG motif plays a role in increasing bioavailability. Importantly, the difference in plasma levels observed between **21a**–**b** and **TXL** were more pronounced than between **21a**–**b** and **15a**–**b**, further supporting earlier findings that the ω-CF_3_ contributes to the overall improved PK properties of these prodrugs. While is it difficult to project, based on the available data, how these mouse PK profiles will translate to systemic exposure in humans, the early plasma *T*_max_ values observed in both fed (Figure 9a) and fasted (Figure 9d) states raises an interesting point for discussion. This early surge in plasma levels is likely due to absorption mechanisms that are independent of CM packaging and lymphatic transport. While lymphatic transport is a property of these lipid conjugates, it is not the only absorption mechanism at play. Early *T*_max_ values suggest that a fraction of each orally dosed compound undergoes rapid absorption into plasma, likely through the hepatic portal vein, whereas the sustained levels over time are more representative of the lymphatically absorbed fraction, whose path to systemic circulation is much slower. Furthermore, this partitioning between hepatically directed and lymphatically directed fractions is known to vary as a function of food intake, which could at least partially explain the differences in the plasma PK profiles of **21a** between Figure 9a,d. However, further experimentation is required to fully elucidate the fundamental mechanisms governing these mouse PK profiles, especially with regard to human translation.

In terms of tissue distribution, both CF_3_-modified BOG prodrugs (**21a** and **21b**) exhibited elevated lung concentrations compared to **TXL** and **15a**–**b**. **21a** exhibited an AUC_0–18h_ of 72,300 h·ng/g in lung tissue, representing a 10-fold improvement over **TXL** (7,430 h·ng/g) and a 9-fold improvement over **15a** (7780 h·ng/g). The similarity in lung AUC values between **15a** and **TXL** suggests that the BOG motif is the predominant driver of this enhancement in lung delivery. Supporting this, **21b** demonstrated a 4-fold increase in lung AUC compared to both **15b** and **TXL**, further highlighting the role of the BOG motif in driving improved lung delivery. Notably, **21a** with the shorter HDP chain outperformed **21b** with the longer ODP chain in terms of lung delivery. This aligns with findings from the Porter group showing that lymphatic transport increases with the lipophilicity of the monoglyceride species, peaking at a 16-carbon chain length, with a decline observed for 18-carbon and 21-carbon analogs.^14^ Crucially, *C*_max_ values in the lung for all prodrugs far exceeded their respective IC_50_’s, suggesting that these prodrugs reach the lung at concentrations sufficient for therapeutic efficacy.

Contrary to observations made by Hostetler et al.,^18^ our data reveal the liver to be the tissue containing the highest prodrug concentration across all compounds (Figure 9). This suggests that, while increasing lipophilicity can bias absorption toward the lymphatics, the liver remains a key site for drug sequestration. It is important to point out that the use of olive oil in the oral gavage vehicle may have influenced tissue distribution to some extent. However, this study was designed to compare PK profiles between different compounds, and all compounds evaluated *in vivo* were administered using the same method with the same dose in the same vehicle. Given the high liver concentrations relative to the lung, the lung-to-liver ratios provide limited insight here into selective targeting. However, it is notable that the CF_3_-modified BOG prodrugs achieved significantly greater lung exposure than **TXL** at the same oral dose, demonstrating their effectiveness in reaching this target compartment, despite considerable hepatic extraction.

### **21a** Maintains Superior Exposure and Lung Distribution to TXL in Fasted Conditions

It is well established that oral absorption and subsequent tissue distribution of lipophilic drugs can be affected by an animal’s fed state.^53–55^ Food intake serves as a stimulus for CM production in enterocytes, and therefore, variable fed states contribute to differences in prodrug absorption into the lymphatics. For example, coadministration of antimalarial drug N-251 with a lipid emulsion was shown to enhance lymphatic transport by over 3-fold.^56^ To evaluate the impact of fasting on drug absorption and distribution, we modified our protocol to include an overnight fast. This mouse PK experiment was performed as above, with the final time point extended to 24 h (instead of 18) for logistical considerations. We focused on compound **21a** due to its superior performance in the first PK study, with **TXL** serving as the benchmark as before. Results are shown in Figure 9 and Table 3. Interestingly, even in the absence of food, the trends observed previously remained consistent, with **21a** displaying significantly increased exposure levels compared to **TXL**, as evidenced by higher *C*_max_ in plasma, liver, and lung and statistically significant increases in AUC.

Comparing the PK profiles of **TXL** under fasted and fed conditions, we observed a 2-fold increase in lung AUC in fasted mice (15,600 h·ng/g fasted, 7430 h·ng/g unfasted). Interestingly, this trend differed for **21a**, which showed a 17% decrease in lung concentration under fasted conditions (59,900 h·ng/g fasted, 72,300 h·ng/g unfasted). This divergence may be explained by the lipophilicity of **21a**, suggesting that lymphatic absorption for this compound is more dependent on food intake and subsequent CM production. In contrast, absorption of the less lipophilic **TXL** appears to improve under fasted conditions. These findings are consistent with a study examining the effect of food on PK properties of various oral antineoplastic drugs which reported that, for compounds with Log *P* > 4.34, drug absorption in the fed state positively correlates with Log *P*.^57^

Finally, we observed earlier *T*_max_ in both **TXL** and **21a** in fasted mice. There is no clear consensus in the literature on the impact of the fed state on *T*_max_. While many studies report no significant difference,^58–60^ others illustrate faster absorption of lipophilic drugs in the fed state.^61^ This discrepancy suggests that the impact of fed state on *T*_max_ is highly drug-dependent. The previously mentioned study on antineoplastic drugs found considerable variability in *T*_max_; some drugs had an earlier *T*_max_ in fasted states, while for others it was delayed.^57^ For **TXL**, the *T*_max_ shifted from 8 h (fed) to 4 h (fasted), while for the more lipophilic **21a**, the shift was even more pronounced, from 8 h (fed) to 1 h (fasted). Earlier *T*_max_ values may be attributed to faster gastric emptying, which has been shown to be under 30 min in the absence of food and approximately 120 min in the fed state.^54^ Taken together, these results demonstrate that the ω-CF_3_-modified BOG prodrug **21a** demonstrates superior systemic drug exposure and lung distribution than **TXL**, even in the absence of food.

### **21a** Substantially Suppresses the Release of TFV in Plasma and Liver Relative to TXL

To further evaluate the *in vivo* performance of **21a**, we measured the release of the parent drug, **TFV**, in plasma (Figure 10a), liver (Figure 10b), and lung tissues collected from our PK study (fed state), and compared these levels (Figure 10c) with **TXL**. This characterization of systemic exposure to **TFV** is critical, since premature release is associated with organ-specific and dose-limiting toxicities.^30,62^ In **TXL**-dosed mice, higher levels of **TFV** (*C*_max_ and AUC) were detected in plasma and liver, relative to **21a**-treated mice. This is consistent with our previous reports of ω-functionalization as a useful tactic to suppress hepatic ω-oxidation. Also notable is the sustained **TFV** release in liver and plasma over time with both **TXL** and **21a**, highlighting one of the important benefits of strategic lipid conjugation. This kinetic profile may reduce premature systemic release of **TFV**, thereby limiting associated adverse events, while simultaneously supporting longer dosing intervals to increase the patient compliance barrier to resistance. These data also support the hypothesis that the BOG motif meaningfully influences tissue absorption, distribution, and/or metabolism in physiological systems, setting a foundation for future platform application and optimization. Finally, TFV was interestingly undetectable (<1 ng/mL) in lung tissue for both prodrugs using the indicated mass spectrometry methodology, suggesting altered metabolic activation kinetics in this compartment. As the lung is a privileged compartment that plays host to many different diseases, deciphering the driving forces behind this surprising result is the subject of ongoing research including more sensitive mass spectrometry approaches.

**Figure 10:**
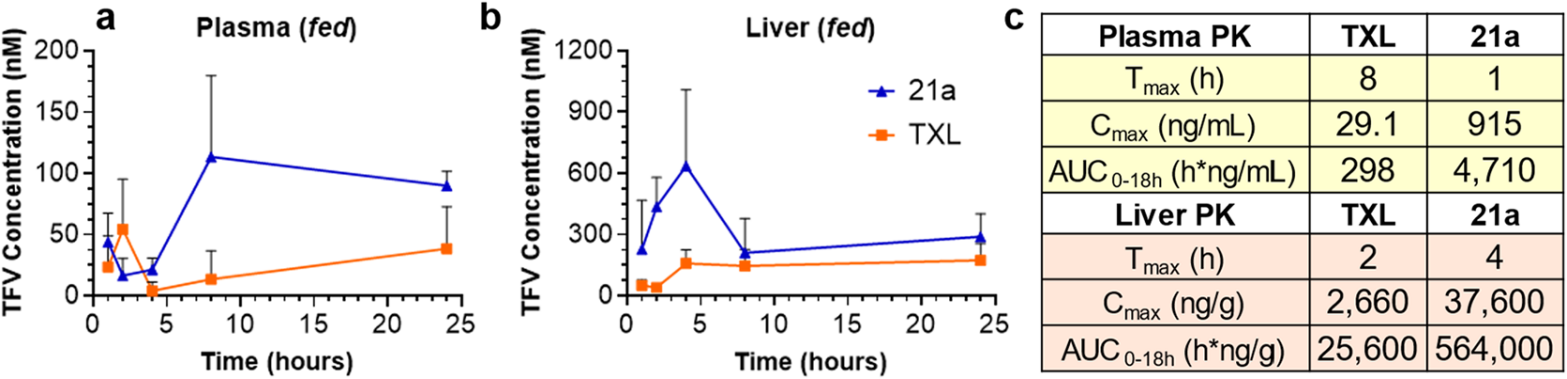
Quantification of TFV metabolite in tissues of mice treated with either **TXL** or **21a**. Concentration–time profiles of parent TFV in (a) plasma and (b) liver of mice treated with either **TXL** or **21a** (*n* = 3 biological replicates). (c) Summary of key TFV plasma and liver PK parameters following oral dose of **TXL** or **21a**. Data represent the mean ± SEM. Graphics were created with GraphPad Prism v10.

## Conclusions

This study combines mechanistic insights into the oral absorption and distribution patterns of lipophilic compounds with innovations in prodrug design to produce a strategy for targeted drug delivery via the CM incorporation. Here we identified two key structural improvements over the prototypical lipid prodrug **TXL**: a CF_3_ group at the terminal position to protect against early oxidation in the gut and liver, and the incorporation of a BOG motif, both of which increase lipophilicity. We developed an assay to characterize the packaging of various control drugs into Caco-2-derived CMs *in vitro*, and then applied our prodrugs to evaluate how structural modifications influence CM packaging. This cell-based screening approach allows efficient evaluation of multiple compounds for their intestinal lymphatic system-targeting potential while reducing cost, time, and reliance on animal studies. We found correlation between *c* Log *D* and prodrug concentration in CMs, with ω-CF_3_ and BOG functionalities acting synergistically to enhance CM association *in vitro*. PK studies demonstrated translation of these findings in mice, suggesting that prodrugs with increased CM uptake *in vitro* can exhibit improved lymphatic delivery *in vivo*. ω-CF_3_ prodrugs, both with and without BOG functionality, showed significantly higher plasma and tissue levels compared to **TXL**, suggesting that the CF_3_ group increases systemic absorption and/or mitigates metabolism. The BOG motif, however, emerged as the key determinant of preferential lung delivery, as ω-CF_3_–BOG prodrug **21a** achieved 10-fold higher lung concentrations than non-BOG prodrugs **15a** and **TXL**, which achieved similar levels in lung. Among the tested compounds, **21a**, the C_16_ ω-CF_3_–BOG prodrug, emerged as the lead candidate, demonstrating superior lung delivery and systemic exposure both under fed and fasted conditions (Figure 11). Importantly, both **21a** and **TXL** exhibited sustained release of TFV in plasma and liver, and **21a** generated substantially less TFV in these compartments, which may help reduce off-target toxicity and support extended dosing intervals. Overall, this study highlights the potential of lipid modifications to improve oral drug delivery of TFV and other nucleoside-based therapeutics via intestinal CM incorporation. Recent work by the Hostetler group on similar prodrug functionalization underscores the advantage of this approach.^38^ By promoting efficient CM incorporation, custom-tailored lipid prodrugs, such as those reported herein, could lead to better therapeutic outcomes, especially in the context of diseases requiring access to lymphatic compartments, such as HIV. The results reported here lay the groundwork for future optimization of lipid-based prodrugs, aiming to enable their oral bioavailability and efficacy in clinical settings.

**Figure 11:**
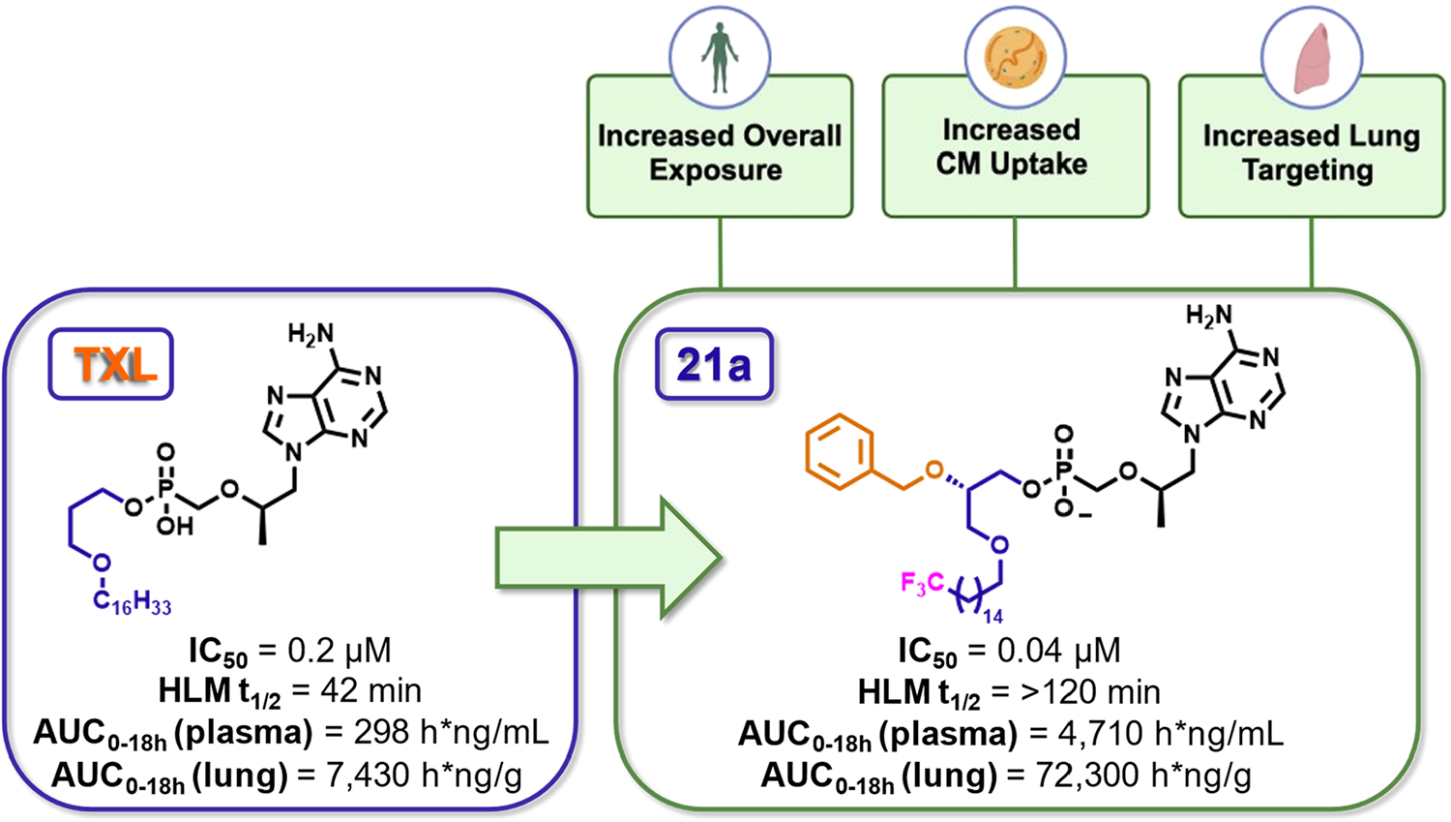
Summary of the pharmacological evaluation of ω-CF_3_-functionalized BOG prodrugs of HIV NRTI TFV, highlighting the superior PK properties compared to unfunctionalized TXL. HLM = human liver microsomes.

## Materials and Methods

### Bioanalytical and Pharmacological Procedures

#### Caco-2 Cells and Culture

Human epithelial colorectal adenocarcinoma (Caco-2) cells were obtained from ATCC (HTB-37) and maintained in Eagle’s minimum essential medium (EMEM, ATCC cat. No. 30–2003) supplemented with fetal bovine serum (20%) and penicillin-streptomycin (5%). Cells were initially seeded in 10 cm tissue culture plates and incubated at 37 °C under 5% CO_2_. They were subcultured upon reaching 75% confluence per ATCC protocol. For CM experiments, cells were plated in 6-well collagen-coated plates in 2 mL media and grown to confluence. Once confluent, growth media was changed every other day.

#### Chylomicron (CM) Biogenesis and Isolation (see Scheme 1)

At 14-days postconfluence, Caco-2 cells were incubated in a lipid mixture to induce secretion of CMs. For the lipid solution, oleic acid (50 mg), lecithin (40 mg), and sodium taurocholate (48 mg) were weighted out into an Eppendorf tube, and the volume was brought up to 900 μL with PBS, followed by vigorous vortexing to ensure sample homogeneity. This 100× lipid mixture was then diluted into growth media to achieve the desired concentrations (2 mM oleic acid, 1.36 mM lecithin, 1 mM sodium taurocholate) by adding 500 μL of the lipid mixture to 49.5 mL of media. The lipid-supplemented media was thoroughly mixed and sterilized by filtration using a 250 mL sterile vacuum filter. For the drug-exposed CM experiments, each test compound was dissolved in 2 mM human serum albumin (HAS, Sigma, essentially fatty acid free) in DPBS, then added to 10 mL aliquots of lipid-containing media to achieve a final concentration of 10 μM. Subsequently, lipid-containing media was added to Caco-2 cells and incubated for 4 h at 37 °C. After incubation, cells were washed twice with PBS then incubated for 4, 8, or 20 h at 37 °C in fresh, colorless growth medium. Media was collected into 15 mL falcon tubes and centrifuged at 2000*g* to remove cell debris. Postcentrifugation, the supernatant was transferred to 50 mL Nalgene Oak Ridge High-Speed PPCO Centrifuge Tubes. Next, to adjust the density for ultracentrifugation, 1.19 g of NaCl in 2 mL of deionized (DI) H_2_O were added to reach a final volume of 5 mL, corresponding to a density of 1.20 g/mL. This mixture was dissolved by vortexing and balanced by weight. 500 μL of DI H_2_O was carefully overlaid onto the mixture. Ultracentrifugation was performed for 2 h and 30 min at 10,920 relative centrifugal force (RCF). Immediately after centrifugation, the top 500 μL layer, containing CMs, was carefully collected using a gel-loading pipet tip.

#### Transmission Electron Microscopy (TEM) Analysis

All microscopy experiments were performed at and with the assistance of the Emory University Robert P. Apkarian Integrated Electron Microscopy Core Facility. For negative stain electron microscopy, samples were absorbed for 5 min onto freshly glow-discharged, carbon-coated, copper grids (Electron Microscopy Sciences, Hatfield, PA, USA). Excess fluid was then blotted from the grid surface with filter paper. The sample-adsorbed grid was placed, sample side down, onto a drop of a freshly prepared, filtered 2% uranyl acetate solution for 1 min. Excess stain was then blotted from the surface of the grid. Samples were imaged using a JEOL JEM-1400 transmission electron microscope (JEOL, Japan) operated at 80 kV. Electron micrographs were acquired on a 2048 × 2048 charge-coupled device (CCD) camera (UltraScan 1000, Gatan Inc., Pleasanton, CA, USA) at a nominal magnification of 30,000× (pixel size 3.4 Å per pixel).

#### Apolipoprotein B ELISA

The quantification of Apolipoprotein B (ApoB) in CM extract was performed using a commercially available ELISA kit (Abcam, Human Apolipoprotein B ELISA Kit, ab190806) according to the manufacturer’s instructions. Briefly, standards and samples were added to wells precoated with human ApoB antibody and incubated for 1 h to allow the binding of ApoB in the samples to the immobilized antibody. After three washes, a biotin-conjugated antihuman ApoB antibody was added. Following another wash to remove unbound biotinylated antibody, streptavidin conjugated to horseradish peroxidase (HRP) was added to the wells. After adding 3,3′,5,5′-tetramethylbenzidine, the color produced was measured photometrically at a wavelength of 450 nm and is directly proportional to the concentration of ApoB present in the samples. The sample values were then cross-reference against a standard curve to determine the respective concentrations of ApoB.

#### Drug Extraction from CM and Quantitation by Mass Spectrometry

To an aliquot of CM extract was added an equal volume of ACN into 1.5 mL Eppendorf tubes. Tubes were shaken for 1 h at rt then centrifuged at 15,000 RCF to remove debris. The clear supernatant was transferred to a glass vial and concentrated under reduced pressure to yield a dry solid. Sample was then reconstituted in 70% ACN in H_2_O, filtered and then analyzed. LC-MS/MS analysis was performed using Agilent Infinity II 1260 HPLC coupled to a G6460C QQQ MS. An Infinity Laboratories Poroshell 120 EC-C18 2.7 μM, 2.1 mm × 50 mm (Agilent, Santa Clara, CA, USA) analytical column was used for chromatographic separations. Two mobile phase conditions were used to optimize chromatographic separations of the lipid prodrugs. Mobile phase A consisted of 40% ACN and 1 mM ammonium acetate in ultrapure H_2_O while mobile phase B consisted of 10% ACN 1 mM ammonium acetate in *i*PrOH. The gradient started at 95% mobile phase A and 5% mobile phase B and held for 1 min before transitioning to 5% mobile phase A and 95% mobile phase B over 6 min. Finally, the gradient returned to the starting conditions, which were held for 2 min prior to needle washing. The column temperature was maintained at 40 °C, sample temperature 4 °C, injection volume 8 μL, and the run time 9 min each. Each injection was followed by a strong (1:1:1:1 ACN/H_2_O/*i*PrOH/MeOH) and weak (75:20:5 ACN/H_2_O/*i*PrOH) needle wash. The source was operated using electrospray ionization (ESI) in positive ionization mode. Data was normalized against internal standard (ITSD) AUC. Two technical replicates were carried out for each sample analysis, with 2× intermediate blank washes in between to avoid carryover.

#### Plasma Membrane Fractionation

Total membrane fractionation was performed using a Minute Plasma Membrane Protein Isolation and Cell Fractionation Kit from Invent Biotechnologies (cat No. SM-005). Per the kit protocol, cells were trypsinized, washed with PBS, and lysed in assay buffer with brief vortexing. The lysate was passed through a filter cartridge to pellet nuclei, and the supernatant was centrifuged to separate the cytosol and membrane fractions. The supernatant, containing the cytosol fraction, was removed, and the pellet, containing the total membrane protein fraction, was resuspended in 200 μL ACN and centrifuged again at 20,000 RCF for 30 min to extract the drug compound. The clear supernatant was collected and analyzed using LC-MS/MS.

#### Metabolic Stability Assays

Assays employed human liver microsomes (HLMs, 20 mg/mL) from Xenotech. A 10 mM NADPH stock was prepared in deionized H_2_O, and test compounds were dissolved in 100% MeOH to a final concentration of 10 mM. The stock solutions were diluted to 500 μM in 100 mM potassium phosphate buffer (pH 7.4), keeping the final MeOH concentration below 0.2%. Reaction mixtures were prepared by combining 928 μL phosphate buffer, 55 μL liver microsomes, and the test compound solution to a final concentration of 3 μM. Verapamil was used as a positive control. The reactions were initiated with the addition of 110 μL of 10 mM NADPH. Aliquots were taken in duplicate at 0, 5, 10, 15, and 30 min, quenched with 100 μL of cold MeOH containing 2 μM internal standard (*d*_5_-7-ethoxy coumarin), and centrifuged at 12,000 RCF for 5 min. The supernatants were then transferred to LC-MS vials for analysis. The average of each duplicate was normalized to the 0 min time point, representing 100% remaining (0% metabolism).

#### Cellular Toxicity and Antiviral Activity Assays

The TZM-bl cell line (BEI Resources, ARP-8129) was used to evaluate both cell viability and HIV pseudovirus infectivity reduction. Cytotoxicity in this cell line was measured via two methods. The first method, an ATP luminescence assay, was used for compounds **15a**, **15b**, **21a**, and **21b** as such: 6000 TZM-bl cells per well were seeded in a 96-well plate and incubated at 37 °C with 5% CO_2_ for 24 h. Compounds were prepared as serial dilutions (final concentrations of 1, 0.2, 0.04, 0.008, and 0.0016 μM) in complete DMEM with DEAE-Dextran at 40 μg/mL to mimic HIV infectivity conditions. Solutions were added to the cells and incubated for another 48 h. Viability was assessed using the Promega Cell Titer Glow (CTG) Assay (cat No. G9242) by adding a 50% PBS-CTG solution to each well and measuring luminescence, which correlates with ATP levels, on an Agilent BioTek Synergy HT reader. Cytotoxicity of compounds **8a**–**d** was determined using a colorimetric assay based the conversion of MTT (3-(4,5-dimethylthiazol-2-yl)-2,5-diphenyltetrazolium bromide) to formazan as described previously.^63^ TZM-bl cells were plated into 96-well tissue culture plates 1 day prior to performing the MTT assay. A series of 10 3-fold dilutions of each compound was performed from a starting concentration of 10 mM and each dilution was tested for a reduction in cell viability according to the manufacturer’s instructions (CyQuant MTT Cell Viability Assay, ThermoFisher, V13154). Wells containing TZM-bl cells with media only were used as a control. Briefly, serial dilutions of each test compound were added to the plated TZM-bl cells. At 48 h, the media was removed and replaced with 100 mL of fresh culture medium. 10 mL of 12 mM MTT solution was added to each well and incubated at 37 °C for 4 h. 85 mL of supernatant was then removed from each well and replaced with 50 mL of DMSO. Following a 10 min incubation at 37 °C, the absorbance of each well was determined at 540 nm using a Biotek Synergy Neo2 multimode reader and Gen5 v3.02 software. For infectivity assays, compounds were tested in the following final concentrations: 1, 0.2, 0.04, 0.008, and 0.0016 μM in complete DMEM and combined with 1000 IU of HIV pseudovirus per well. The drug-pseudovirus solution was incubated for 1 h at 37 °C with 5% CO_2_, then added to TZM-bl cells, which were seeded in a 96 well plate 1 day prior as above. After a 48 h incubation, supernatant was aspirated and cells were lysed then freeze–thawed twice at −80 °C. Luciferase activity driven by the HIV LTR was measured using the Promega Luciferase Assay System (cat No. E1501) as previously described.^50,63–65^ Each cytotoxicity and infectivity assay were performed at least twice with duplicate wells, and data are reported as the average of independent replicates.

#### IC_50_ and CC_50_ Calculations

Raw data output from MTT and ATP assays, reflecting cell viability at various concentrations, were imported into GraphPad Prism Version 10.3.1. Compound concentrations were log-transformed and concentration–response curves were generated, followed by nonlinear regression using the four-parameter logistic equation to derive IC_50_ and CC_50_ values.

#### Animal Care During In Vivo Experiments

In accordance with the American Chemical Society Guidelines to Publication of Chemical Research, we affirm the use of standard ethical practices when housing and handling research animals, as ensured by the Emory University’s Institutional Animal Care and Use Committee (IACUC) and the Division of Animal Resources (DAR) through a rigorous protocol review, amendment, and approval process. During *in vivo* experiments, mice were housed in compliance with Emory University’s Division of Animal Resources (DAR) guidelines. For fasting experiments, food was removed the evening before the experiment. Mice had access to water throughout the fasting period. Food was reintroduced 4 h after oral dosing per industry standard protocols. Animals were monitored daily for signs of pain, distress, or adverse events. All procedures were conducted following protocols approved by the Institutional Animal Care and Use Committee (IACUC).

#### Compound Formulation

The compounds **TXL**, **15a**, **15b**, **21a**, **21b**, and a vehicle control were prepared at a concentration of 1 mg/mL in a 90:10 solution of olive oil (Sigma-Aldrich, cat. No. O1514) and 190-proof ethanol. The dosing for each mouse was calculated based on individual body weights to achieve a final dose of 10 mg/kg. For example, a 20 g mouse would receive 200 μL of the 1 mg/mL drug formulation, while a 25 g mouse would receive 250 μL.

#### Animal Handling and Dosing

In accordance with the American Chemical Society Guidelines to Publication of Chemical Research, we affirm the use of standard ethical practices when housing and handling research animals, as ensured by the Emory University’s Institutional Animal Care and Use Committee (IACUC) and the Division of Animal Resources (DAR) through a rigorous protocol review, amendment, and approval process. A total of 220 C57BL6/J male mice (aged 11–12 weeks, weight, 22–30 g) were used for these studies. Each mouse was weighed, recorded, and ear-clipped for identification. The dosing for each mouse was calculated based on individual body weights to achieve a final dose of 10 mg/kg. Animals were anesthetized using isoflurane gas before receiving a single dose of drug prepared as described above using a 22-gauge gavage needle attached to a calibrated syringe.

#### Blood and Tissue Collection

Time points for tissue collection were 1, 2, 4, 8, and 18 h for unfasted mice, and 1, 2, 4, 8, and 24 h for fasted mice. At each time point, the mice (*n* = 4 per compound per time point) were anesthetized using isoflurane and subjected to cardiac puncture for blood collection into EDTA-coated tubes. The collected blood samples were immediately placed on ice before the mice were humanely euthanized via cervical dislocation. Brain, liver, lungs, and kidneys were harvested, rinsed in PBS, and placed in prelabeled tubes. Tissues were flash-frozen in liquid nitrogen then transferred to dry ice for storage.

#### Blood Processing and Sample Storage

Blood samples were collected into EDTA-coated tubes and centrifuged at 400 RCF for 10 min at 4 °C to separate plasma from the cellular components. The plasma layer was carefully pipetted off and stored in separate prelabeled and preweighed tubes. All tissues were stored at −80 °C until time for processing.

#### Tissue Processing, Drug Extraction, and LC-MS/MS Analysis

Tissues were weighed (∼50 mg) into homogenization tubes and ACN containing a 10 μM ITSD was added to achieve a final solvent-to-tissue ratio of 4:1. Homogenization beads were added, and the tissues were processed (Biotage Lysera, part no. 19–060) using two 20 s pulses with a 10 s interval period. Homogenate was centrifuged at maximum speed for 1 h, supernatant collected and analyzed via LC-MS using the method described previously. For plasma samples, a 50 μL aliquot was added 200 μL of ACN containing ITSD. The mixture was vortexed thoroughly and then centrifuged to precipitate proteins, with the supernatant collected and analyzed via LC-MS.

#### Lipophilicity Calculations

*c* Log *D* values were calculated in Maestro of the Schrödinger Small Molecule Drug Discovery software suite using LigPrep and QikProp modules. First, LigPrep was used to add hydrogen atoms, select the appropriate chiral centers, and determine 3D low-energy conformations and protonation states at pH 7.4, 6.5, and 5.5. The resulting structures were evaluated using QikProp, which calculated *c* Log *D*, among other physicochemical properties. *c* Log *D* values were consistent for each compound across the pH 7.4, 6.5, and 5.5, and therefore, only *c* Log *D* pH 6.5 (which is perhaps most representative of the intestinal environment) is discussed herein.

#### Calculating PK Parameters

PK parameters, including half-life (*t*_1/2_) and area under the curve (AUC), were calculated using Phoenix Certara WinNonLin software. Concentration–time data were imported into the software, and noncompartmental analysis was performed to determine AUCs and terminal half-lives using a linear up/log-down calculation with extravascular dosing (10 mg/kg) parameters.

#### Statistical Analysis

All statistical analyses were performed using GraphPad Prism 10. Outliers were identified and removed using Grubbs’ test at a significance level of *p* < 0.05 prior to further analysis. Data were then analyzed using one-way analysis of variance (ANOVA) to determine statistical differences between experimental groups. Post hoc comparisons were made using Tukey’s multiple comparison test to account for comparisons between multiple groups. Statistical significance was determined at a threshold of *p* < 0.05. Data are presented as mean ± standard deviation (SD) unless otherwise noted, and significance levels were indicated on the graphs where appropriate.

### Synthetic Procedures and Compound Characterization

#### General Synthesis and Characterization

Automated flash column chromatography was carried out using a Teledyne ISCO CombiFlash Companion system equipped with RediSep Rf columns, either normal-phase silica gel or reverse-phase C18 gold. Thin-layer chromatography (TLC) involved aluminum-backed silica gel plates (Sigma-Aldrich, F-254) visualized under UV light (254 nm) or stained with phosphomolybdic acid in ethanol. Nuclear magnetic resonance (^1^H, ^13^C, ^19^F, ^31^P) was recorded on Varian INOVA spectrometers (600, 500, or 400 MHz) or VNMR 400 MHz at the Emory University NMR Center. Deuterated solvents CDCl_3_ or CD_3_OD were used, and MestReNova software analyzed chemical shifts in ppm, multiplicities, and coupling constants (Hz). High-resolution mass spectrometry (HRMS) was performed at the Emory Mass Spectrometry Center, while LC-MS used an Agilent 1200 HPLC system with a 6120 Quadrupole mass spectrometer (ESI), employing mixtures of HPLC-grade ACN/H_2_O with 0.1% formic acid through ZORBAX Eclipse XDB-C18 or Poroshell 120 EC-C8 columns. MeOH-dissolved samples were analyzed for purity, with all final compounds achieving ≥95% as determined using NMR and HPLC-MS.

#### General Alkylation Procedure A

To a round-bottom flask with a stir bar was added 1-bromoalkane (3.0 equiv or 1.0 equiv) and a 1:1 mixture (0.2 M) of THF and 50% aqueous NaOH solution. The mixture was stirred vigorously and heated to 75 °C before TBAB (0.2 equiv) and either (*S*)-(+) or (*R*)-(−)-2,2-dimethyl-1,3-dioxolane-4-methanol (1.0 equiv) were added. The resulting reaction mixture was stirred vigorously at 75 °C for 20–24 h, followed by cooling to rt and extracting 3 times with Et_2_O. The combined organic layers were then washed with H_2_O (100 mL) and saturated aqueous NaCl (200 mL) before drying over anhydrous sodium sulfate, filtering, and concentrating *in vacuo* to afford a colorless oil. All ^1^H NMR spectra for **2a**–**d** are consistent with those previously reported.^66^

##### (4*S*)-4-(Hexadecoxymethyl)-2,2-dimethyl-1,3-dioxolane (**2a**)

Synthesis was carried out according to General Alkylation Procedure A section starting from 1-bromohexadecane (5.50 mL, 22.7 mmol, 3.0 equiv) and (*S*)-(+)-2,2-dimethyl-1,3-dioxolane-4-methanol (1.00 mL, 7.57 mmol, 1.0 equiv), and the resulting crude material was purified via column chromatography eluting along a gradient of 0–20% EtOAc in hexanes to yield a white solid (2.08 g, 5.83 mmol, 77% yield). ^1^H NMR (400 MHz, CDCl_3_) δ 4.29 (app p, *J* = 6.0 Hz, 1H), 4.08 (dd, *J* = 8.2, 6.4 Hz, 1H), 3.75 (dd, *J* = 8.3, 6.4 Hz, 1H), 3.58–3.41 (m, 4H), 1.64–1.53 (m, 4H), 1.45 (s, 3H), 1.39 (s, 3H), 1.36–1.25 (m, 24H), 0.88 (t, *J* = 6.5 Hz, 3H).

##### (4*S*)-4-(Octadecoxymethyl)-2,2-dimethyl-1,3-dioxolane (**2b**)

Synthesis was carried out according to General Alkylation Procedure A section starting from 1-bromooctadecane (15.5 mL, 45.3 mmol, 3.0 equiv) and (*S*)-(+)-2,2-dimethyl-1,3-dioxolane-4-methanol (2.00 mL, 15.1 mmol, 1.0 equiv), and the resulting crude material was purified via column chromatography eluting along a gradient of 0–20% EtOAc in hexanes to yield a white solid (5.12 g, 13.3 mmol, 88% yield). ^1^H NMR (400 MHz, CDCl_3_) δ 4.29 (app p, *J* = 6.1 Hz, 1H), 4.08 (dd, *J* = 8.2, 6.4 Hz, 1H), 3.75 (dd, *J* = 8.3, 6.4 Hz, 1H), 3.58–3.39 (m, 4H), 1.65–1.53 (m, 4H), 1.45 (s, 3H), 1.39 (s, 3H), 1.33–1.23 (m, 28H), 0.89 (t, *J* = 6.9 Hz, 3H).

##### (4*R*)-4-(Hexadecoxymethyl)-2,2-dimethyl-1,3-dioxolane (**2c**)

Synthesis was carried out according to General Alkylation Procedure A section starting from 1-bromohexadecane (6.94 mL, 22.7 mmol, 3.0 equiv) and (*R*)-(−)-2,2-dimethyl-1,3-dioxolane-4-methanol (1.00 mL, 7.57 mmol, 1.0 equiv), and the resulting crude material was purified via column chromatography eluting along a gradient of 0–20% EtOAc in hexanes to yield a white solid (2.27 g, 6.36 mmol, 84% yield). ^1^H NMR (400 MHz, CDCl_3_) δ 4.29 (app p, *J* = 6.4 Hz, 1H), 4.08 (dd, *J* = 8.3, 6.4 Hz, 1H), 3.75 (dd, *J* = 8.2, 6.4 Hz, 1H), 3.58–3.40 (m, 4H), 1.65–1.52 (m, 4H), 1.45 (s, 3H), 1.39 (s, 3H), 1.36–1.24 (m, 24H), 0.90 (t, *J* = 6.6 Hz, 3H).

##### (4*R*)-4-(Octadecoxymethyl)-2,2-dimethyl-1,3-dioxolane (**2d**)

Synthesis was carried out according to General Alkylation Procedure A section starting from 1-bromooctadecane (11.6 mL, 34.1 mmol, 3.0 equiv) and (*R*)-(−)-2,2-dimethyl-1,3-dioxolane-4-methanol (1.50 mL, 11.4 mmol, 1.0 equiv) and the resulting crude material was purified via column chromatography eluting along a gradient of 0–20% EtOAc in hexanes to yield a white solid (3.23 g, 8.40 mmol, 74% yield). ^1^H NMR (400 MHz, CDCl_3_) δ 4.29 (app p, *J* = 6.4 Hz, 1H), 4.08 (dd, *J* = 8.2, 6.4 Hz, 1H), 3.75 (dd, *J* = 8.2, 6.4 Hz, 1H), 3.58–3.37 (m, 4H), 1.66–1.52 (m, 4H), 1.45 (s, 3H), 1.39 (s, 3H), 1.36–1.24 (m, 28H), 0.88 (t, *J* = 6.7 Hz, 3H).

#### General Acetonide Deprotection Procedure

Intermediate **2a**–**d** (1.0 equiv) was added to a round-bottom flask with a stir bar, diluted with 80% v/v glacial AcOH in H_2_O (0.2 M), and the resulting reaction mixture was stirred vigorously at 60 °C for 24–72 h. Upon cooling to rt, the mixture was diluted with toluene (100 mL) and concentrated under reduced pressure to yield a clear oil. All ^1^H NMR spectra for **3a**–**d** are consistent with those previously reported.^66^

##### (2*S*)-3-Hexadecoxypropane-1,2-diol (**3a**)

Synthesis was carried out according to the General Acetonide Deprotection Procedure section starting from **2a** (2.10 g, 5.89 mmol, 1.0 equiv), and the resulting crude material was purified via column chromatography eluting along a gradient of 0–100% EtOAc in hexanes to yield a white solid (1.64 g, 5.19 mmol, 88% yield). ^1^H NMR (400 MHz, CDCl_3_) δ 3.91–3.81 (m, 1H), 3.76–3.60 (m, 2H), 3.58–3.42 (m, 4H), 1.61–1.52 (m, 4H), 1.35–1.21 (m, 24H), 0.88 (t, *J* = 6.6 Hz, 3H).

##### (2*S*)-3-Octadecoxypropane-1,2-diol (**3b**)

Synthesis was carried out according to the General Acetonide Deprotection Procedure section starting from **2b** (5.00 g, 13.0 mmol, 1.0 equiv), and the resulting crude material was purified via column chromatography eluting along a gradient of 0–100% EtOAc in hexanes to yield a white solid (4.03 g, 11.7 mmol, 90% yield). ^1^H NMR (400 MHz, CDCl_3_) δ 4.22–3.99 (m, 1H), 3.79–3.63 (m, 2H), 3.60–3.40 (m, 4H), 1.64–1.50 (m, 4H), 1.39–1.13 (m, 28H), 0.90 (t, *J* = 6.3 Hz, 3H).

##### (2*R*)-3-Hexadecoxypropane-1,2-diol (**3c**)

Synthesis was carried out according to the General Acetonide Deprotection Procedure section starting from **2c** (1.20 g, 3.37 mmol, 1.0 equiv), and the resulting crude material was purified via column chromatography eluting along a gradient of 0–100% EtOAc in hexanes to yield a white solid (0.842 g, 2.66 mmol, 79% yield). ^1^H NMR (400 MHz, CDCl_3_) δ 4.24–4.07 (m, 1H), 3.77–3.60 (m, 2H), 3.57–3.37 (m, 4H), 1.67–1.48 (m, 4H), 1.25 (m, 24H), 0.87 (t, *J* = 6.5 Hz, 3H).

##### (2*R*)-3-Octadecoxypropane-1,2-diol (**3d**)

Synthesis was carried out according to the General Acetonide Deprotection Procedure section starting from **2d** (2.80 g, 7.29 mmol, 1.0 equiv), and the resulting crude material was purified via column chromatography eluting along a gradient of 0–100% EtOAc in hexanes to yield a white solid (2.11 g, 6.13 mmol, 84% yield). ^1^H NMR (400 MHz, CDCl_3_) δ 4.23–4.09 (m, 2H), 4.07–3.99 (m, 1H), 3.55–3.35 (m, 4H), 1.65–1.53 (m, 4H), 1.39–1.23 (m, 28H), 0.90 (t, *J* = 6.7 Hz, 3H).

#### General Silylation Procedure

Compound **3a**–**d** or **17a**–**b** (1.0 equiv) was added to a round-bottom flask with a stir bar. The solid was diluted with DCM (0.2 M), Et_3_N (1.4 equiv) and TBSCl (1.4 equiv) were added, and the resulting mixture was stirred vigorously at rt under argon. After a few min of stirring, DMAP (0.06 equiv) was added, and the resulting reaction mixture was stirred vigorously at rt under argon. After 19–24 h, the reaction mixture was partitioned between DCM and H_2_O. The resulting aqueous layer was extracted twice with DCM, and combined organic layers were washed twice with H_2_O, washed once with brine, dried over anhydrous sodium sulfate, filtered, and evaporated under reduced pressure to yield a clear oil. ^1^H NMR spectra for **4a**–**d** are consistent with previously reported.^67^

##### (2*S*)-1-[*tert*-Butyl­(dimethyl)­silyl]­oxy-3-hexadecoxy-propan-2-ol (**4a**)

Synthesis was carried out according to the General Silylation Procedure section starting from **3a** (1.60 g, 5.06 mmol, 1.0 equiv), and the resulting crude material was purified via column chromatography eluting along a gradient of 0–20% EtOAc in hexanes to yield a clear oil (1.46 g, 3.39 mmol, 67% yield). ^1^H NMR (400 MHz, CDCl_3_) δ 3.86–3.78 (m, 1H), 3.65–3.52 (m, 2H), 3.51–3.33 (m, 4H), 1.66–1.52 (m, 4H), 1.37–1.24 (m, 24H), 0.92–0.90 (m, 12H), 0.10 (s, 3H), 0.07 (s, 3H).

##### (2*S*)-1-[*tert*-Butyl­(dimethyl)­silyl]­oxy-3-octadecoxy-propan-2-ol (**4b**)

Synthesis was carried out according to the General Silylation Procedure section starting from **3b** (3.50 g, 10.2 mmol, 1.0 equiv), and the resulting crude material was purified via column chromatography eluting along a gradient of 0–20% EtOAc in hexanes to yield a clear oil (3.63 g, 7.93 mmol, 78% yield). ^1^H NMR (400 MHz, CDCl_3_) δ 3.88–3.79 (m, 1H), 3.65–3.50 (m, 2H), 3.47–3.33 (m, 4H), 1.64–1.51 (m, 4H), 1.39–1.21 (m, 28H), 0.94–0.87 (m, 12H), 0.10 (s, 3H), 0.07 (s, 3H).

##### (2*R*)-1-[*tert*-Butyl­(dimethyl)­silyl]­oxy-3-hexadecoxy-propan-2-ol (**4c**)

Synthesis was carried out according to the General Silylation Procedure section starting from **3c** (0.750 g, 2.37 mmol, 1.0 equiv), and the resulting crude material was purified via column chromatography eluting along a gradient of 0–20% EtOAc in hexanes to yield a clear oil (0.755 g, 1.75 mmol, 74% yield). ^1^H NMR (400 MHz, CDCl_3_) δ 3.86–3.80 (m, 1H), 3.71–3.62 (m, 2H), 3.51–3.41 (m, 4H), 1.65–1.53 (m, 4H), 1.39–1.22 (m, 24H), 0.96–0.91 (m, 12H), 0.09 (s, 6H).

##### (2*R*)-1-[*tert*-Butyl­(dimethyl)­silyl]­oxy-3-octadecoxy-propan-2-ol (**4d**)

Synthesis was carried out according to the General Silylation Procedure section starting from **3d** (2.00 g, 5.81 mmol, 1.0 equiv), and the resulting crude material was purified via column chromatography eluting along a gradient of 0–20% EtOAc in hexanes to yield a clear oil (2.18 g, 4.76 mmol, 82% yield). ^1^H NMR (400 MHz, CDCl_3_) δ 3.80 (app p, *J* = 5.4 Hz, 1H), 3.68–3.59 (m, 2H), 3.48–3.40 (m, 4H), 1.65–1.53 (m, 4H), 1.25 (m, 28H), 0.89–0.88 (m, 12H), 0.07 (s, 6H).

#### General Benzylation Procedure

NaH (60% dispersion in mineral oil, 2.5 equiv) was added to an oven-dried flask with a stir bar. Diluted with anhydrous THF (0.4 M), cooled to 0 °C, and stirred vigorously under argon. A solution of BnBr (2 equiv) in anhydrous THF (0.2 M) was added dropwise via syringe pump at a rate of 4 mL/h, and the resulting mixture was stirred vigorously at 0 °C under argon for 45 min. After this time, a solution of **4a**–**d** or **18a**–**b** (1.0 equiv) in anhydrous THF (0.4 M) was added in dropwise fashion, and the resulting reaction mixture was allowed to slowly warm to rt while vigorously stirring under argon. After 20–24 h, the reaction was carefully quenched with H_2_O, and the resulting aqueous layer was extracted 3 times with Et_2_O. The combined organic layers were washed once with brine, dried over anhydrous sodium sulfate, filtered, and evaporated under reduced pressure to yield a clear oil. ^1^H NMR spectra for **5a**–**d** are consistent with previously reported.^68^

##### *tert*-Butyl-dimethyl-[(2*S*)-2-benzyloxy-3-hexadecoxy-propoxy]­silane (**5a**)

Synthesis was carried out according to the General Benzylation Procedure section starting from **4a** (1.20 g, 2.79 mmol, 1.0 equiv), and the resulting crude material was purified via column chromatography eluting along a gradient of 0–20% EtOAc in hexanes to yield a clear oil (0.813 g, 1.56 mmol, 56% yield). ^1^H NMR (400 MHz, CDCl_3_) δ 7.44–7.30 (m, 5H), 4.72 (s, 1H), 4.53 (s, 1H), 3.72–3.69 (m, 1H), 3.67–3.41 (m, 6H), 1.63–1.53 (m, 4H), 1.28 (m, 24H), 1.01–0.83 (m, 12H), 0.09 (s, 6H).

##### *tert*-Butyl-dimethyl-[(2*S*)-2-benzyloxy-3-octadecoxy-propoxy]­silane (**5b**)

Synthesis was carried out according to the General Benzylation Procedure section starting from **4b** (3.50 g, 7.64 mmol, 1.0 equiv), and the resulting crude material was purified via column chromatography eluting along a gradient of 0–20% EtOAc in hexanes to yield a clear oil (2.93 g, 5.34 mmol, 70% yield). ^1^H NMR (400 MHz, CDCl_3_) δ 7.44–7.32 (m, 5H), 4.72 (s, 1H), 4.53 (s, 1H), 3.73–3.69 (m, 1H), 3.68–3.42 (m, 6H), 1.70–1.53 (m, 4H), 1.29 (m, 28H), 0.92–0.87 (m, 12H), 0.08 (s, 6H).

##### *tert*-Butyl-dimethyl-[(2*R*)-2-benzyloxy-3-hexadecoxy-propoxy]­silane (**5c**)

Synthesis was carried out according to the General Benzylation Procedure section starting from **4c** (0.88 g, 2.10 mmol, 1.0 equiv), and the resulting crude material was purified via column chromatography eluting along a gradient of 0–20% EtOAc in hexanes to yield a clear oil (0.692 g, 1.33 mmol, 65% yield). ^1^H NMR (400 MHz, CDCl_3_) δ 7.44–7.30 (m, 5H), 4.73 (s, 1H), 4.57 (s, 1H), 3.76–3.72 (m, 1H), 3.68–3.42 (m, 6H), 1.63–1.52 (m, 4H), 1.28 (m, 24H), 0.96–0.87 (m, 12H), 0.09 (s, 6H).

##### *tert*-Butyl-dimethyl-[(2*R*)-2-benzyloxy-3-octadecoxy-propoxy]­silane (**5d**)

Synthesis was carried out according to the General Benzylation Procedure section starting from **4d** (2.30 g, 5.02 mmol, 1.0 equiv), and the resulting crude material was purified via column chromatography eluting along a gradient of 0–20% EtOAc in hexanes to yield a clear oil (1.98 g, 3.61 mmol, 72% yield). ^1^H NMR (400 MHz, CDCl_3_) δ 7.41–7.29 (m, 5H), 4.72 (s, 1H), 4.57 (s, 1H), 3.76–3.69 (m, 1H), 3.68–3.38 (m, 6H), 1.72–1.48 (m, 4H), 1.40–1.21 (m, 28H), 0.95–0.90 (m, 12H), 0.09 (s, 6H).

#### General Silyl Deprotection Procedure

Anhydrous pyridine was first added to a polypropylene tube equipped with a stir bar. Hydrofluoric acid (70%) in pyridine was then added and the resulting solution was stirred vigorously at rt. A solution of **5a**–**d** or **19a**–**b** in anhydrous THF (0.18 M) was added, and the resulting reaction mixture was stirred vigorously at rt. After 20–24 h, TLC indicated complete consumption of starting material. Accordingly, the reaction mixture was partitioned between EtOAc and brine, and the resulting aqueous layer was extracted twice with EtOAc. The combined organic layers were washed once with brine, dried over anhydrous sodium sulfate, filtered, and evaporated under reduced pressure to yield a clear oil. ^1^H NMR spectra for **6a**–**d** are consistent with previously reported.^69^

##### (2*S*)-2-Benzyloxy-3-hexadecoxy-propan-1-ol (**6a**)

Synthesis was carried out according to the General Silyl Deprotection Procedure section starting from **5a** (0.561 g, 1.10 mmol, 1.0 equiv), and the resulting crude material was purified via column chromatography eluting along a gradient of 0–50% EtOAc in hexanes to yield a clear oil (0.328 g, 0.810 mmol, 75% yield). ^1^H NMR (400 MHz, CDCl_3_) δ 7.40–7.29 (m, 5H), 4.70 (dd, *J* = 8.3, 6.2 Hz, 2H), 3.82–3.75 (m, 1H), 3.72–3.46 (m, 6H), 1.59–1.49 (m, 4H), 1.39–1.22 (m, 24H), 0.90 (t, *J* = 6.6 Hz, 3H).

##### (2*S*)-2-Benzyloxy-3-octadecoxy-propan-1-ol (**6b**)

Synthesis was carried out according to the General Silyl Deprotection Procedure section starting from **5b** (1.33 g, 2.43 mmol, 1.0 equiv), and the resulting crude material was purified via column chromatography eluting along a gradient of 0–50% EtOAc in hexanes to yield a clear oil (0.841 g, 1.90 mmol, 83% yield). ^1^H NMR (400 MHz, CDCl_3_) δ 7.38–7.27 (m, 5H), 4.67 (dd, *J* = 8.1, 6.2 Hz 2H), 3.71–3.64 (m, 1H), 3.62–3.37 (m, 6H), 1.63–1.53 (m, 4H), 1.36–1.19 (m, 28H), 0.90 (t, *J* = 6.8 Hz, 3H).

##### (2*R*)-2-Benzyloxy-3-hexadecoxy-propan-1-ol (**6c**)

Synthesis was carried out according to the General Silyl Deprotection Procedure section starting from **5c** (0.344 g, 0.651 mmol, 1.0 equiv), and the resulting crude material was purified via column chromatography eluting along a gradient of 0–50% EtOAc in hexanes to yield a clear oil (0.211 g, 0.523 mmol, 80% yield). ^1^H NMR (600 MHz, CDCl_3_) δ 7.35–7.29 (m, 5H), 4.73 (dd, *J* = 8.2, 6.4 Hz 2H), 3.77–3.71 (m, 1H), 3.67–3.36 (m, 6H), 1.55–1.34 (m, 4H), 1.34–1.20 (m, 24H), 0.86 (t, J = 7.0 Hz, 3H).

##### (2*R*)-2-Benzyloxy-3-octadecoxy-propan-1-ol (**6d**)

Synthesis was carried out according to the General Silyl Deprotection Procedure section starting from **5d** (0.500 g, 0.902 mmol, 1.0 equiv), and the resulting crude material was purified via column chromatography eluting along a gradient of 0–50% EtOAc in hexanes to yield a clear oil (0.255 g, 0.587 mmol, 65% yield). ^1^H NMR (600 MHz, CDCl_3_) δ 7.32–7.23 (m, 5H), 4.58 (dd, *J* = 7.7, 5.8 Hz, 2H), 3.71–3.64 (m, 1H), 3.61–3.22 (m, 6H), 1.51–1.34 (m, 4H), 1.34–1.12 (m, 28H), 0.79 (t, *J* = 7.1 Hz, 3H).

#### General TFV Coupling Procedure

To a Schlenk tube containing a stir bar was added intermediate **6**, **14** or **20** (1.0 equiv or 1.2 equiv), **TFV** (**7**, 1.0 equiv), DCC (1.6 equiv) under argon. Diluted with anhydrous NMP (0.2 M), added DMAP (0.02 equiv), and the resulting turbid mixture was then heated to 100 °C with vigorous stirring. After 5 min, the solution became clear and colorless, and after 20–24 h, the reaction mixture was dark yellow. After this time, the reaction mixture was cooled to rt, during which a white precipitate formed. A few drops of H_2_O were added to facilitate additional precipitation before the mixture was filtered and concentrated *in vacuo*. Standard 3-step purification involved (1) normal phase flash chromatography (0–100% 80:20:3 DCM/MeOH/NH_4_OH in DCM), (2) reverse phase (C18) column chromatography (10–100% MeOH in H_2_O), and (3) stirring for 15 min in 7 N methanolic ammonia solution (to form the ammonium salt) before drying under vacuum to yield a fluffy white solid.

##### Ammonium­[(1*R*)-2-(6-aminopurin-9-yl)-1-methyl-ethoxy]­methyl-[(2*S*)-2-benzyloxy-3-hexadecoxy-propoxy]­phosphinate (**8a**)

Synthesis was carried out according to the General TFV Coupling Procedure section starting from intermediate **6a** (0.250 g, 0.621 mmol, 1.0 equiv) and TFV **7** (0.182 g, 0.621 mmol, 1.0 equiv) to yield a white solid (0.174 g, 0.258 mmol, 42% yield). ^1^H NMR (400 MHz, CD_3_OD) δ 8.31 (s, 1H), 8.19 (s, 1H), 7.42–7.17 (m, 5H), 4.62 (s, 2H), 4.33 (dd, *J* = 14.4, 3.2 Hz, 1H), 4.18 (dd, *J* = 14.4, 6.4 Hz, 1H), 3.94 (t, *J* = 5.7 Hz, 2H), 3.89–3.79 (m, 1H), 3.77–3.66 (m, 2H), 3.60–3.45 (m, 3H), 3.40 (td, *J* = 6.5, 1.1 Hz, 2H), 1.57–1.46 (m, 2H), 1.38–1.24 (m, 26H), 1.11 (d, *J* = 6.2 Hz, 3H), 0.89 (t, *J* = 6.6 Hz, 3H). ^13^C NMR (151 MHz, CD_3_OD) δ 157.0, 153.3, 150.9, 144.3, 140.1, 129.2 (2C), 128.8 (2C), 128.4, 119.5, 79.1, 76.9 (d, *J*_CP_ = 13.0 Hz), 73.1, 72.6, 71.9, 65.6, 65.5, 65.5 (d, *J*_CP_ = 5.6 Hz), 33.1, 30.8 (8C), 30.7, 30.6, 30.5, 27.3, 23.7, 16.9, 14.5. ^31^P NMR (162 MHz, CD_3_OD) δ 16.3. HRMS (ESI) [M + H]^+^ calc. for C_35_H_59_O_6_N_5_P, 676.41975, observed, 676.41983. LC-MS (ESI, C8, 0.5 mL/min) 75–95% ACN in H_2_O, 6 min, rt = 1.210, *m*/*z* = 676.5 [M + H]^+^ and 674.5 [M – H]^−^.

##### Ammonium­[(1*R*)-2-(6-aminopurin-9-yl)-1-methyl-ethoxy]­methyl-[(2*S*)-2-benzyloxy-3-octadecoxy-propoxy]­phosphinate (**8b**)

Synthesis was carried out according to the General TFV Coupling Procedure section starting from intermediate **6b** (0.850 g, 1.96 mmol, 1.0 equiv) and TFV **7** (0.563 g, 1.96 mmol, 1.0 equiv) to yield a white solid (0.370 g, 0.582 mmol, 27% yield). ^1^H NMR (400 MHz, CD_3_OD) δ 8.32 (s, 1H), 8.19 (s, 1H), 7.35–7.16 (m, 5H), 4.62 (s, 2H), 4.33 (dd, *J* = 14.4, 3.2 Hz, 1H), 4.18 (dd, *J* = 14.4, 6.3 Hz, 1H), 3.94 (t, *J* = 6.5 Hz, 2H), 3.77–3.66 (m, 3H), 3.59–3.44 (m, 3H), 3.40 (td, *J* = 6.5, 1.2 Hz, 2H), 1.57–1.46 (m, 2H), 1.27 (s, 30H), 1.11 (d, *J* = 6.2 Hz, 3H), 0.89 (t, *J* = 6.5, 3H). ^13^C CD_3_OD δ 157.0, 153.2, 150.9, 144.3, 140.2, 129.2 (2C), 128.8 (2C), 128.4, 119.5, 79.1, 76.9 (d, *J*_CP_ = 12.8 Hz), 73.1, 72.6, 71.9, 65.6, 65.5, 65.5 (d, *J* = 6.9 Hz), 33.1, 30.8 (10C), 30.7, 30.6, 30.5, 27.3, 23.7, 16.9, 14.5. ^31^P NMR (162 MHz, CD_3_OD) δ 17.1. HRMS (ESI) [M + H]^+^ calc. for C_37_H_63_O_6_N_5_P, 704.45105, observed, 704.45105. LC-MS (ESI, C8, 0.5 mL/min) 50–95% ACN in H_2_O, 6 min, rt = 5.447, *m*/*z* = 704.4 [M + H]^+^ and 702.2 [M – H]^−^; 75–95% ACN in H_2_O, 6 min, rt = 2.210, *m*/*z* = 704.4 [M + H]^+^ and 702.2 [M – H]^−^.

##### Ammonium­[(1*R*)-2-(6-aminopurin-9-yl)-1-methyl-ethoxy]­methyl-[(2*R*)-2-benzyloxy-3-hexadecoxy-propoxy]­phosphinate (**8c**)

Synthesis was carried out according to the General TFV Coupling Procedure section starting from intermediate **6c** (0.172 g, 0.433 mmol, 1.0 equiv) and TFV **7** (0.124 g, 0.433 mmol, 1.0 equiv) to yield a white solid (0.095 g, 0.144 mmol, 33% yield). ^1^H NMR (400 MHz, CD_3_OD) δ 8.39 (s, 1H), 8.26 (s, 1H), 7.41–7.24 (m, 5H), 4.69 (s, 2H), 4.40 (dd, *J* = 14.4, 3.2 Hz, 1H), 4.25 (dd, *J* = 14.4, 6.3 Hz, 1H), 4.02 (t, *J* = 6.3 Hz, 2H), 3.97–3.89 (m, 1H), 3.84–3.75 (m, 2H), 3.65–3.54 (m, 3H), 3.47 (td, *J* = 6.5, 1.1 Hz, 2H), 1.63–1.54 (m, 2H), 1.43–1.28 (m, 26H), 1.18 (d, *J* = 6.2 Hz, 3H), 0.93 (t, *J* = 6.4 Hz, 3H). ^13^C NMR (101 MHz, CD_3_OD) δ 155.5, 151.7, 149.6, 143.0, 138.8, 127.8 (2C), 127.5 (2C), 127.0, 118.2, 77.7, 75.5 (d, *J*_CP_ = 12.6 Hz), 71.7, 71.2, 70.5, 64.4, 64.3, 64.2 (d, = 5.9 Hz), 31.7, 29.4 (m, 9C), 29.2, 29.0, 25.9, 22.3, 15.5, 13.0. ^31^P NMR (162 MHz, CD_3_OD) δ 17.0. HRMS (ESI) [M + H]^+^ calc. for C_35_H_59_O_6_N_5_P, 676.41841, observed, 676.41873. LC-MS (ESI, C8, 0.5 mL/min) 75–95% ACN in H_2_O, 6 min, rt = 1.268, *m*/*z* = 676.5 [M + H]^+^ and 674.5 [M – H]^−^; 50–95% ACN in H_2_O, 6 min, rt = 4.032, *m*/*z* = 676.5 [M + H]^+^ and 674.5 [M – H]^−^.

##### Ammonium­[(1*R*)-2-(6-aminopurin-9-yl)-1-methyl-ethoxy]­methyl-[(2*R*)-2-benzyloxy-3-octadecoxy-propoxy]­phosphinate (**8d**)

Synthesis was carried out according to the General TFV Coupling Procedure section starting from intermediate **6d** (0.250 g, 0.572 mmol, 1.0 equiv) and TFV **7** (0.16 g, 0.572 mmol, 1.0 equiv) to yield a white solid (0.161 g, 0.228 mmol, 40% yield). ^1^H NMR (600 MHz, CD_3_OD) δ 8.39 (s, 1H), 8.22 (s, 1H), 7.38–7.17 (m, 5H), 4.63 (br s, 2H), 4.38 (dd, *J* = 14.5, 3.1 Hz, 1H), 4.20 (dd, *J* = 14.5, 6.7 Hz, 1H), 4.01–3.93 (m, 2H), 3.82–3.72 (m, 3H), 3.65 (dd, *J* = 13.1, 9.3 Hz, 1H), 3.61–3.50 (m, 2H), 3.5–3.4 (m, 2H), 1.57–1.50 (m, 2H), 1.38–1.22 (m, 30H), 1.10 (d, *J* = 6.3 Hz, 3H), 0.90 (t, *J* = 7.0 Hz, 3H). ^13^C NMR (101 MHz, CD_3_OD) δ 155.0, 150.9, 149.5, 143.2, 138.8, 127.8 (2C), 127.4 (2C), 127.0, 118.1, 77.8, 75.5 (d, *J* = 12.6 Hz), 71.7, 71.2, 70.5, 64.2, 64.2, 64.1 (d, *J* = 6.2), 31.7, 29.6 (11C), 29.2, 29.1, 25.9, 22.3, 15.5, 13.0. ^31^P NMR (162 MHz, CD_3_OD) δ 16.3. HRMS (ESI) [M + H]^+^ calc. for C_37_H_63_O_6_N_5_P, 704.45105, observed, 704.45111. LC-MS (ESI, C8, 0.5 mL/min) 85–95% ACN in H_2_O, 6 min, rt = 2.102, *m*/*z* = 704.4 [M + H]^+^ and 702.2 [M – H]^−^; 55–95% ACN in H_2_O, 6 min, rt = 4.858, *m*/*z* = 704.4 [M + H]^+^ and 702.2 [M – H]^−^.

#### General PMB Protection Procedure

NaH (60% dispersion in mineral oil, 2.5 equiv) was added to a solution of either undec-10-yn-1-ol or oct-7-yn-1-ol (1.0 equiv) in THF (0.2 M), and the resulting mixture was stirred vigorously at rt. After 30 min, 4-methoxybenzyl chloride (1.5 equiv) was added dropwise, and the resulting reaction was stirred vigorously for 22–24 h at rt. After this time, the reaction mixture was carefully quenched with a saturated aqueous ammonium chloride, and the aqueous layer was extracted three times with EtOAc. The combined organic phases were subsequently washed twice with brine, dried over anhydrous magnesium sulfate, filtered, and concentrated *in vacuo*. All NMR are consistent with previously reported.^70^

##### 1-Methoxy-4-(undec-10-ynoxymethyl)­benzene (**10a**)

Synthesis was carried out according to the General PMB Protection Procedure section starting from undec-10-yn-1-ol (5.00 mL, 39.7 mmol, 1.0 equiv), and the resulting crude material was purified via column chromatography eluting along a gradient of 5–20% EtOAc in hexanes to yield a white solid (6.87 g, 23.0 mmol, 60% yield). ^1^H NMR (400 MHz, CDCl_3_) δ 7.35–7.27 (m, 2H), 6.95–6.89 (m, 2H), 4.46 (s, 2H), 3.83 (s, 3H), 3.47 (t, *J* = 6.6 Hz, 2H), 2.21 (td, *J* = 7.7, 2.7 Hz, 2H), 1.97 (t, *J* = 2.5 Hz, 1H), 1.70–1.51 (m, 6H), 1.47–1.35 (m, 8H). ^13^C NMR (101 MHz, CDCl_3_) δ 159.2, 130.9, 129.3 (2C), 113.8 (2C), 84.7, 72.6, 70.2, 68.3, 55.3, 29.7 (4C), 28.7, 28.5, 25.8, 18.4. HRMS (ESI) [M + H]^+^ calc. for C_19_H_29_O_2_, 289.21621, observed, 289.21596.

##### 1-Methoxy-4-(oct-7-ynoxymethyl)­benzene (**10b**)

Synthesis was carried out according to the General PMB Protection Procedure section starting from oct-7-yn-1-ol (11.0 mL, 87.3 mmol, 1.0 equiv), and the resulting crude material was purified via column chromatography eluting along a gradient of 5–20% EtOAc in hexanes to yield a white solid. (11.2 g, 45.5 mmol, 57% yield). ^1^H NMR (400 MHz, CDCl_3_) δ 7.30–7.23 (m, 2H), 6.92–6.84 (m, 2H), 4.43 (s, 2H), 3.80 (s, 3H), 3.44 (t, *J* = 6.6 Hz, 2H), 2.18 (td, *J* = 7.1, 2.7 Hz, 2H), 1.93 (t, *J* = 2.6 Hz, 1H), 1.67–1.47 (m, 4H), 1.47–1.31 (m, 4H). HRMS (ESI) [M – H]^−^ calc. for C_16_H_21_O_2_, 245.15361, observed, 245.15405.

#### General Alkylation Procedure B

To a stirring solution of **10a**–**b** (1.0 equiv) and DMPU (3.0 equiv) in THF (0.2 M) −78 °C was carefully added *n*-butyllithium (2.0 equiv) dropwise over 20 min under an atmosphere of argon. Upon completion of the addition, the resulting mixture was stirred vigorously at −78 °C for 1 h. After this time, either 1,1,1-trifluoro-5-iodopentane or 1,1,1-trifluoro-10-bromodecane (1.5 equiv or 2.0 equiv) was added dropwise at −78 °C, and the resulting reaction mixture was stirred vigorously while slowly warming to rt overnight. After 18–20 h, the reaction was carefully quenched with H_2_O, and the aqueous layer was extracted three times with DCM. The combined organic phases were subsequently washed twice with brine, dried over anhydrous magnesium sulfate, filtered, and concentrated *in vacuo* to yield a clear oil.

##### 1-Methoxy-4-(16,16,16-trifluorohexadec-10-ynoxymethyl)­benzene (**11a**)

Synthesis was carried out according to General Alkylation Procedure B section starting from **10a** (6.00 g, 20.8 mmol, 1.0 equiv) and reacting with 1,1,1-trifluoro-5-iodopentane (10.5 g 41.6 mmol, 2.0 equiv). The resulting crude material was purified via column chromatography eluting along a gradient of 0–20% EtOAc in hexanes to yield a clear oil (5.32 g, 12.9 mmol, 62% yield). ^1^H NMR (400 MHz, CDCl_3_) δ 7.21–7.16 (m, 2H), 6.82–6.78 (m, 2H), 4.35 (s, 2H), 3.72 (s, 3H), 3.36 (t, *J* = 6.5 Hz, 2H), 2.06 (s, 2H), 1.97 (dd, *J* = 16.2, 11.0 Hz, 2H), 1.57–1.16 (m, 20H). ^13^C NMR (101 MHz, CDCl_3_) δ 159.2, 130.9, 129.3 (2C), 127.4 (q, *J*_CF_ = 275.9 Hz), 113.9 (2C), 80.3, 72.6, 70.2, 69.2, 55.4, 34.0 (q, *J*_CF_ = 27.6 Hz), 29.3 (8C), 25.9, 22.0 (q, *J*_CF_ = 2.8 Hz), 18.9. ^19^F NMR (376 MHz, CDCl_3_) δ −66.4 (t, *J* = 11.4 Hz). HRMS (ESI) [M]^•+^ calc. for C_24_H_35_O_2_F_3_, 412.25983, observed, 412.25935.

##### 1-Methoxy-4-(18,18,18-trifluorooctadec-7-ynoxymethyl)­benzene (**11b**)

Synthesis was carried out according to General Alkylation Procedure B section starting from **10b** (7.00 g, 28.4 mmol, 1.0 equiv) and reacting with 1,1,1-trifluoro-10-bromodecane (11.7 g, 42.6 mmol, 1.5 equiv). The resulting crude material was purified via column chromatography eluting along a gradient of 0–20% EtOAc in hexanes to yield a clear oil (5.14 g, 11.7 mmol, 41% yield). ^1^H NMR (400 MHz, CDCl_3_) δ 7.30–7.22 (m, 2H), 6.92–6.84 (m, 2H), 4.43 (s, 2H), 3.80 (s, 3H), 3.43 (t, *J* = 6.6 Hz, 2H), 2.18–2.10 (m, 6H), 1.63–1.26 (m, 22H). ^13^C NMR (101 MHz, CDCl_3_) δ 159.2, 130.9, 129.4 (2C), 127.4 (q, *J*_CF_ = 276.2 Hz), 113.9 (2C), 80.4, 72.7, 70.3, 69.3, 55.4, 33.9 (q, *J*_CF_ = 28.2 Hz), 29.8, 29.4, 29.3, 29.2 (4C), 28.9, 28.8, 28.8, 25.9, 22.0 (q, *J*_CF_ = 2.9 Hz), 18.9. ^19^F NMR (376 MHz, CDCl_3_) δ −66.4 (t, *J* = 11.0 Hz). HRMS (ESI) [M + H]^+^ calc. for C_26_H_40_O_2_F_3_, 441.29749, observed, 441.29767.

#### General Hydrogenation Procedure

Compound **11a**–**b** (1.0 equiv) was added to an oven-dried flask equipped with a magnetic stir bar, and the starting material was dissolved in EtOAc (0.2 M). The solution was subsequently degassed under light vacuum with vigorous stirring for approximately 10 min, and then the reaction flask was purged with argon. This cycle was repeated twice more before the addition of palladium hydroxide on carbon (20 wt %, 0.1 equiv). Once more, the reaction flask was placed under vacuum with vigorous stirring before a final flush using a balloon of H_2_ gas. The resulting reaction mixture was then stirred vigorously under an atmosphere of H_2_ at rt for 18–22 h. After this time, the heterogeneous reaction mixture was filtered over a bed of Celite, which was washed thoroughly with EtOAC, and the mother liquor was concentrated under reduced pressure to yield a white solid.

##### 16,16,16-Trifluorohexadecan-1-ol (**12a**)

Synthesis was carried out according to the General Hydrogenation Procedure section starting from **11a** (5.00 g, 12.1 mmol, 1.0 equiv), and the resulting crude material was purified via column chromatography eluting along a gradient of 0–20% EtOAc in hexanes to yield a white solid (2.34 g, 7.88 mmol, 65% yield). ^1^H NMR (400 MHz, CDCl_3_) δ 3.66 (t, *J* = 6.7 Hz, 2H), 2.14–2.01 (m, 2H), 1.66–1.52 (m, 4H), 1.38–1.24 (m, 23H). ^13^C NMR (101 MHz, CDCl_3_) δ 127.4 (q, *J*_CF_ = 279.4 Hz), 63.3, 34.0 (q, *J*_CF_ = 27.1 Hz), 33.0, 29.7 (10C), 25.9, 22.0. ^19^F NMR (376 MHz, CDCl_3_) δ −66.4 (t, *J* = 11.0 Hz). ^1^H NMR spectrum is consistent with what was previously reported.^71^

##### 18,18,18-Trifluorooctadecan-1-ol (**12b**)

Synthesis was carried out according to the General Hydrogenation Procedure section starting from **11b** (5.14 g, 11.7 mmol, 1.0 equiv) and the resulting crude material was purified via column chromatography eluting along a gradient of 0–20% EtOAc in hexanes to yield a white solid (3.15 g, 9.71 mmol, 83% yield). ^1^H NMR (400 MHz, CDCl_3_) δ 3.64 (t, *J* = 6.6 Hz, 2H), 2.14–1.97 (m, 2H), 1.60–1.48 (m, 4H), 1.41–1.20 (m, 26H). ^13^C NMR (101 MHz, CDCl_3_) δ 129.1 (q, *J*_CF_ = 276.2 Hz), 63.3, 34.0 (q, *J*_CF_ = 27.7 Hz), 33.0, 29.7 (12C), 25.9, 22.0. ^19^F NMR (376 MHz, CDCl_3_) δ −66.4 (t, *J* = 11.0 Hz). HRMS (ESI) [M + Na]^+^ calc. for C_18_H_35_OF_3_Na 347.2532, observed, 347.2539. ^1^H and ^13^C NMR spectra are consistent with what was previously reported.^72^

#### General Tosylation Procedure

Intermediate **12a**–**b** (1.0 equiv), DMAP (0.01 equiv), Et_3_N (1.2 equiv) and TsCl (1.2 equiv) were stirred vigorously in DCM (0.2 M) at 0 °C under argon atmosphere for 30 min before allowing the mixture to slowly warm to rt. After 16–18 h, the reaction mixture was concentration *in vacuo*.

##### 16,16,16-Trifluorohexadecyl 4-methylbenzenesulfonate (**13a**)

Synthesis was carried out according to the General Tosylation Procedure section starting from **12a** (2.00 g, 6.75 mmol, 1.0 equiv), and the resulting crude material was purified via column chromatography eluting along a gradient of 0–10% EtOAc in hexanes to yield a white solid (2.67 g, 5.94 mmol, 88% yield). ^1^H NMR (400 MHz, CDCl_3_) δ 7.81 (d, *J* = 8.6 Hz, 2H), 7.37 (d, *J* = 8.0 Hz, 2H), 4.03 (t, *J* = 6.5 Hz, 2H), 2.47 (s, 3H), 2.15–2.01 (m, 2H), 1.70–1.58 (m, 4H), 1.43–1.17 (m, 22H). ^13^C NMR (101 MHz, CDCl_3_) δ 144.8, 133.3, 127.4 (q, *J*_CF_ = 276.11 Hz), 129.9 (2C), 128.0 (2C), 70.9, 33.9 (q, *J*_CF_ = 28.2 Hz), 30.1 (11C), 25.5, 22.0 (q, *J*_CF_ = 2.9 Hz), 21.8. ^19^F NMR (376 MHz, CDCl_3_) δ −66.4 (t, *J* = 11.1 Hz). HRMS (ESI) [M + H]^+^ calc. for C_23_H_38_O_3_F_3_S, 451.24883, observed, 451.24819.

##### 18,18,18-Trifluorooctadecyl 4-methylbenzenesulfonate (**13b**)

Synthesis was carried out according to the General Tosylation Procedure section starting from **12b** (0.500 g, 1.53 mmol, 1.0 equiv), and the resulting crude material was purified via column chromatography eluting along a gradient of 0–10% EtOAc in hexanes to yield a white solid (0.554 g, 1.16 mmol, 75% yield). ^1^H NMR (400 MHz, CDCl_3_) δ 7.79 (d, *J* = 8.3 Hz, 2H), 7.34 (d, *J* = 8.0 Hz, 2H), 4.01 (t, *J* = 6.5 Hz, 2H), 2.45 (s, 3H), 2.14–1.97 (m, 2H), 1.68–1.48 (m, 4H), 1.38–1.19 (m, 26H). ^13^C NMR (101 MHz, CDCl_3_) δ 144.8, 133.4, 129.9 (2C), 128.0 (2C), 127.5 (q, *J*_CF_ = 277.12), 70.9, 33.9 (q, *J*_CF_ = 28.3 Hz), 30.0 (13C), 25.5, 22.0 (q, *J*_CF_ = 2.9 Hz), 21.8. ^19^F NMR (376 MHz, CDCl_3_) δ −66.4 (t, *J* = 11.0 Hz). HRMS (ESI) [M + Na]^+^ calc. for C_25_H_41_O_3_F_3_SNa, 501.2621, observed, 501.2632.

##### 3-(16,16,16-Trifluorohexadecoxy)­propan-1-ol (**14a**)

**14a** was synthesized as previously reported.^40^

##### 3-(18,18,18-Trifluorooctadecoxy)­propan-1-ol (**14b**)

In an oven-dried flask under an atmosphere of argon, NaH (60% dispersion in mineral oil, 0.090 g, 2.91 mmol, 2.5 equiv) was added to 1,3-propanediol (0.229 g, 2.32 mmol, 2.0 equiv) in DMF (0.2 M) at 0 °C. After 1 h of vigorous stirring at this temperature, **13b** (0.554 g, 1.16 mmol, 1.0 equiv) was added, and the resulting reaction mixture was allowed to slowly warm to rt overnight. After 18 h, the reaction was carefully quenched with H_2_O, and the aqueous layer was extracted three times with DCM. The organic phases were combined, washed twice with brine, dried over anhydrous magnesium sulfate, filtered and concentrated *in vacuo*. The resulting crude material was then purified via column chromatography eluting along a gradient of 0–20% EtOAc in hexanes to yield a white solid (0.373 g, 0.975 mmol, 84% yield). ^1^H NMR (400 MHz, CDCl_3_) δ 3.80 (app q, *J* = 5.0 Hz, 2H), 3.64 (t, *J* = 5.7 Hz, 2H), 3.45 (t, *J* = 6.6 Hz, 2H), 2.52 (br s, 1H), 2.13–2.01 (m, 2H), 1.85 (p, *J* = 5.6 Hz, 2H), 1.64–1.50 (m, 4H), 1.41–1.25 (m, 26H). ^13^C NMR (101 MHz, CDCl_3_) δ 127.4 (q, *J*_CF_ = 276.2 Hz), 71.7, 70.7, 62.7, 33.9 (q, *J*_CF_ = 28.3 Hz), 32.0, 29.9, 29.8 (4C), 29.8 (2C), 29.7, 29.7, 29.6, 29.5, 29.3, 28.8, 26.3, 22.0 (q, *J*_CF_ = 2.9 Hz). ^19^F NMR (376 MHz, CDCl_3_) δ −66.4 (t, *J* = 11.0 Hz). HRMS (ESI) [M + H]^+^ calc. for C_21_H_42_O_2_F_3_, 383.31314, observed, 383.31359.

##### Ammonium­[(1*R*)-2-(6-aminopurin-9-yl)-1-methyl-ethoxy]­methyl-[3-(16,16,16-trifluorohexadecoxy)­propoxy]­phosphinate (**15a**)

**15a** was synthesized as previously reported.^40^

##### Ammonium­[(1*R*)-2-(6-aminopurin-9-yl)-1-methyl-ethoxy]­methyl-[3-(18,18,18-trifluorooctadecoxy)­propoxy]­phosphinate (**15b**)

Synthesis was carried out according to the General TFV Coupling Procedure section starting from intermediate **14b** (0.320 g, 0.843 mmol, 1.2 equiv) and TFV **7** (0.200 g, 0.701 mmol, 1.0 equiv) to yield **15b** as a white solid (0.229 g, 0.342 mmol, 49% yield). ^1^H NMR (400 MHz, CD_3_OD) δ 8.32 (s, 1H), 8.20 (s, 1H), 4.38 (dd, *J* = 14.4, 3.2 Hz, 1H), 4.23 (dd, *J* = 14.4, 6.6 Hz, 1H), 3.95–3.81 (m, 3H), 3.71 (dd, *J* = 12.8, 9.5 Hz, 1H), 3.52–3.39 (m, 3H), 3.34 (t, *J* = 6.6 Hz, 2H), 2.21–2.04 (m, 2H), 1.77 (m, 2H), 1.52 (m, 4H), 1.27 (d, *J* = 6.6 Hz, 26H), 1.16 (d, *J* = 6.3 Hz, 3H). ^13^C NMR (101 MHz, CD_3_OD) δ 157.1, 153.3, 150.9, 144.3, 128.9 (q, *J*_CF_ = 275.3 Hz), 119.5, 76.9 (d, *J*_CP_ = 13.0 Hz), 72.0, 68.4, 66.2, 64.6, 63.1 (d, *J*_CP_ = 5.7 Hz), 34.4 (q, *J*_CF_ = 28.1 Hz), 32.4, 30.9–30.7 (7C), 30.7, 30.7, 30.6, 30.4, 29.8, 27.3, 23.1, 23.0, 16.8. ^19^F NMR (376 MHz, CD_3_OD) δ −68.8 (t, *J =* 11.2 Hz). ^31^P NMR (162 MHz, CD_3_OD) δ 15.3. HRMS (ESI) [M + H]^+^ calc. for C_30_H_54_O_5_N_5_F_3_P, 652.38092, observed, 652.38064. LC-MS (ESI, C8, 0.5 mL/min) 45–95% ACN in H_2_O, 6 min, rt = 4.023, *m*/*z* = 652.5 [M + H]^+^ and 650.5 [M – H]^−^; 65–95% ACN in H_2_O, 6 min, rt = 1.151, *m*/*z* = 652.5 [M + H]^+^ and 650.5 [M – H]^−^.

##### (4*S*)-2,2-Dimethyl-4-(16,16,16-trifluorohexadecoxymethyl)-1,3-dioxolane (**16a**)

Synthesis was carried out according to General Alkylation Procedure A section starting from intermediate **13a** (2.51 g, 5.56 mmol, 1.0 equiv) and (*S*)-(+)-2,2-dimethyl-1,3-dioxolane-4-methanol (0.734 g, 5.56 mmol, 1.0 equiv), and the resulting crude material was purified via column chromatography eluting along a gradient of 0–20% EtOAc in hexanes to yield a white solid (1.28 g, 3.12 mmol, 56% yield). ^1^H NMR (400 MHz, CDCl_3_) δ 4.26 (p, *J* = 6.0, 1H), 4.06 (dd, *J* = 8.3, 6.4 Hz, 1H), 3.73 (dd, *J* = 8.2, 6.4 Hz, 1H), 3.55–3.36 (m, 4H), 2.13–1.97 (m, 2H), 1.64–1.47 (m, 4H), 1.42 (s, 3H), 1.36 (s, 3H), 1.34–1.20 (m, 22H). ^13^C NMR (101 MHz, CDCl_3_) δ 128.8 (q, *J*_CF_ = 281.6 Hz), 109.5, 74.9, 72.0, 72.0, 67.1, 33.7 (q, *J*_CF_ = 28.2 Hz), 29.8, 29.6, 29.5 (9C), 28.8, 26.9, 26.2, 25.6. ^19^F NMR (376 MHz, CDCl_3_) δ −66.4 (t, *J* = 11.0 Hz). HRMS (ESI) [M + H]^+^ calc. for C_22_H_42_O_3_F_3_, 411.30806, observed, 411.30807.

##### (4*S*)-2,2-Dimethyl-4-(18,18,18-trifluorooctadecoxymethyl)-1,3-dioxolane (**16b**)

Synthesis was carried out according to General Alkylation Procedure A section starting from intermediate **13b** (1.10 g, 2.30 mmol, 1.0 equiv) and (*S*)-(+)-2,2-dimethyl-1,3-dioxolane-4-methanol (0.304 g, 2.30 mmol, 1.0 equiv) and the resulting crude material was purified via column chromatography eluting along a gradient of 0–20% EtOAc in hexanes to yield a white solid (0.603 g, 1.44 mmol, 59% yield). ^1^H NMR (400 MHz, CDCl_3_) δ 4.26 (p, *J* = 6.0 Hz, 1H), 4.06 (dd, *J* = 8.2, 6.4 Hz, 1H), 3.73 (dd, *J* = 8.2, 6.4 Hz, 1H), 3.54–3.39 (m, 4H), 2.12–1.98 (m, 2H), 1.62–1.50 (m, 4H), 1.42 (s, 3H), 1.36 (s, 3H), 1.34–1.22 (m, 26H). ^13^C NMR (101 MHz, CDCl_3_) δ 127.1 (q, *J*_CF_ = 276.3 Hz), 109.2, 74.6, 71.7, 71.7, 66.8, 33.4 (q, *J*_CF_ = 27.3 Hz), 30.8, 29.7–28.9 (m, 11C), 28.5, 26.6, 25.9, 25.3, 21.7. ^19^F NMR (376 MHz, CDCl_3_) δ −66.4 (t, *J* = 11.0 Hz). HRMS (ESI) [M + H]^+^ calc. for C_24_H_46_O_3_F_3_, 439.33936, observed, 439.34005.

##### (2*S*)-3-(16,16,16-Trifluorohexadecoxy)­propane-1,2-diol (**17a**)

Synthesis was carried out according to the General Acetonide Deprotection Procedure section starting from intermediate **16a** (1.00 g, 2.44 mmol, 1.0 equiv), and the resulting crude material was purified via column chromatography eluting along a gradient of 0–100% EtOAc in hexanes to yield a white solid (0.523 g, 1.42 mmol, 58% yield). ^1^H NMR (400 MHz, CDCl_3_) δ 3.90–3.81 (m, 1H), 3.77–3.61 (m, 2H), 3.58–3.41 (m, 4H), 2.12–1.98 (m, 2H), 1.59 (m, 4H), 1.40–1.20 (m, 22H). ^13^C NMR (101 MHz, CDCl_3_) δ 126.1 (q, *J*_CF_ = 276.5 Hz), 72.7, 72.0, 70.5, 64.5, 34.0 (q, *J*_CF_ = 29.5 Hz), 29.8 (7C), 29.6, 29.5, 29.3, 28.8, 26.2, 22.0 (q, *J*_CF_ = 2.8 Hz). ^19^F NMR (376 MHz, CDCl_3_) δ −66.4 (t, *J* = 10.8 Hz). HRMS (ESI) [M + Na]^+^ calc. for C_19_H_37_O_3_F_3_Na, 393.4840, observed, 393.2585.

##### (2*S*)-3-(18,18,18-Trifluorooctadecoxy)­propane-1,2-diol (**17b**)

Synthesis was carried out according to the General Acetonide Deprotection Procedure section starting from intermediate **16b** (0.500 g, 1.11 mmol, 1.0 equiv) and the resulting crude material was purified via column chromatography eluting along a gradient of 0–100% EtOAc in hexanes to yield a white solid (0.291 g, 0.730 mmol, 64% yield). ^1^H NMR (400 MHz, CDCl_3_) δ 3.89–3.83 (m, 1H), 3.69 (d, *J* = 16.7 Hz, 2H), 3.56–3.42 (m, 4H), 2.12–2.04 (m, 2H), 1.56 (m, 4H), 1.41–1.19 (m, 26H). ^13^C NMR (101 MHz, CDCl_3_) δ 127.0 (q, *J*_CF_ = 278.2 Hz), 72.2, 71.5, 70.1, 64.0, 33.5 (q, *J*_CF_ = 28.7 Hz), 29.5 (9C), 29.1, 29.0, 28.8, 28.4, 25.7, 21.5 (q, *J*_CF_ = 2.8 Hz). ^19^F NMR (376 MHz, CDCl_3_) δ −66.4 (t, *J* = 11.0 Hz). HRMS (ESI) [M]·^+^ calc. for C_21_H_41_O_3_F_3_, 398.3008, observed, 398.3012.

##### (2*R*)-1-[*tert*-Butyl­(dimethyl)­silyl]­oxy-3-(16,16,16-trifluorohexadecoxy)­propan-2-ol (**18a**)

Synthesis was carried out according to the General Silylation Procedure section starting from intermediate **17a** (0.500 g, 1.35 mmol, 1.0 equiv), and the resulting crude material was purified via column chromatography eluting along a gradient of 0–20% EtOAc in hexanes to yield a clear oil (0.360 g, 0.744 mmol, 55% yield). ^1^H NMR (600 MHz, CDCl_3_) δ 3.84–3.78 (m, 1H), 3.68–3.60 (m, 2H), 3.49–3.40 (m, 4H), 2.10–2.02 (m, 2H), 1.60–1.51 (m, 4H), 1.38–1.25 (m, 22H), 0.90 (s, 9H), 0.07 (s, 6H). ^13^C NMR (101 MHz, CDCl_3_) δ 127.6 (q, *J*_CF_ = 276.3 Hz), 71.8, 71.7, 70.8, 64.2, 33.9 (q, *J*_CF_ = 28.2 Hz), 29.9 (6C), 29.7, 29.7, 29.6, 29.3, 28.9, 26.3, 26.1 (3C), 22.0 (q, *J*_CF_ = 2.9 Hz), 17.6, −5.4, −5.4. ^19^F NMR (376 MHz, CDCl_3_) δ −66.4 (t, *J =* 11.0 Hz). HRMS (ESI) [M]^•+^ calc. for C_25_H_51_O_3_F_3_Si, 484.35630, observed, 484.35661.

##### (2*R*)-1-[*tert*-Butyl­(dimethyl)­silyl]­oxy-3-(18,18,18-trifluorooctadecoxy)­propan-2-ol (**18b**)

Synthesis was carried out according to the General Silylation Procedure section starting from intermediate **17b** (0.290 g, 0.730 mmol), and the resulting crude material was purified via column chromatography eluting along a gradient of 0–20% EtOAc in hexanes to yield a clear oil (0.273 g, 0.53 3 mmol, 73% yield). ^1^H NMR (400 MHz, CDCl_3_) δ 3.84–3.76 (m, 1H), 3.69–3.58 (m, 2H), 3.50–3.38 (m, 4H), 2.11–1.99 (m, 2H), 1.62–1.50 (m, 4H), 1.41–1.19 (m, 26H), 0.90 (s, 9H), 0.07 (s, 6H). ^13^C NMR (101 MHz, CDCl_3_) δ 127.4 (q, *J*_CF_ = 276.3 Hz), 71.8, 71.6, 70.8, 64.2, 33.9 (q, *J*_CF_ = 28.2 Hz), 29.8 (8C), 29.7, 29.6, 29.5, 29.3, 28.8, 26.3, 26.0 (3C), 22.0 (q, *J*_CF_ = 2.9 Hz), 18.4, −5.3, −5.4. ^19^F NMR (376 MHz, CDCl_3_) δ −66.4 (t, *J* = 11.0 Hz). [M + Na]^+^ calc. for C_27_H_55_NaO _3_F_3_Si, 534.36922, observed, 534.36987.

##### *tert*-Butyl-dimethyl-[(2*R*)-2-benzyloxy-3-(16,16,16-trifluorohexadecoxy)­propoxy]­silane (**19a**)

Synthesis was carried out according to the General Benzylation Procedure section starting from intermediate **18a** (0.300 g, 0.622 mmol, 1.0 equiv), and the resulting crude material was purified via column chromatography eluting along a gradient of 0–20% EtOAc in hexanes to yield a clear oil (0.253 g, 0.431 mmol, 69% yield). ^1^H NMR (400 MHz, CDCl_3_) δ 7.41–7.29 (m, 5H), 4.61 (dd, *J* = 11.3, 1.3 Hz, 2H), 3.69 (app q, *J* = 1.7 Hz, 1H), 3.67–3.53 (m, 2H), 3.53–3.39 (m, 4H), 2.11–1.97 (m, 2H), 1.60–1.52 (m, 4H), 1.27 (d, *J* = 11.4 Hz, 22H), 0.97–0.80 (m, 9H), 0.08 (s, 6H). ^13^C NMR (101 MHz, CDCl_3_) δ 139.1, 128.5, 128.4 (2C), 127.9 (2C), 127.4 (q, *J*_CF_ = 276.1 Hz), 79.0, 73.5, 72.4, 71.8, 63.3, 33.9 (q, *J*_CF_ = 28.3 Hz), 29.8 (6C), 29.7, 29.6, 29.5, 29.3, 28.9, 27.2, 26.0 (3C), 22.0 (q, *J*_CF_ = 2.6 Hz), 14.29, −5.3, −5.3. ^19^F NMR (376 MHz, CDCl_3_) δ −66.4 (t, *J =* 11.0 Hz). HRMS (ESI) [M + H]^+^ calc. for C_32_H_58_O_3_F_3_Si, 575.41018, observed, 575.41137.

##### *tert*-Butyl-dimethyl-[(2*R*)-2-benzyloxy-3-(18,18,18-trifluorooctadecoxy)­propoxy]­silane (**19b**)

Synthesis was carried out according to the General Benzylation Procedure section starting from intermediate **18b** (0.250 g, 0.491 mmol, 1.0 equiv), and the resulting crude material was purified via column chromatography eluting along a gradient of 0–20% EtOAc in hexanes to yield a clear oil (0.151 g, 0.252 mmol, 52% yield). ^1^H NMR (400 MHz, CDCl_3_) δ 7.37–7.33 (m, 5H), 4.67 (dd, *J* = 11.7, 1.2 Hz, 2H), 3.75 (app q, *J* = 6.7 Hz, 1H), 3.72–3.63 (m, 2H), 3.61–3.42 (m, 4H), 2.10–1.99 (m, 2H), 1.64–1.52 (m, 4H), 1.63–1.49 (m, 26H), 1.39–1.22 (m, 9H), −0.00 (s, 6H). ^19^F NMR (376 MHz, CDCl_3_) δ −66.4 (t, *J* = 11.0 Hz).

##### (2*S*)-2-Benzyloxy-3-(16,16,16-trifluorohexadecoxy)­propan-1-ol (**20a**)

Synthesis was carried out according to the General Silyl Deprotection Procedure section starting from intermediate **19a** (0.200 g, 0.351 mmol, 1.0 equiv), and the resulting crude material was purified via column chromatography eluting along a gradient of 0–50% EtOAc in hexanes and concentrated down to yield a clear oil (0.101 g, 0.224 mmol, 63% yield). ^1^H NMR (400 MHz, CDCl_3_) δ 7.28–7.17 (m, 5H), 4.57–4.44 (m, 2H), 3.70–3.61 (m, 1H), 3.62–3.53 (m, 2H), 3.53–3.41 (m, 2H), 3.34 (td, *J* = 6.6, 1.6 Hz, 2H), 2.04–1.90 (m, 2H), 1.53–1.40 (m, 4H), 1.30–1.11 (m, 22H). ^13^C NMR (101 MHz, CDCl_3_) δ 175.6, 138.7, 128.8 (2C), 128.2 (2C), 128.0 (q, *J*_CF_ = 279.3 Hz), 78.3, 72.5, 72.3, 71.5, 63.4, 34.1 (q, *J*_CF_ = 28.0 Hz), 31.1, 29.9 (6C), 29.9, 29.7, 29.6, 29.1, 26.5, 18.0. ^19^F NMR (376 MHz, CDCl_3_) δ −66.4 (t, *J =* 11.0 Hz). HRMS (ESI) [M + H]^+^ calc. for C_26_H_44_O_3_F_3_, 461.32371, observed, 461.32418.

##### (2*S*)-2-Benzyloxy-3-(18,18,18-trifluorooctadecoxy)­propan-1-ol (**20b**)

Synthesis was carried out according to the General Silyl Deprotection Procedure section starting from intermediate **19b** (0.150 g, 0.254 mmol), and the resulting crude material was purified via column chromatography eluting along a gradient of 0–50% EtOAc in hexanes and concentrated down to yield a clear oil (0.062 g, 0.104 mmol, 41% yield). ^1^H NMR (600 MHz, CDCl_3_) δ 7.37–7.28 (m, 5H), 4.74–4.60 (m, 2H), 3.76 (m, 1H), 3.72–3.63 (m, 2H), 3.61–3.51 (m, 2H), 3.48–3.39 (m, 2H), 2.11–2.00 (m, 2H), 1.60–1.50 (m, 4H), 1.37–1.23 (m, 26H).^13^C NMR (101 MHz, CDCl_3_) δ 175.3, 138.5, 128.4 (2C), 128.2 (2C), 128.0 (q, *J*_CF_ = 279.5 Hz), 78.3, 72.8, 72.9, 71.1, 63.9, 34.2 (q, *J*_CF_ = 28.0 Hz), 32.4, 29.9 (8C), 29.9, 29.6, 29.5, 29.1, 26.9, 18.7. ^19^F NMR (376 MHz, CDCl_3_) −66.4 (t, *J =* 11.1 Hz). HRMS (ESI) [M + H]^+^ calc. for C_28_H_48_O_3_F_3_, 489.35501, observed, 489.35605.

##### Ammonium­[(1*R*)-2-(6-Aminopurin-9-yl)-1-methyl-ethoxy]­methyl-[(2*S*)-2-benzyloxy-3-(16,16,16-trifluorohexadecoxy)­propoxy]­phosphinate (**21a**)

Synthesis was carried out according to the General TFV Coupling Procedure section starting from intermediate **20a** (0.100 g, 0.222 mmol, 1.0 equiv) and TFV **7** (0.063 g, 0.222 mmol, 1.0 equiv) to yield a white solid (0.041 g, 0.063 mmol, 26% yield). ^1^H NMR (400 MHz, CD_3_OD) δ 8.32 (s, 1H), 8.16 (s, 1H), 7.29–7.10 (m, 5H), 4.59 (s, 2H), 4.32 (dd, *J* = 14.5, 2.9 Hz, 1H), 4.12 (dd, *J* = 14.4, 6.7 Hz, 1H), 4.01–3.89 (m, 3H), 3.78–3.67 (m, 2H), 3.62–3.44 (m, 3H), 3.37 (t, *J* = 6.5 Hz, 2H), 2.13–2.00 (m, 2H), 1.52–1.44 (m, 4H), 1.37–1.18 (m, 22H), 1.06 (d, *J* = 6.2 Hz, 3H). ^13^C NMR (151 MHz, CD_3_OD) δ 151.9, 149.8, 145.9, 145.7, 139.6, 128.8 (2C), 128.4 (2C), 128.1, 127.6 (q, *J*_CF_ = 275.1 Hz), 118.7, 78.5, 76.0 (d, *J*_CP_ = 11.5 Hz), 72.7, 72.2, 71.1, 65.3, 65.2, 64.4 (d, *J* = 6.2 Hz), 34.0 (q, *J*_CF_ = 28.2 Hz), 30.430.3 (m, 6C), 30.3, 30.2, 30.1, 29.9, 29.3, 26.9, 22.6 (q, *J* = 2.6 Hz), 16.6. ^19^F NMR (376 MHz, CD_3_OD): δ −68.0 (t, *J* = 11.2 Hz). ^31^P NMR (162 MHz, CD_3_OD): δ 17.2. HRMS (ESI) [M + H]^+^ calc. for C_35_H_56_O_6_N_5_F_3_P, 730.39111, observed, 730.39148. LC-MS (ESI, C8, 0.5 mL/min) 75–95% ACN in H_2_O, 6 min, rt = 3.986, *m*/*z* = 730.4 [M + H]^+^ and 728.3 [M – H]^−^.

##### Ammonium­[(1*R*)-2-(6-aminopurin-9-yl)-1-methyl-ethoxy]­methyl-[(2*S*)-2-benzyloxy-3-(18,18,18-trifluorooctadecoxy)­propoxy]­phosphinate (**21b**)

Synthesis was carried out according to the General TFV Coupling Procedure section starting from intermediate **20b** (0.60 g, 0.121 mmol, 1.0 equiv) and TFV **7** (0.035 g, 0.121 mmol, 1.0 equiv) to yield a white solid (0.032 g, 0.042 mmol, 34% yield). ^1^H NMR (400 MHz, CD_3_OD) δ 8.40 (s, 1H), 8.23 (s, 1H), 7.37–7.17 (m, 5H), 4.66 (br s, 2H), 4.46–4.36 (m, 1H), 4.20 (dd, *J* = 14.4, 6.6 Hz, 1H), 4.06–3.93 (m, 3H), 3.86–3.72 (m, 2H), 3.68–3.50 (m, 3H), 3.44 (t, *J* = 6.5 Hz, 2H), 2.22–2.05 (m, 2H), 1.61–1.49 (m, 4H), 1.44–1.26 (m, 26H), 1.13 (d, *J* = 6.2 Hz, 3H). ^13^C NMR (151 MHz, CD_3_OD): δ 151.5, 148.8, 145.5, 144.7, 138.7, 127.8 (2C), 127.5 (q, *J*_CF_ = 274.8 Hz), 127.4 (2C), 127.1, 117.8, 77.6, 75.1 (d, *J*_CP_ = 11.1 Hz), 71.7, 71.2, 70.2, 64.3, 64.2, 63.5 (d, *J* = 6.9 Hz), 33.0 (q, *J*_CF_ = 28.1 Hz), 29.5 – 29.3 (m, 8C), 29.3, 29.2, 29.1, 28.9, 28.4, 25.9, 21.6 (q, *J* = 3.1 Hz), 15.6. ^19^F NMR (376 MHz, CD_3_OD): δ −68.0 (t, *J* = 11.2 Hz). ^31^P NMR (162 MHz, CD_3_OD): δ 17.0. HRMS (ESI) [M + H]^+^ calc. for C_37_H_60_O_6_N_5_F_3_P, 758.42212, observed, 758.42278. LC-MS (ESI, C8, 0.5 mL/min) 50–95% ACN in H_2_O, 6 min, rt = 4.499, *m*/*z* = 758.5 [M + H]^+^ and 756.5 [M – H]^−^; 75–95% ACN in H_2_O, 6 min, rt = 1.415, *m*/*z* = 758.5 [M + H]^+^ and 756.5 [M – H]^−^.

## Supplementary Material



## Notes

Data for CM, antiviral, and LC-MS/MS assays, reference chemical structures, ^1^H and ^13^C NMR spectra for final compounds, LC-MS traces for final compounds, and associated references (PDF)

## Abbreviations

ATP: adenosine triphosphate
AUC: area under the curve
BOG: benzyloxyglycerol
BUP: buprenorphine
CAB: cabazitaxel
CCR5: CC-type chemokine receptor 5
CDV: cidofovir
CHOL: cholesterol
CM: chylomicron
CXCR4: CXC-type chemokine receptor 4
CYP: cytochrome P450
DCC: *N*,*N*′-dicyclohexylcarbodiimide
DCM: dichloromethane
DMAP: *N*,*N*-dimethylaminopyridine
DMPU: *N*,*N*′-dimethylpropylene urea
DTX: docetaxel
ELISA: enzyme-linked immunosorbent assay
ER: endoplasmic reticulum
GI: gastrointestinal
HBV: hepatitis B virus
HDP: hexadecyloxypropyl
HIV: human immunodeficiency virus
HLM: human liver microsomes
ITSD: internal standard
LDL: low-density lipoprotein
MET: metformin
MLM: mouse liver microsomes
MPA: mycophenolic acid
MTT: 3-(4,5-dimethyl-2-thiazolyl)-2,5-diphenyl-2*H*-tetrazolium bromide (thiazole blue)
NADPH: nicotinamide adenine dinucleotide phosphate
NMP: *N*-methyl-2-pyrrolidone
NtRTI: nucleotide reverse transcriptase inhibitor
OA: oleic acid
ODBG: octadecyl-*sn*-2-benzyloxyglycerol
ODP: octadecyloxypropyl
PBS: phosphate-buffered saline
PK: pharmacokinetic
PL: phospholipid
PMB: *para*-methoxybenzyl
rt: room temperature (or retention time)
SARS-CoV-2: severe acute respiratory syndrome coronavirus 2
SEM: standard error of the mean
TAF: tenofovir alafenamide fumarate
TBAB: tetrabutylammonium bromide
TBS: *tert*-butyldimethylsilyl
TC: taurocholate
TDF: tenofovir disoproxil fumarate
TEM: transmission electron microscopy
TFV: tenofovir
TG: triglyceride
THF: tetrahydrofuran
TU: testosterone undecanoate
TXL: tenofovir exalidex
VLDL: very low-density lipoprotein
