# Supplementary material for: Next-Generation Lipid Prodrugs Orally Deliver Tenofovir via Enhanced Chylomicron Incorporation
Source: ACS Pharmacol Transl Sci. 2025 Aug 22;8(9):3047–73. doi: 10.1021/acsptsci.5c00237 (PMC12441861; doi:10.1021/acsptsci.5c00237)
Supplement: Supplementary file 1 [file pt5c00237_si_001.pdf]

SUPPORTING INFORMATION

Next-Generation Lipid Prodrugs Orally Deliver Tenofovir via Enhanced Chylomicron Incorporation

Hannah B. Gold[1], Nicole Pribut[1], Esther L. Outtrim[1], Priscilla Davidson[1,2], Christopher M. Monaco[1], August Myers[3,4], Carrie Qi Sun[2], Goknil Pelin Coskun[1], Andrea Mancia[1], Yanli Yang[1], Samantha Burton[1], Areeb Aftab[3,4], Cynthia A. Derdeyn[3,4], Rebecca S. Arnold[2], John A. Petros[2], Ken Liu[1], Eric J. Miller[5], Dennis C. Liotta[1]*.*

[1]Department of Chemistry, Emory University College of Arts & Sciences, Atlanta, GA 30322, USA

[2]Department of Urology, Emory University School of Medicine, Atlanta, GA 30322, USA

[3]Department of Laboratory Medicine & Pathology, University of Washington School of Medicine, Seattle, WA 98195, USA

[4]Washington National Primate Research Center, Infectious Diseases & Translational Medical Unit, Seattle, WA 98195, USA

[5]Department of Pharmacology & Chemical Biology, Emory University School of Medicine, Atlanta, GA 30322, USA

*Corresponding Authors: Eric J. Miller (ejmill2@emory.edu) and Dennis C. Liotta (dliotta@emory.edu)

TABLE OF CONTENTS

Supplementary Data For Bio Assays ____ S3-7

Chemical Structures Reference Figure ____ S3

Chylomicron Assays ____ S4

Anti-HIV Activity Assays ____ S4–8

LC-MS Scan Parameters for Test Compounds ____ S9

Final Compound ^{1}H and ^{13}C NMR Spectra ____ S10–S33

Final Compound LC/MS Traces ____ S34–S40

References ____ S41

SUPPLEMENTARY DATA FOR BIO ASSAYS

Chemical Structures Reference Figure

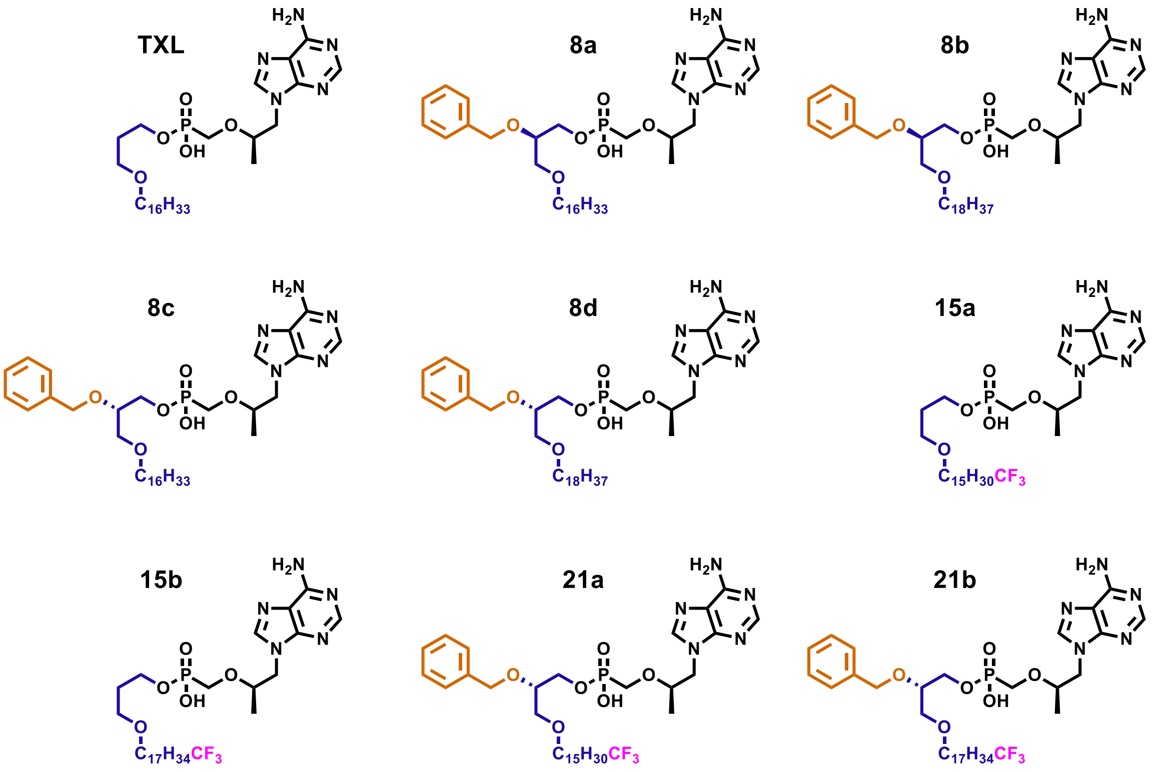

Figure S1. Chemical structures of TFV prodrugs referenced in this study.

Chylomicron Assays

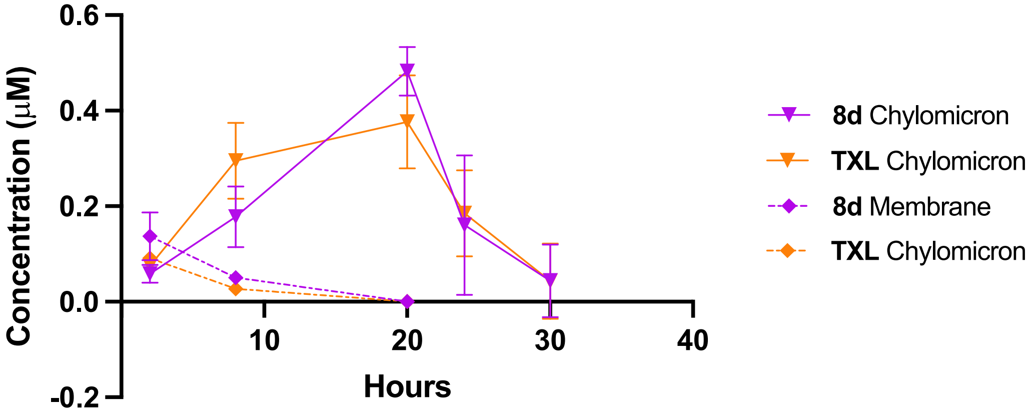

Figure S2. Extended chylomicron time course assay. Concentration of compounds **TXL** and **8d** in Caco-2 membrane and chylomicron isolate at 2, 8, 20, 24 and 30 hours (n = 3 replicates per timepoint).

Comparative Anti-HIV Activity Assays

Compound ID	HIV IC_{50} (previously reported)	Fold Change from TXL
**TXL**	0.011	1
**15a**	0.054	4.9

Table S1. Previously reported IC_{50} values[1] for compounds **15a** and **TXL**, along with their corresponding fold-change.

Compound ID	Lipid Motif	HIV IC_{50} (μM)	Fold Change from TXL
TXL	$C_3H_6OC_{16}H_{33}$	0.001	1
8a	$CH_2CH(\mathit{S})OBnCH_2OC_{16}H_{33}$	0.001	1
8b	$CH_2CH(\mathit{R})OBnCH_2OC_{18}H_{37}$	0.001	1
8c	$CH_2CH(\mathit{R})OBnCH_2OC_{16}H_{33}$	0.002	2
8d	$CH_2CH(\mathit{S})OBnCH_2OC_{18}H_{37}$	0.002	2

Table S2. HIV IC_{50} values and fold-change relative to **TXL**.

Several factors likely contribute to the variability observed in the IC_{50} values reported for **TXL** (**Tables S1-S3**). These include differences in solubility and stability of the compounds, experimental conditions, storage conditions, and timing of experiments. Variations in the dilution series and concentrations tested can also influence assay sensitivity and reproducibility.

Compound ID	Lipid Motif	HIV IC_{50} (μM)	Fold Change from TXL
TXL	$C_3H_6OC_{16}H_{33}$	0.21	1
15a	$C_3H_6OC_{15}H_{30}CF_3$	>1	4.8
15b	$C_3H_6OC_{17}H_{34}CF_3$	0.6	2.9
21a	$CH_2CH(\mathit{S})OBnCH_2OC_{15}H_{30}CF_3$	0.04	0.2
21b	$CH_2CH(\mathit{S})OBnCH_2OC_{17}H_{34}CF_3$	0.07	0.3

Table S3. HIV IC_{50} values and fold-change relative to **TXL**.

Additionally, the TZM-bl assay is a single-round infection assay designed to measure inhibitors blocking viral entry into the cell,[2, 3] thus a different efficacy assay allowing for multiple replication cycles may be better suited to measure reverse transcriptase inhibition by our prodrugs. Further, reverse transcription can initiate in the virion prior to cell entry, potentially affecting the efficacy of **TXL** depending on the timing of drug exposure.[4] Another factor affecting these assays is the requirement for **TXL** to enter the cell to reach its active form, which can add complexity compared to direct-entry inhibitors. Given these factors, caution is necessary when comparing IC_{50} values across different experiments *in vitro*. As a result, **TXL** has been used as an internal control here to normalize potency assessments.

Formulation	IC_{50} (μM)
BSA (+) FA	>10
BSA (-) FA	>10
HSA (-) FA	>10
BSA (+) FA (+) TXL	0.04
BSA (-) FA (+) TXL	0.02
HSA (-) FA (+) TXL	0.01
HSA (-) FA (+) TXL	0.03

Table S4. IC_{50} of TXL in different serum albumin formulations. BSA = bovine serum albumin. FA = fatty acids. HSA = human serum albumin.

In this antiviral activity assay, prodrugs are prepared in serum albumin (either bovine, BSA, or human, HSA), which is the most abundant plasma protein. Albumin has been shown to bind and

solubilize lipid prodrugs, providing a biologically relevant environment that mirrors *in vivo* conditions.[5] In circulation, an equilibrium between albumin-bound and free drug determines the prodrug's distribution and availability for cellular uptake and subsequent viral inhibition.[6] To assess whether differences in serum albumin formulation contributed to the variability observed in this assay, we evaluated **TXL**'s antiviral activity in different drug formulations using BSA and HSA, both with and without added fatty acids (**Table S4**). Serum albumin can bind both drugs and endogenous fatty acids, which can affect the unbound concentration of drug in the assay. We hypothesized that the presence or absence of fatty acids could alter how much drug is bound versus free, potentially affecting the measured antiviral activity. However, IC_{50} values remained relatively consistent across conditions, indicating that serum albumin formulation was not a significant source of variability. Also, as shown in **Table S1**, the relative potency of **15a** when compared with **TXL** observed in our previously reported data[1] is consistent with the relative potency measured in this study (**Table S3**). This supports our comparison to **TXL** despite the variations in absolute IC_{50}.

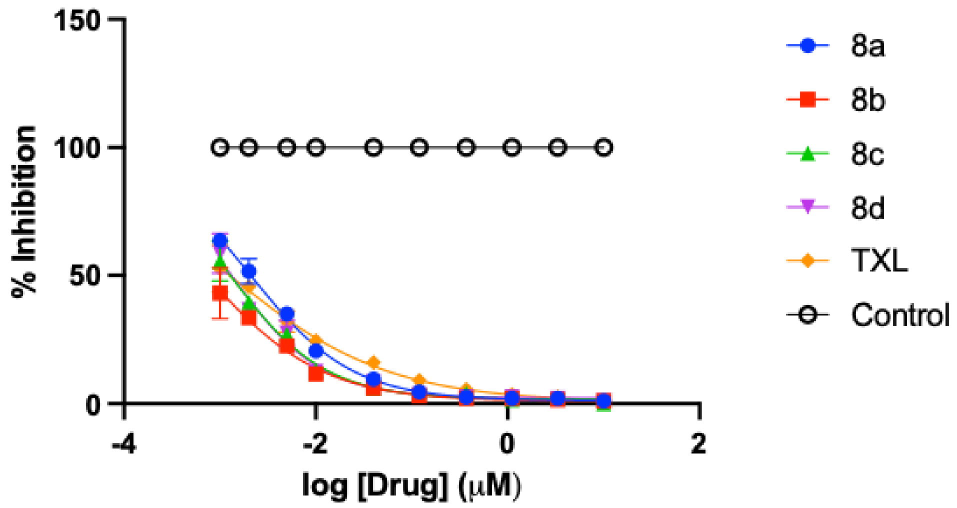

Figure S3. Log-transformed dose-response curve in TZM-bl cells infected with HIV pseudovirus and treated with non-CF_3 benzyloxylipid TFV prodrugs.

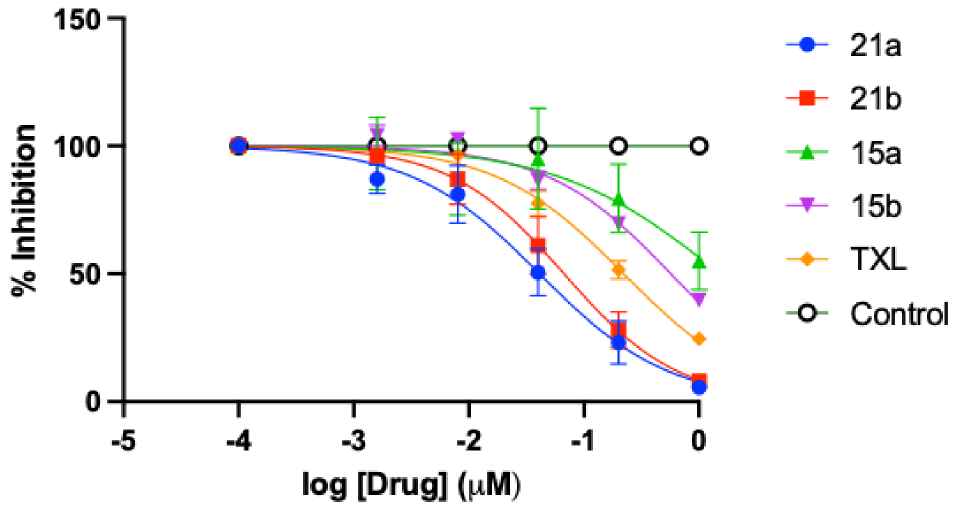

Figure S4: Log-transformed dose-response curve in TZM-bl cells infected with HIV pseudovirus and treated with CF_3 benzyloxylipid TFV prodrugs.

Compound Name	Precursor Ion MS1	Product Ion MS2	Dwell (ms)	Fragmentor Voltage (V)	Collision Energy (V)	Cell Accelerator (v)	Polarity
**8a**	676.4	658.4	100	170	29	4	Positive
	676.4	378	100	170	37	4	Positive
**8b**	676.4	378	100	170	37	4	Positive
	676.4	360	100	170	33	4	Positive
**8c**	704.5	686.4	100	150	33	4	Positive
	704.5	378	100	150	37	4	Positive
**8d**	704.5	378	100	150	37	4	Positive
	704.5	360	100	150	37	4	Positive
**15a**	624.4	270.1	100	200	37	4	Positive
	624.4	176.1	100	200	69	4	Positive
**15b**	652.44	270	100	110	41	4	Positive
	652.44	206.1	100	110	50	4	Positive
**21a**	730.39	360.1	100	170	37	4	Positive
	730.39	91.1	100	170	65	4	Positive
**21b**	758.43	360.1	100	138	41	4	Positive
	758.43	91.1	100	138	69	4	Positive

Table S5. Scan parameters for the tested compounds and associated transitions in MRM mode.

FINAL COMPOUND ^{1}H AND ^{13}C NMR SPECTRA

Ammonium[(1R)-2-(6-aminopurin-9-yl)-1-methyl-ethoxy]methyl-[(2S)-2-benzyloxy-3-hexadecoxy-propoxy]phosphinate ***(8a)***. ^{1}H NMR (400 MHz, CD_3OD), ^{13}C NMR (151 MHz, CD_3OD), ^{31}P NMR (162 MHz, CD_3OD).

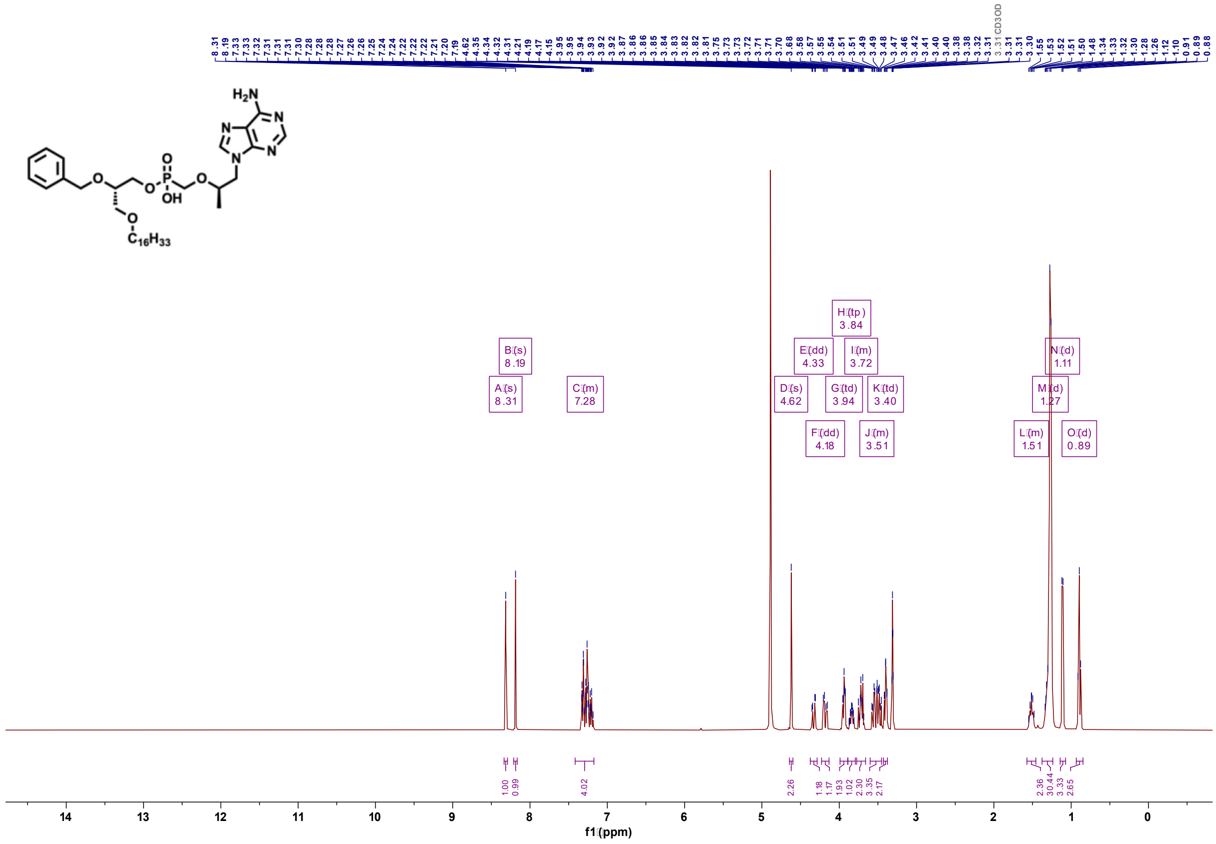

Figure S5. 8a ^{1}H NMR (400 MHz, CD_3OD).

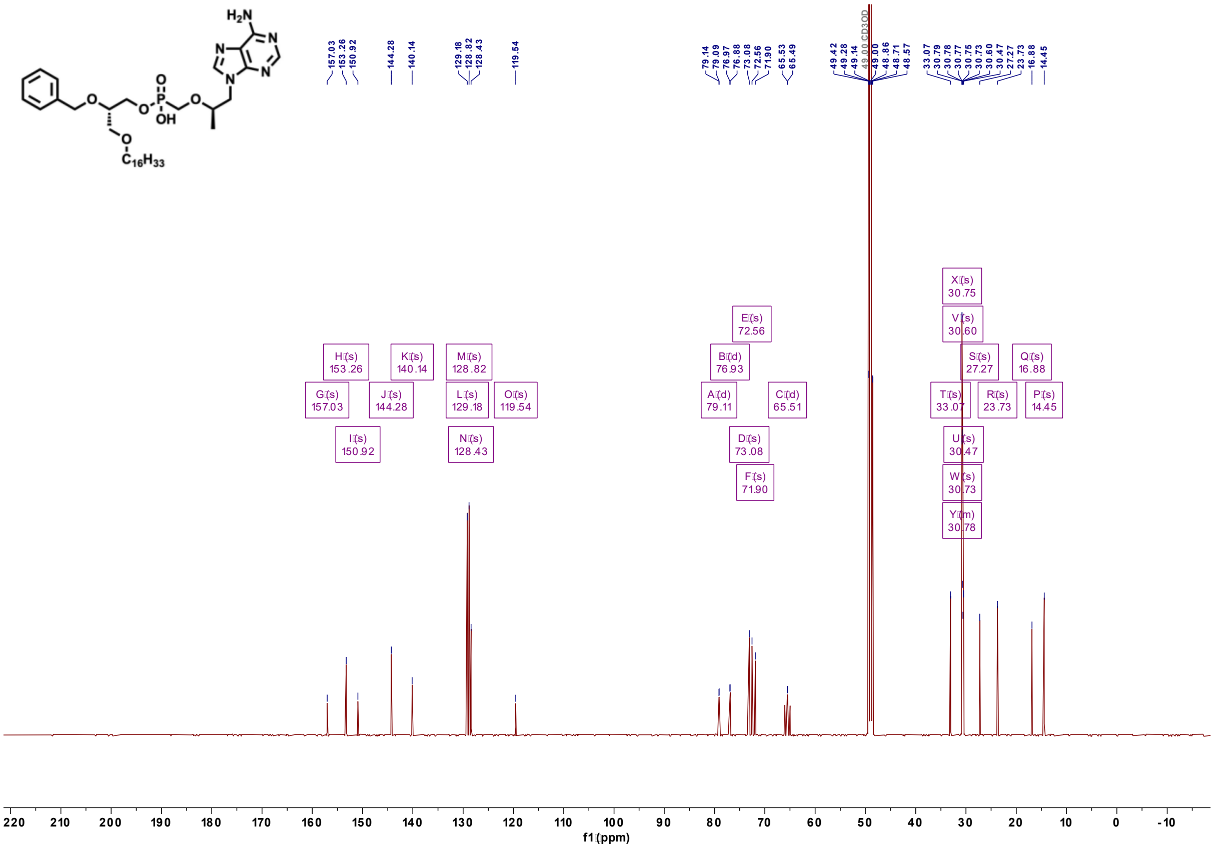

Figure S6. 8a ^{13}C NMR (151 MHz, CD_3OD).

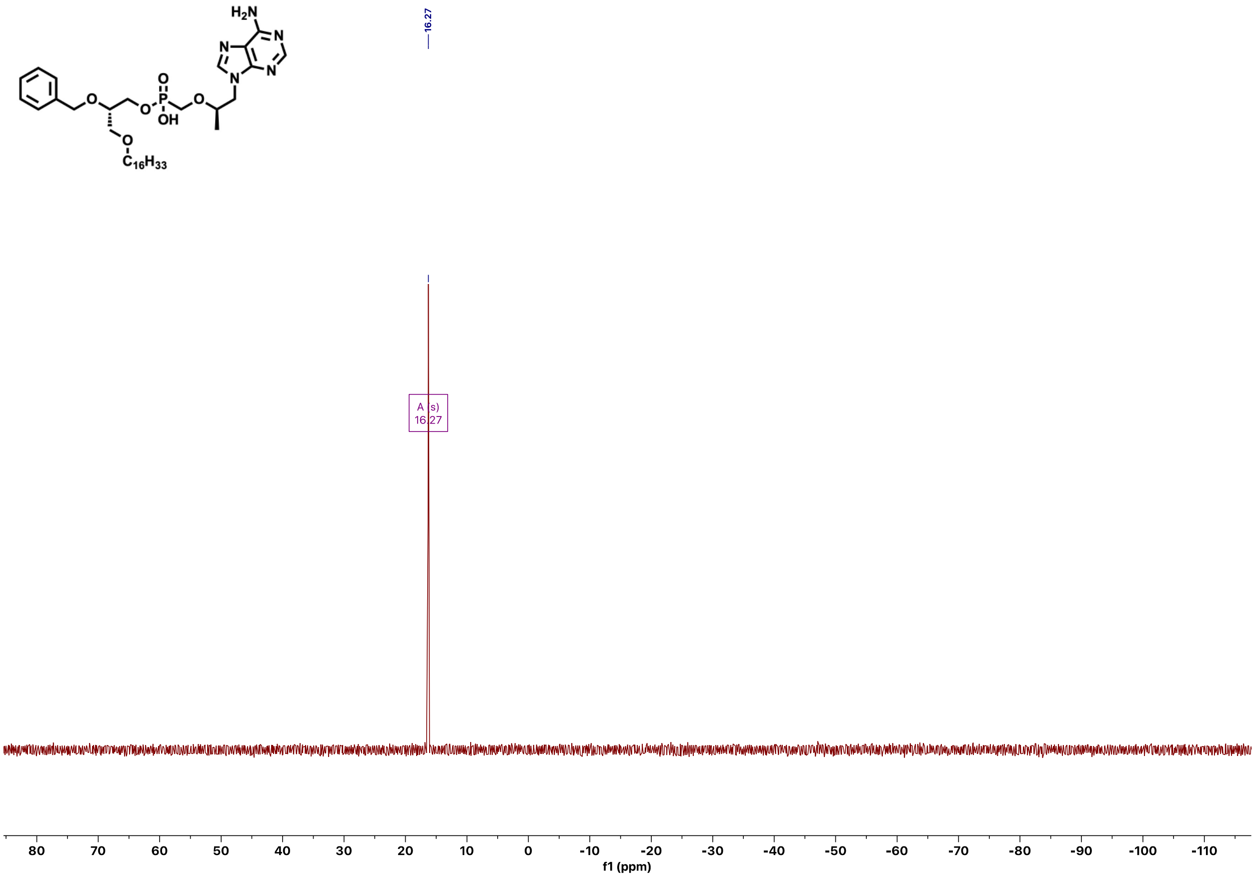

Figure S7. 8a ^{31}P NMR (162 MHz, CD_3OD).

Ammonium[(1R)-2-(6-aminopurin-9-yl)-1-methyl-ethoxy]methyl-[(2S)-2-benzyloxy-3-octadecoxy-propoxy]phosphinate (***8b***). ^{1}H NMR (600 MHz, CD_3OD), ^{13}C NMR (101 MHz, CD_3OD),^{31}P NMR (162 MHz, CD_3OD).

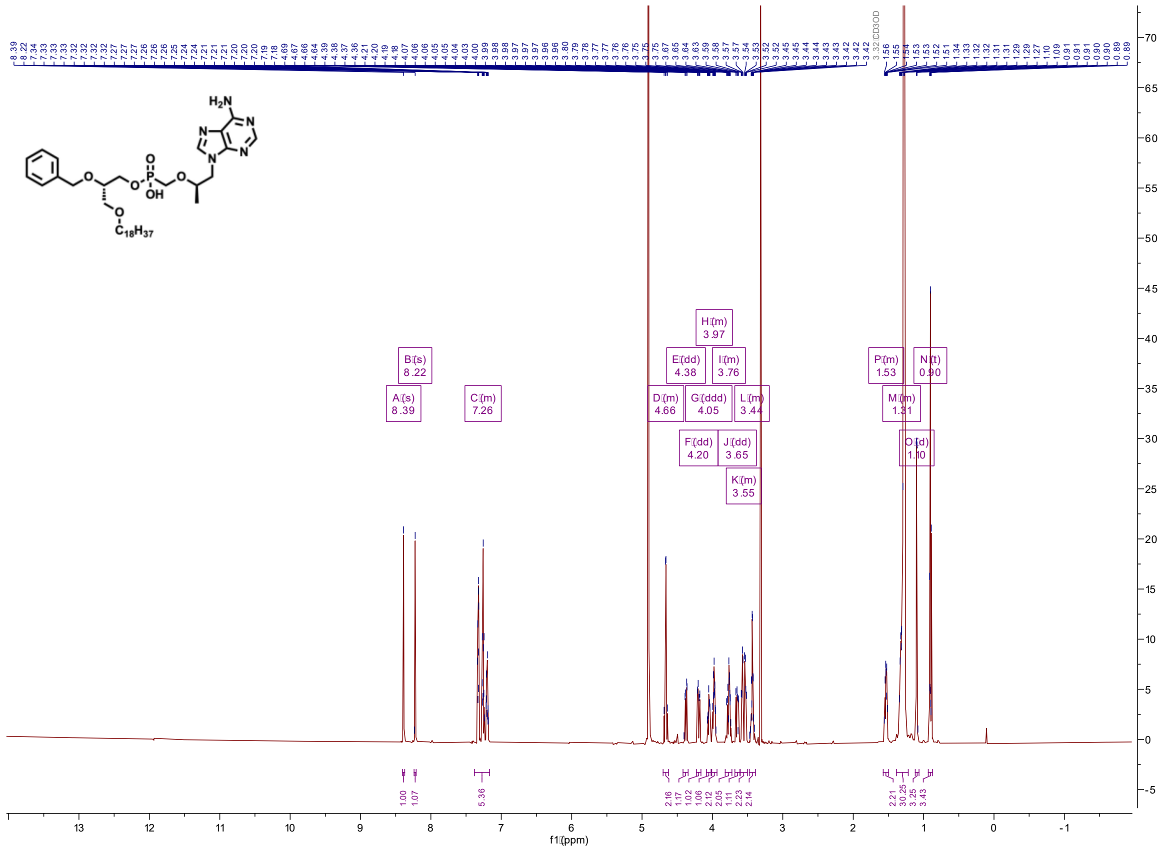

Figure S8. 8b ^{1}H NMR (600 MHz, CD_3OD).

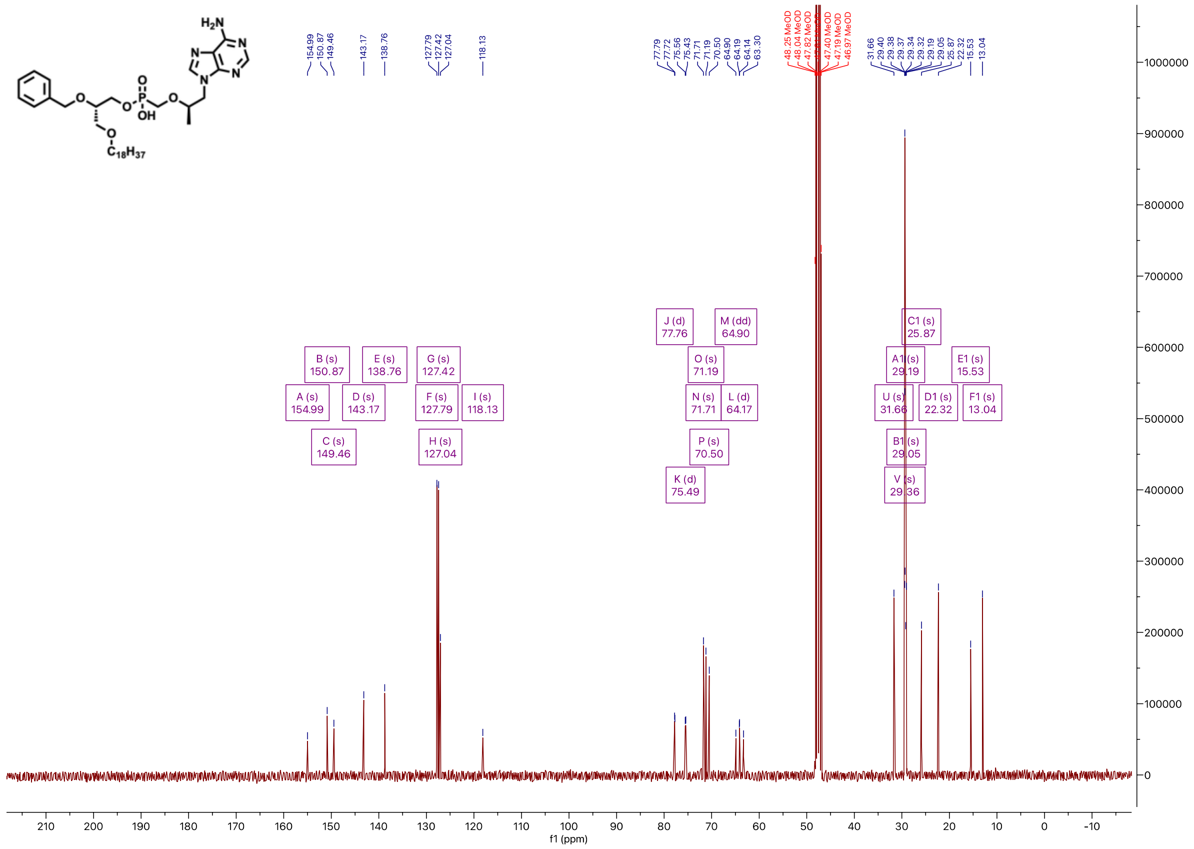

Figure S9. 8b ^{13}C NMR (101 MHz, CD_3OD).

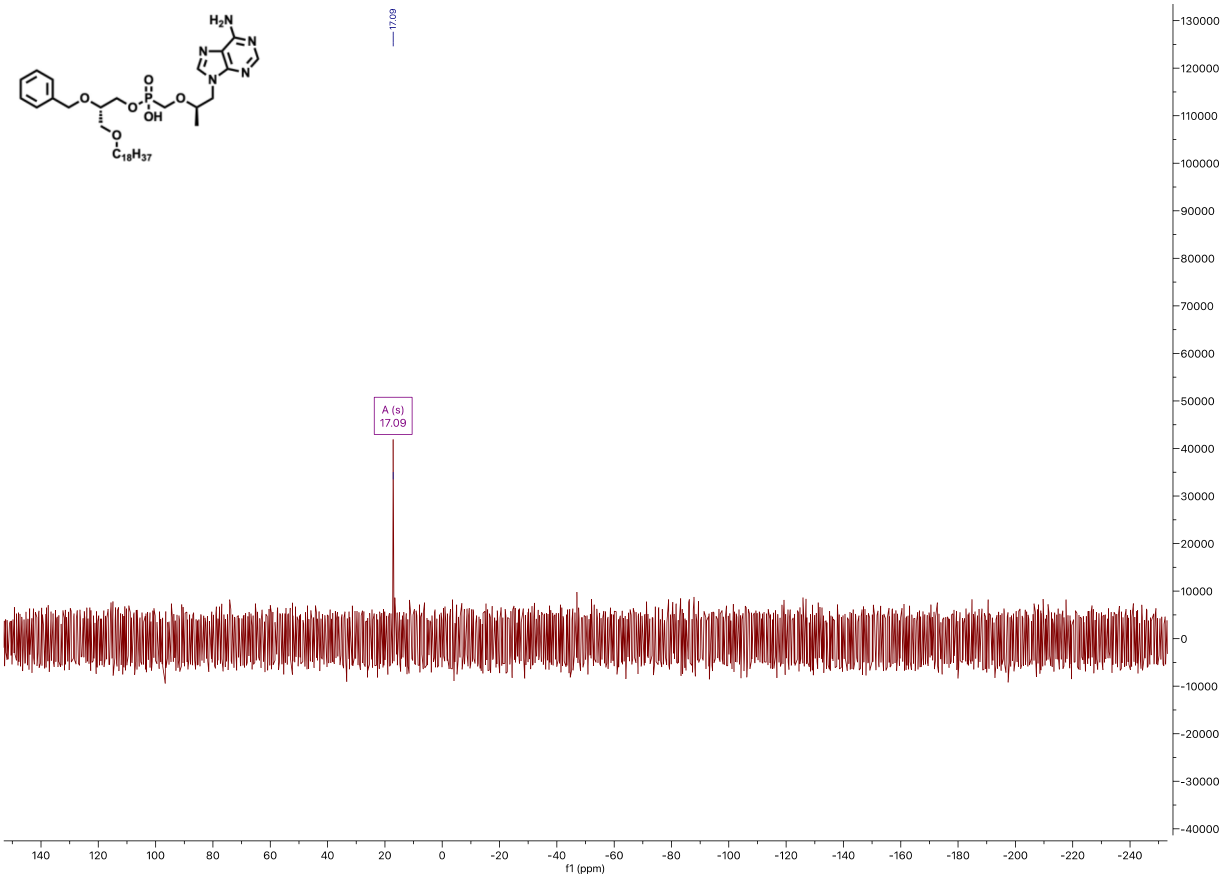

Figure S10. 8b ^{31}P NMR (162 MHz, CD_3OD).

*Ammonium[(1R)-2-(6-aminopurin-9-yl)-1-methyl-ethoxy]methyl-[(2R)-2-benzyloxy-3-hexadecoxy-propoxy]phosphinate (**8c**)*. ^{1}H NMR (400 MHz, CD_3OD), ^{13}C NMR (151 MHz, CD_3OD), ^{31}P NMR (162 MHz, CD_3OD).

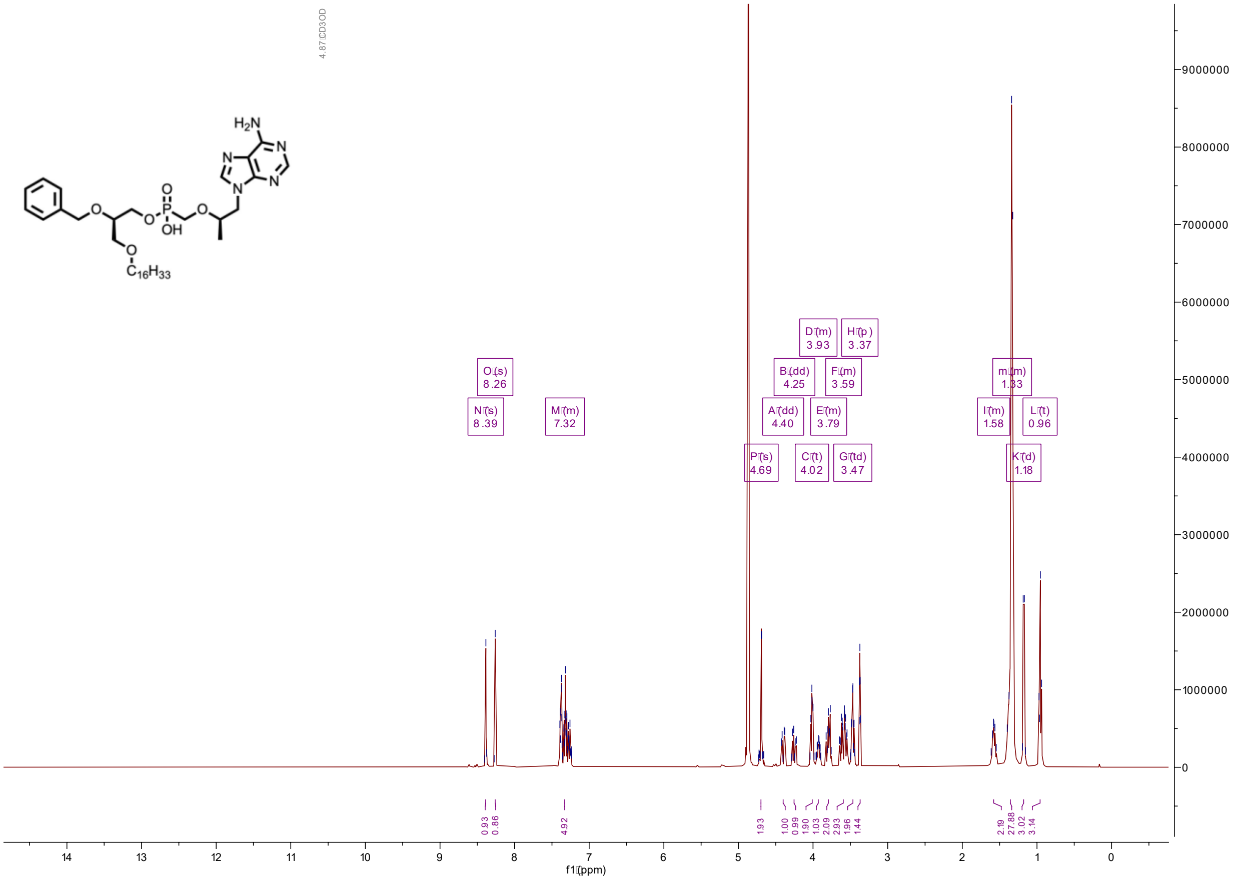

Figure S11. 8c ^{1}H NMR (400 MHz, CD_3OD).

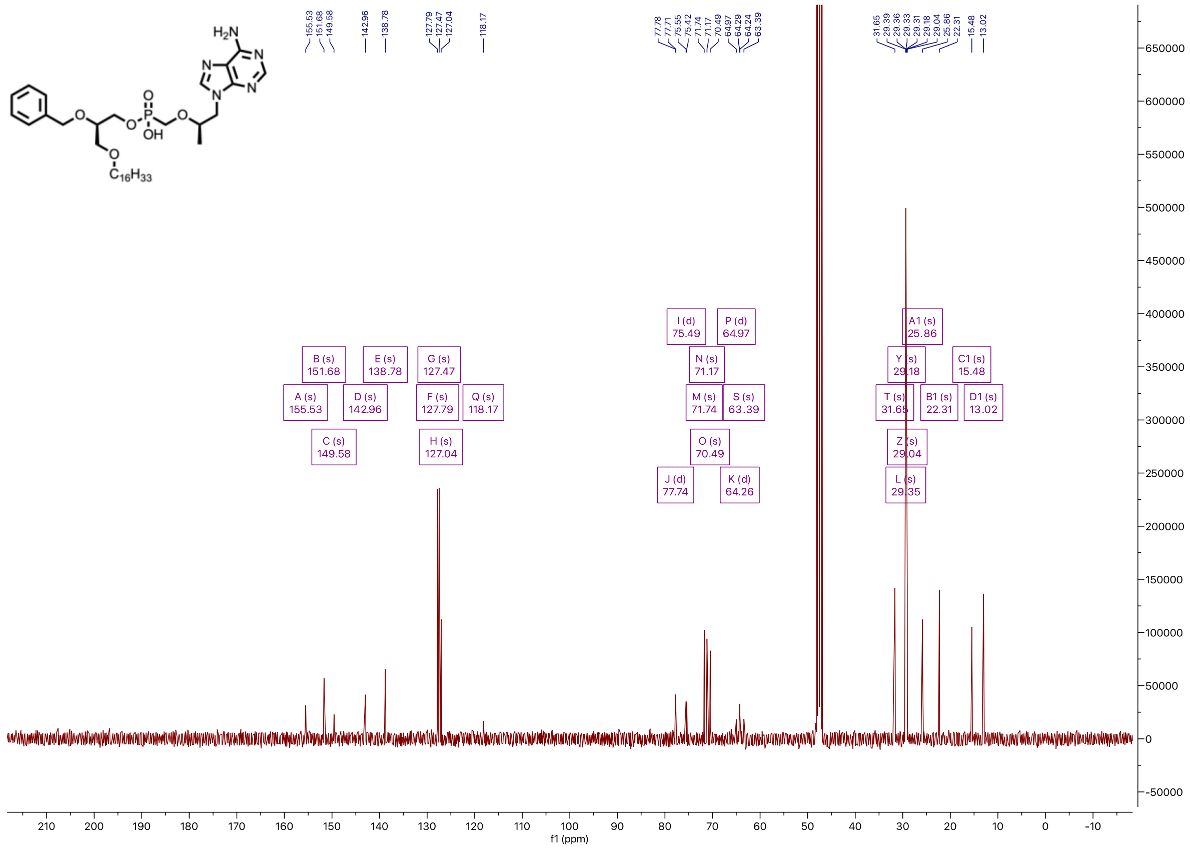

Figure S12. 8c ^{13}C NMR (151 MHz, CD_3OD).

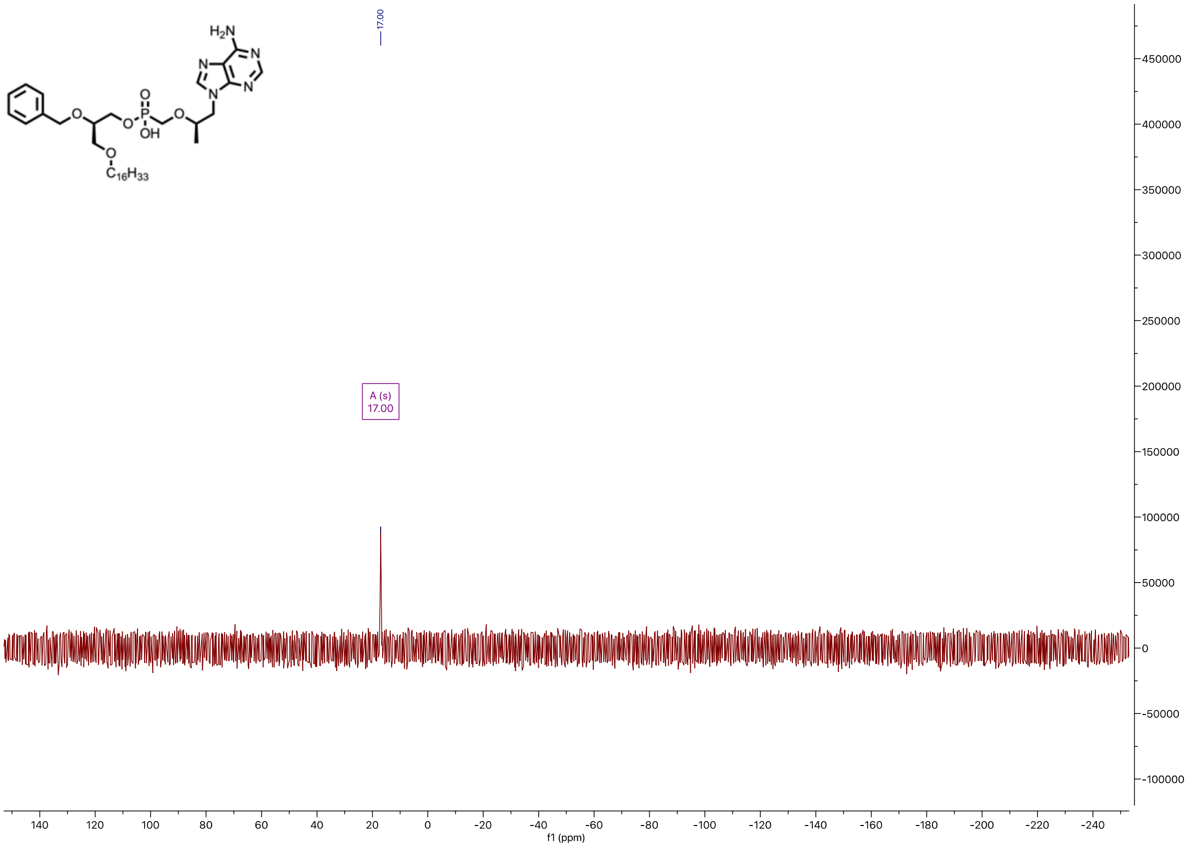

Figure S13. 8c ^{31}P NMR (162 MHz, CD_3OD).

*Ammonium[(1R)-2-(6-aminopurin-9-yl)-1-methyl-ethoxy]methyl-[(2R)-2-benzyloxy-3-octadecoxy-propoxy]phosphinate (**8d**).* ^{1}H NMR (600 MHz, CD_3OD), ^{13}C NMR (101 MHz, CD_3OD), ^{31}P NMR (162 MHz, CD_3OD).

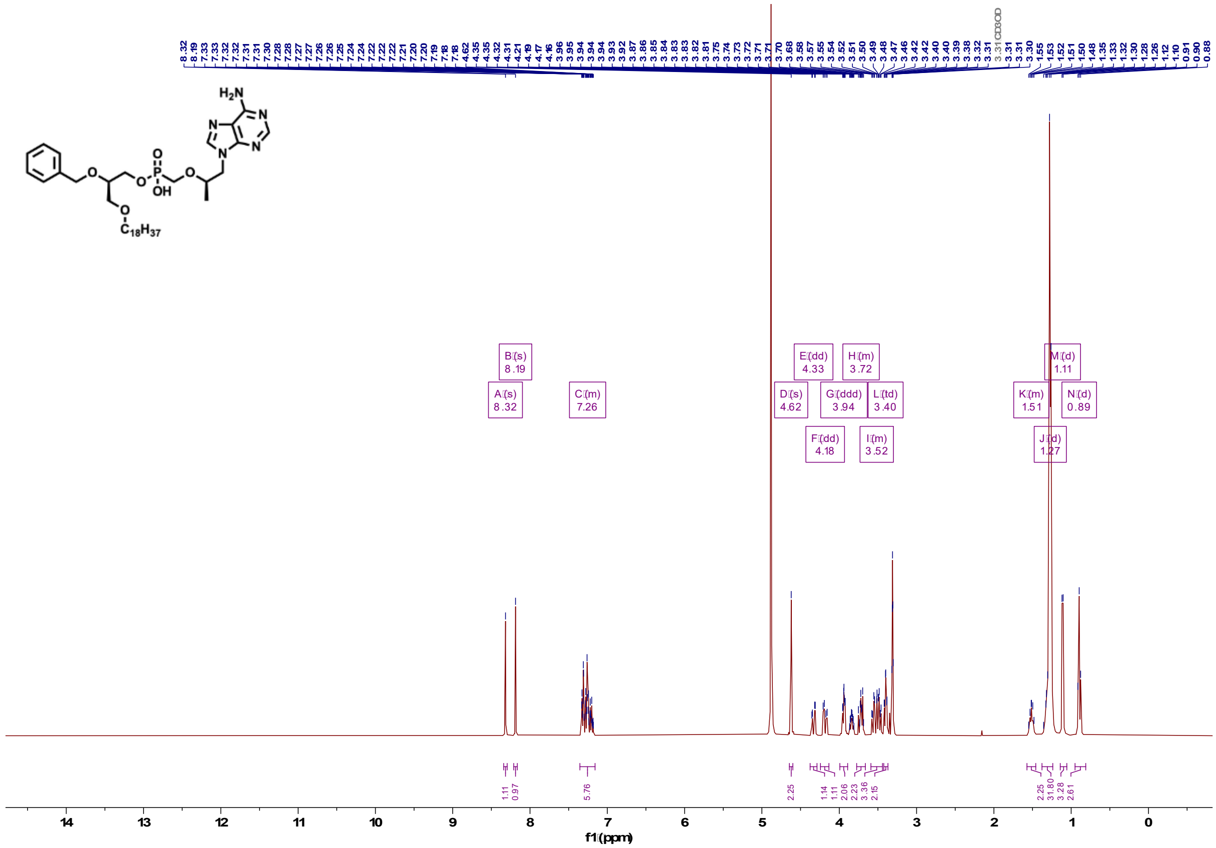

Figure S14. 8d ^{1}H NMR (600 MHz, CD_3OD).

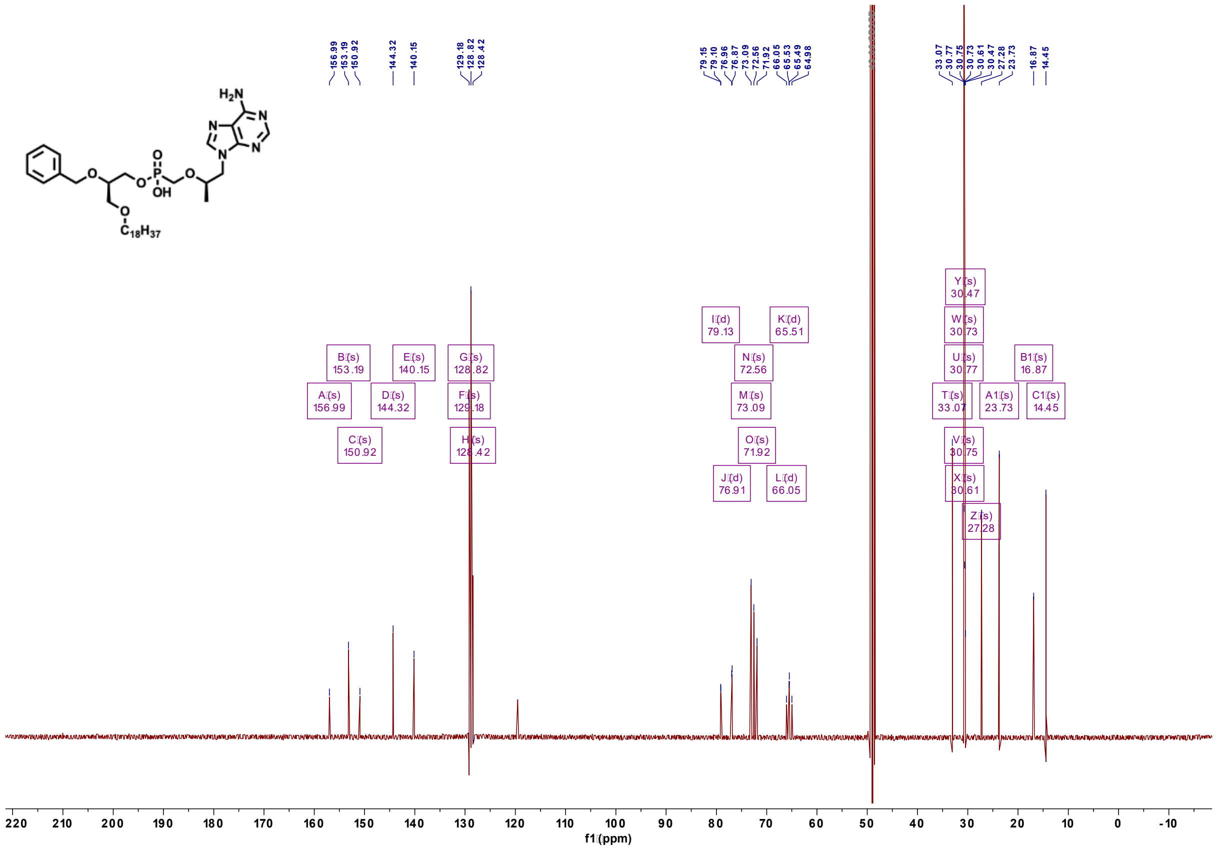

Figure S15. 8d ^{13}C NMR (101 MHz, CD_3OD).

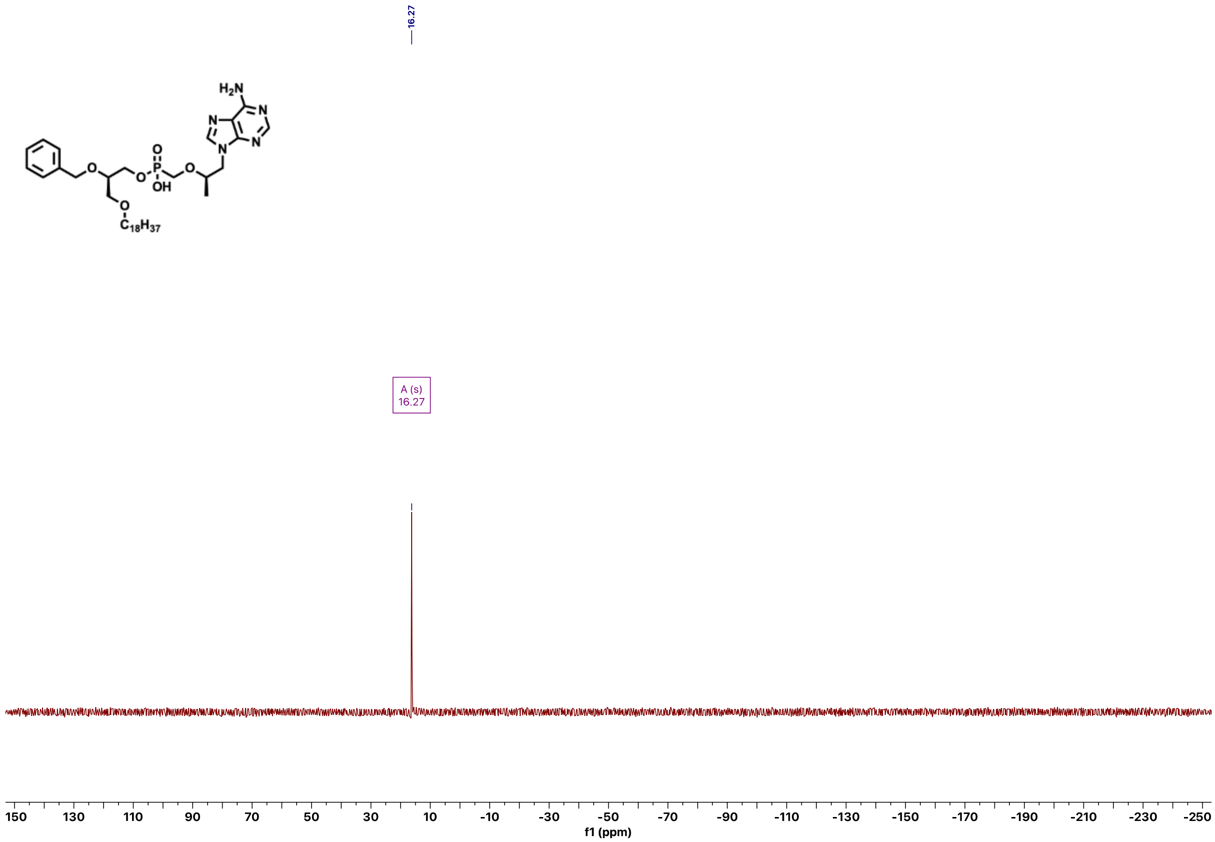

Figure S16. 8d ^{31}P NMR (162 MHz, CD_3OD).

*Ammonium[(1R)-2-(6-Aminopurin-9-yl)-1-methyl-ethoxy]-methyl-[3-(16,16,16-trifluorohexadecoxy)propoxy]phosphinate (**15a**)* as previously described.[1]

*Ammonium [(1R)-2-(6-aminopurin-9-yl)-1-methyl-ethoxy]methyl-[3-(18,18,18-trifluorooctadecoxy)propoxy]phosphinate (**15b**).* ^{1}H NMR (400 MHz, CD_3OD), ^{13}C NMR (101 MHz, CD_3OD), ^{19}F NMR (376 MHz, CD_3OD), ^{31}P NMR (162 MHz, CD_3OD).

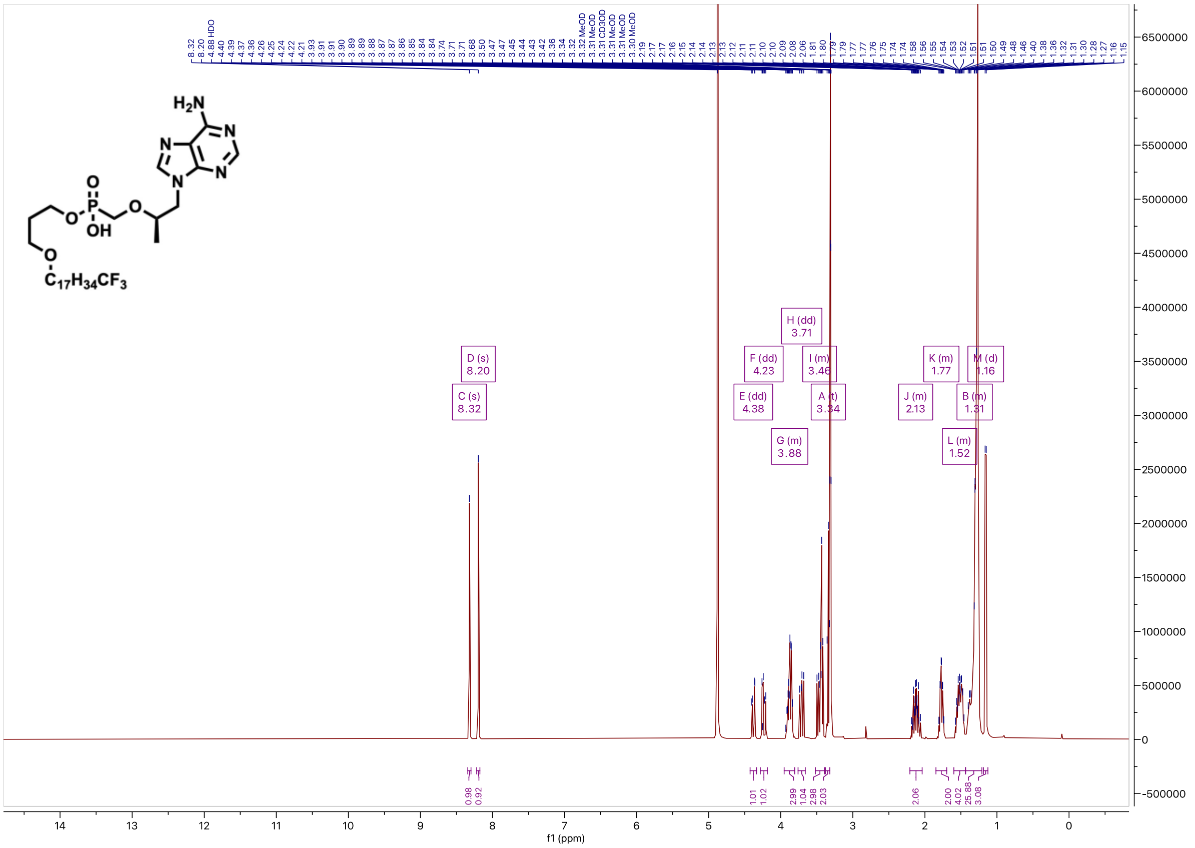

Figure S17. 15b ^{1}H NMR (400 MHz, CD_3OD).

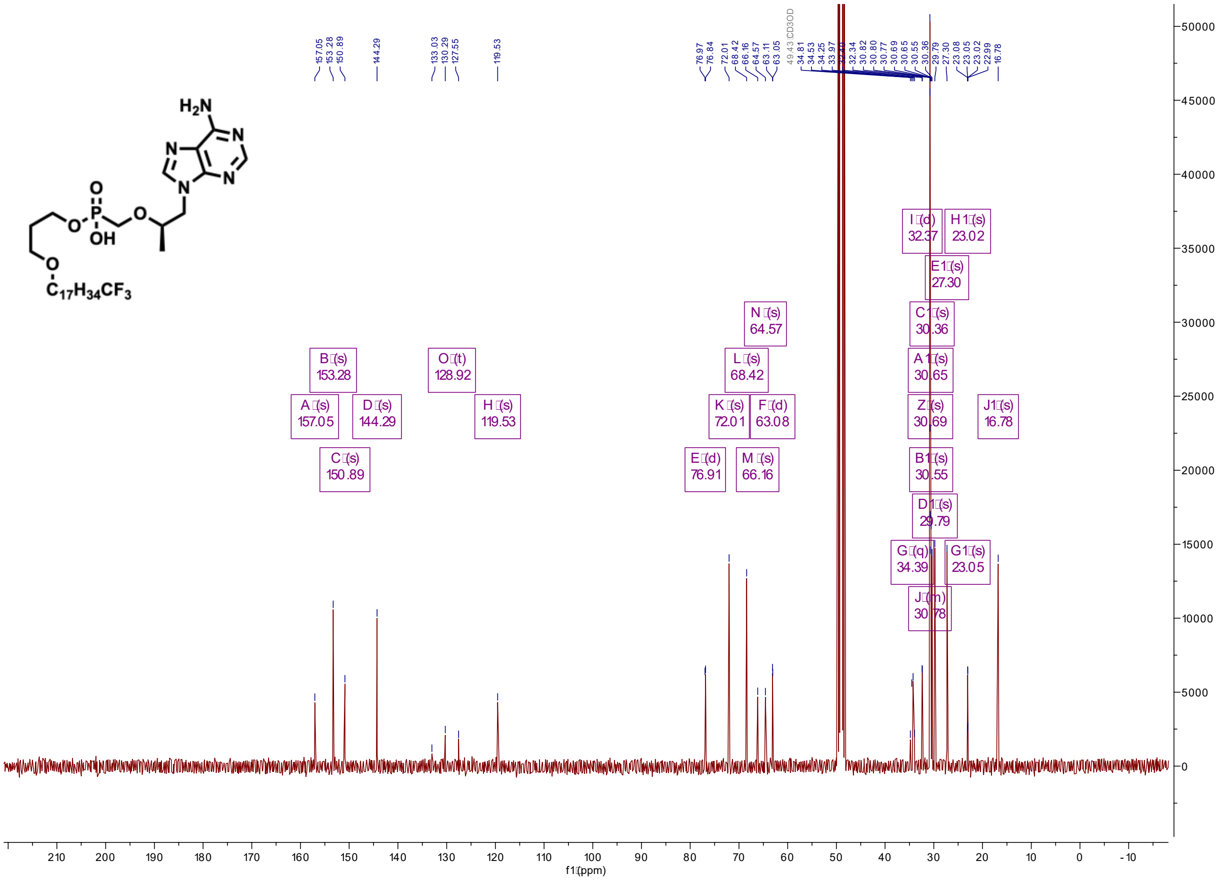

Figure S18. 15b ^{13}C NMR (101 MHz, CD_3OD).

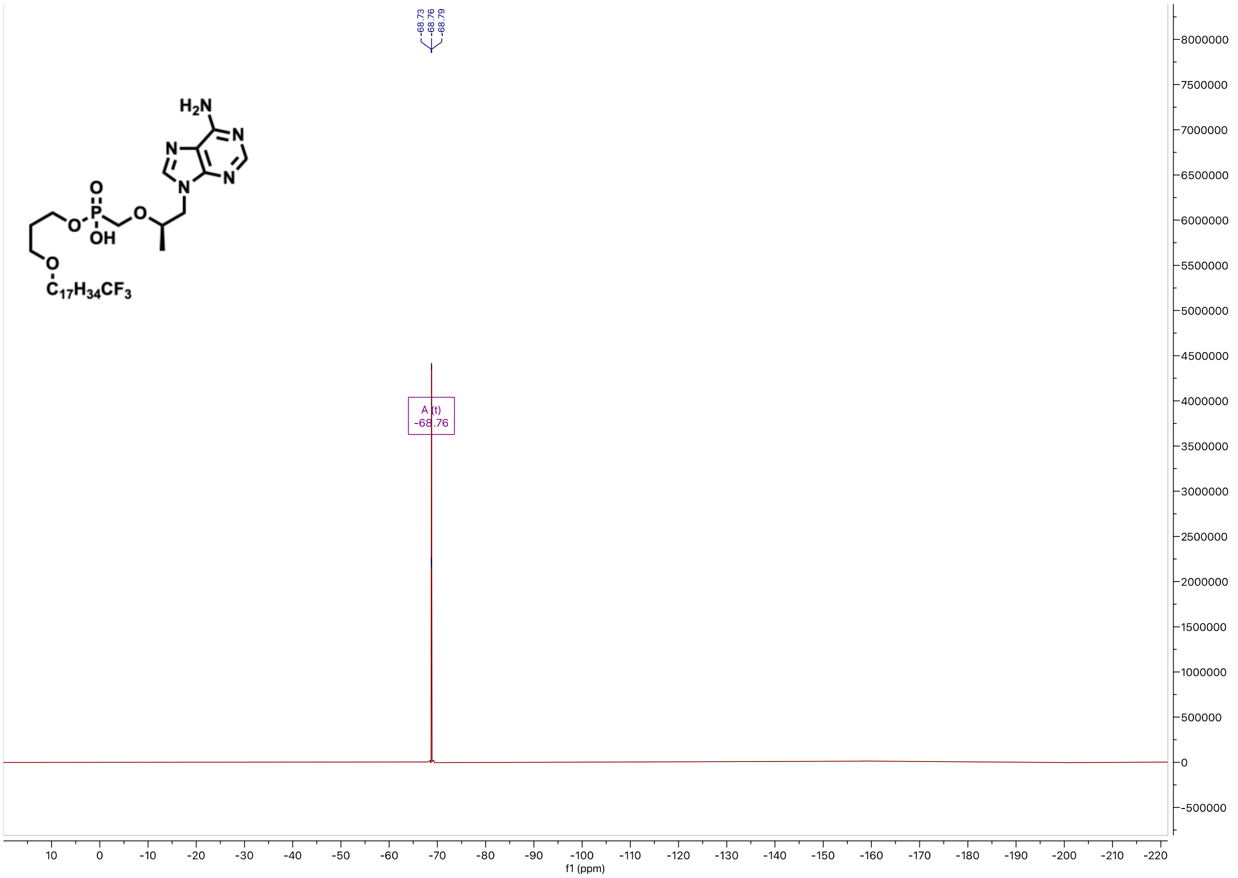

Figure S19. 15b ^{19}F NMR (376 MHz, CD_3OD).

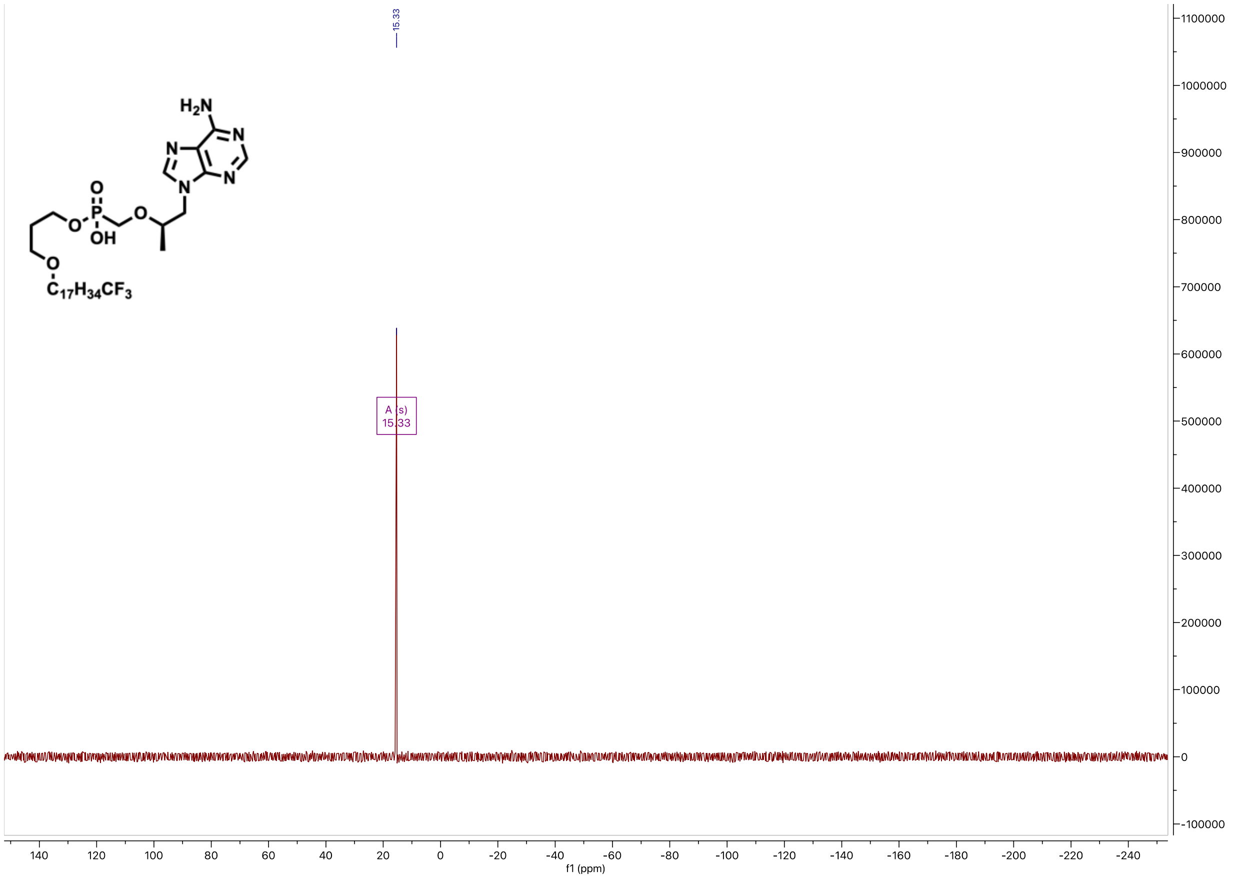

Figure S20. 15b ^{31}P NMR (162 MHz, CD_3OD).

*[(1R)-2-(6-aminopurin-9-yl)-1-methyl-ethoxy]methyl-[(2S)-2-benzyloxy-3-(16,16,16-trifluorohexadecoxy)propoxy]phosphinate (**21a**).* ^{1}H NMR (400 MHz, CD_3OD), ^{13}C NMR (151 MHz, CD_3OD), ^{19}F NMR (376 MHz, CD_3OD), ^{31}P NMR (162 MHz, CD_3OD).

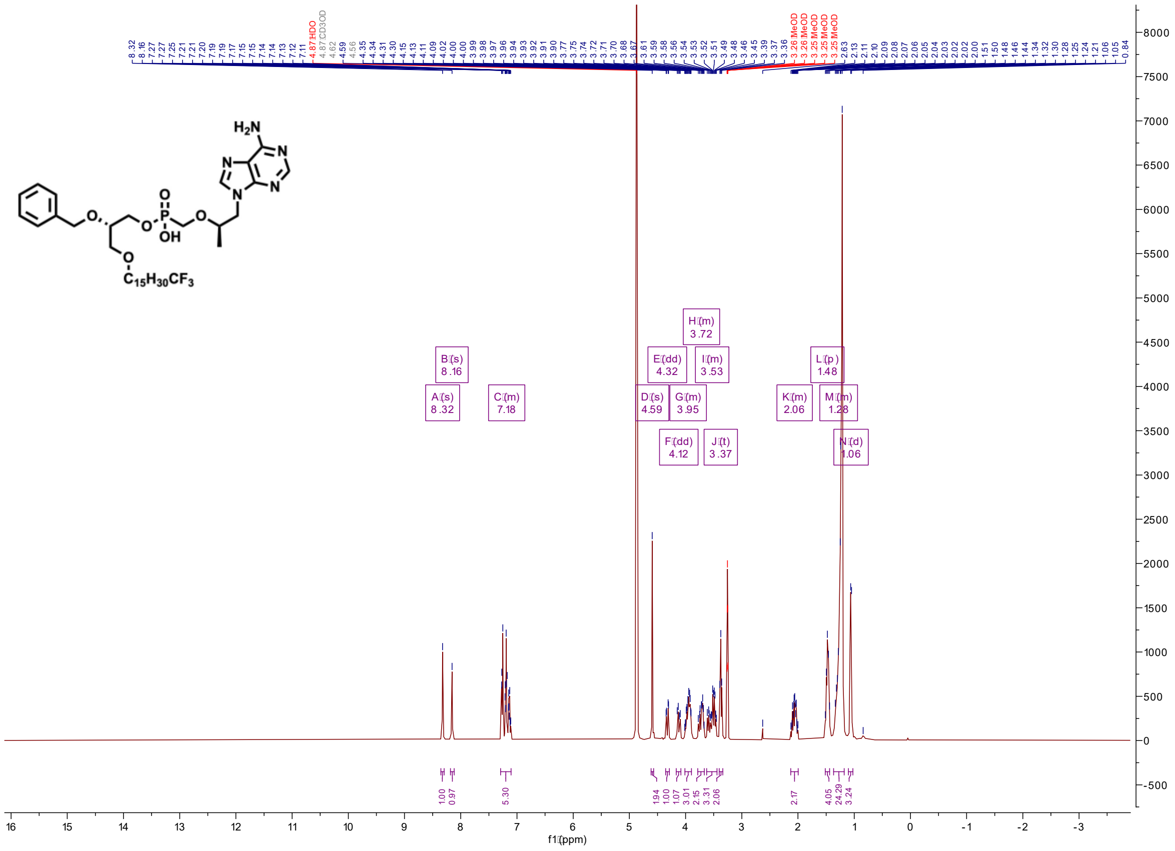

Figure S21. 21a ^{1}H NMR (400 MHz, CD_3OD).

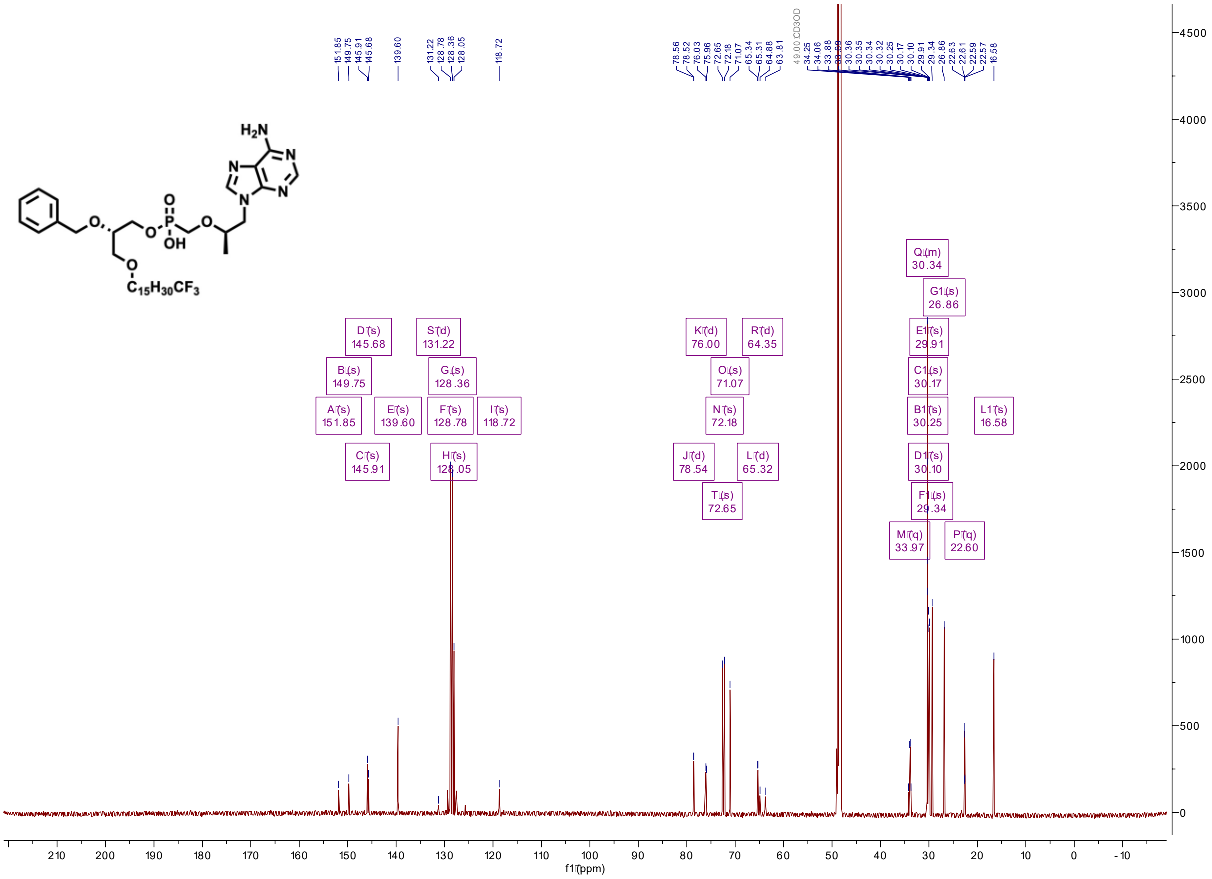

Figure S22. 21a ^{13}C NMR (151 MHz, CD_3OD).

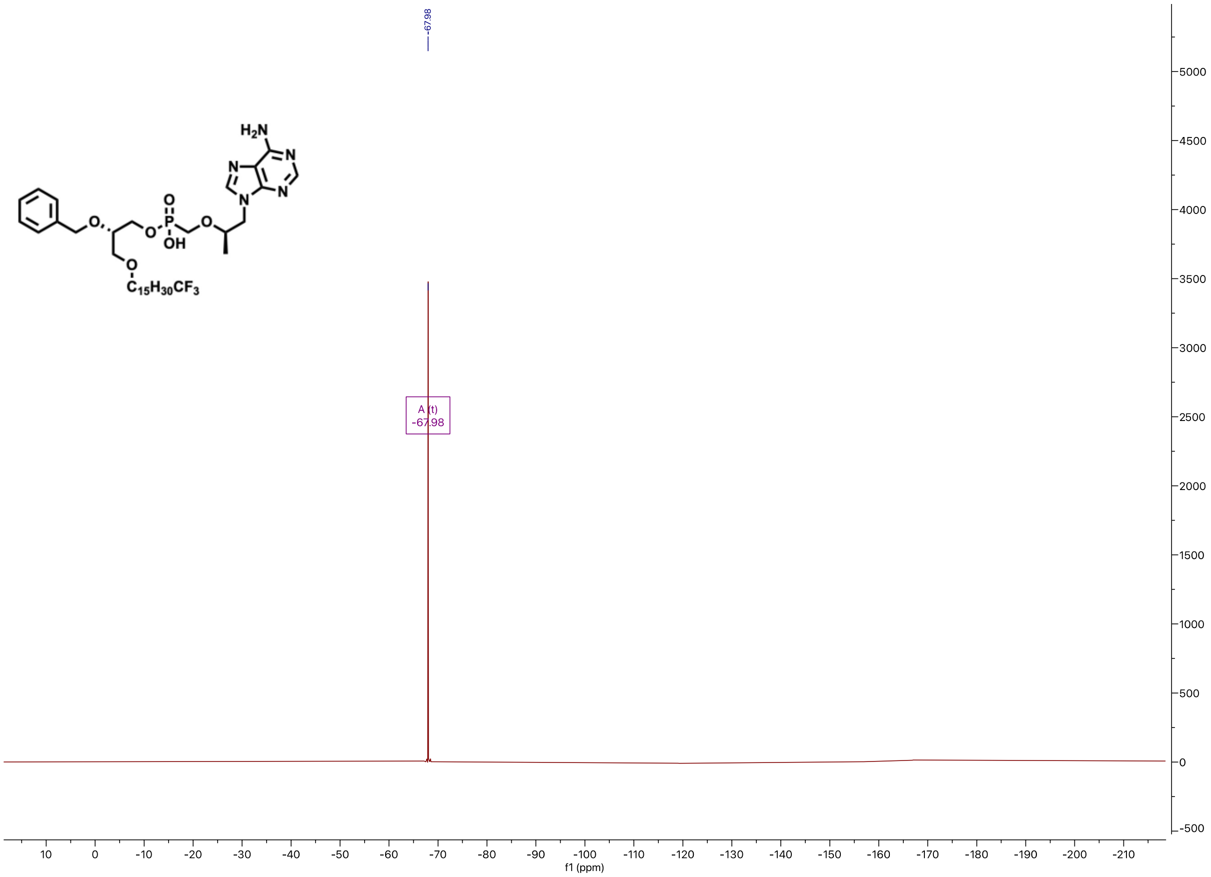

Figure S23. 21a ^{19}F NMR (376 MHz, CD_3OD).

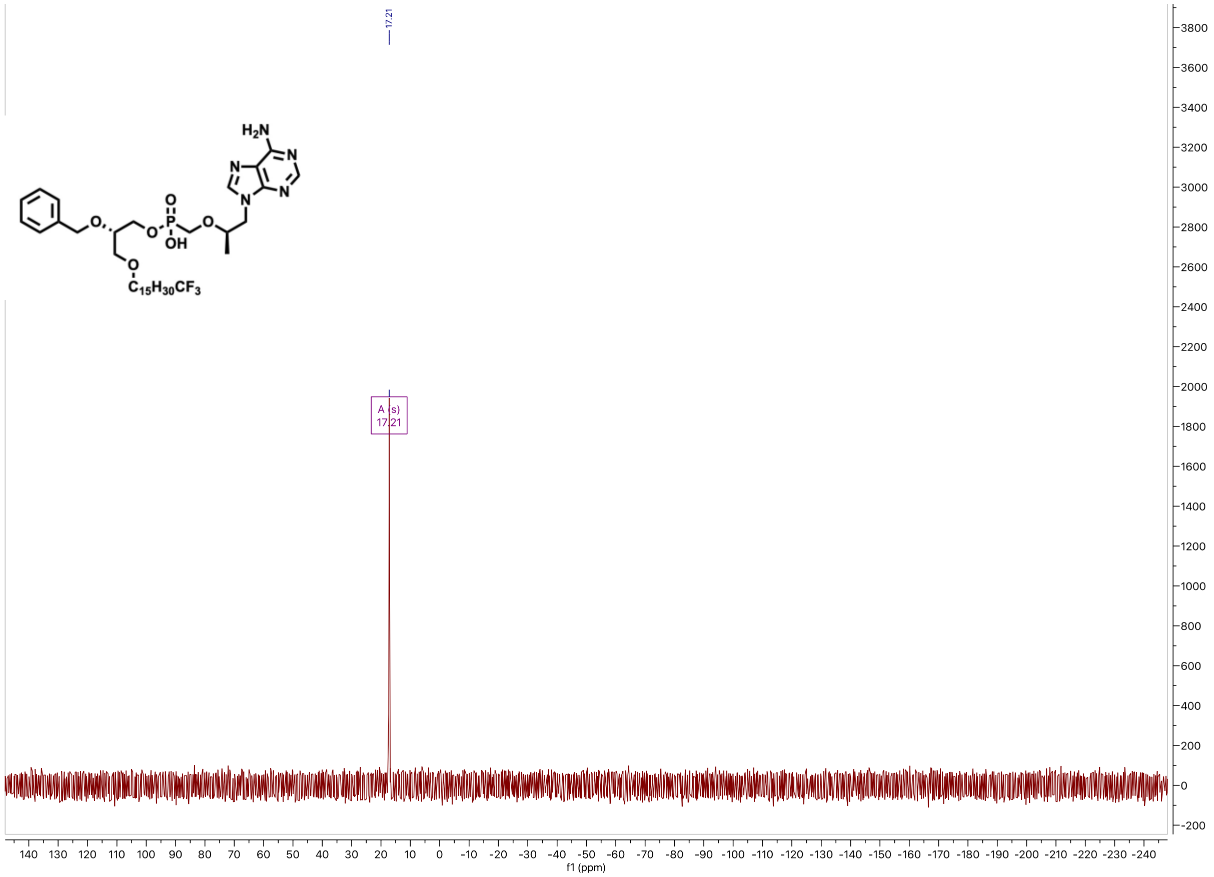

Figure S24. 21a ^{31}P NMR (162 MHz, CD_3OD).

[(1R)-2-(6-aminopurin-9-yl)-1-methyl-ethoxy]methyl-[(2S)-2-benzyloxy-3-(18,18,18-trifluorooctadecoxy)propoxy]phosphinate (***21b***). ^{1}H NMR (400 MHz, CD_3OD), ^{13}C NMR (151 MHz, CD_3OD), ^{19}F NMR (376 MHz, CD_3OD), ^{31}P NMR (162 MHz, CD_3OD).

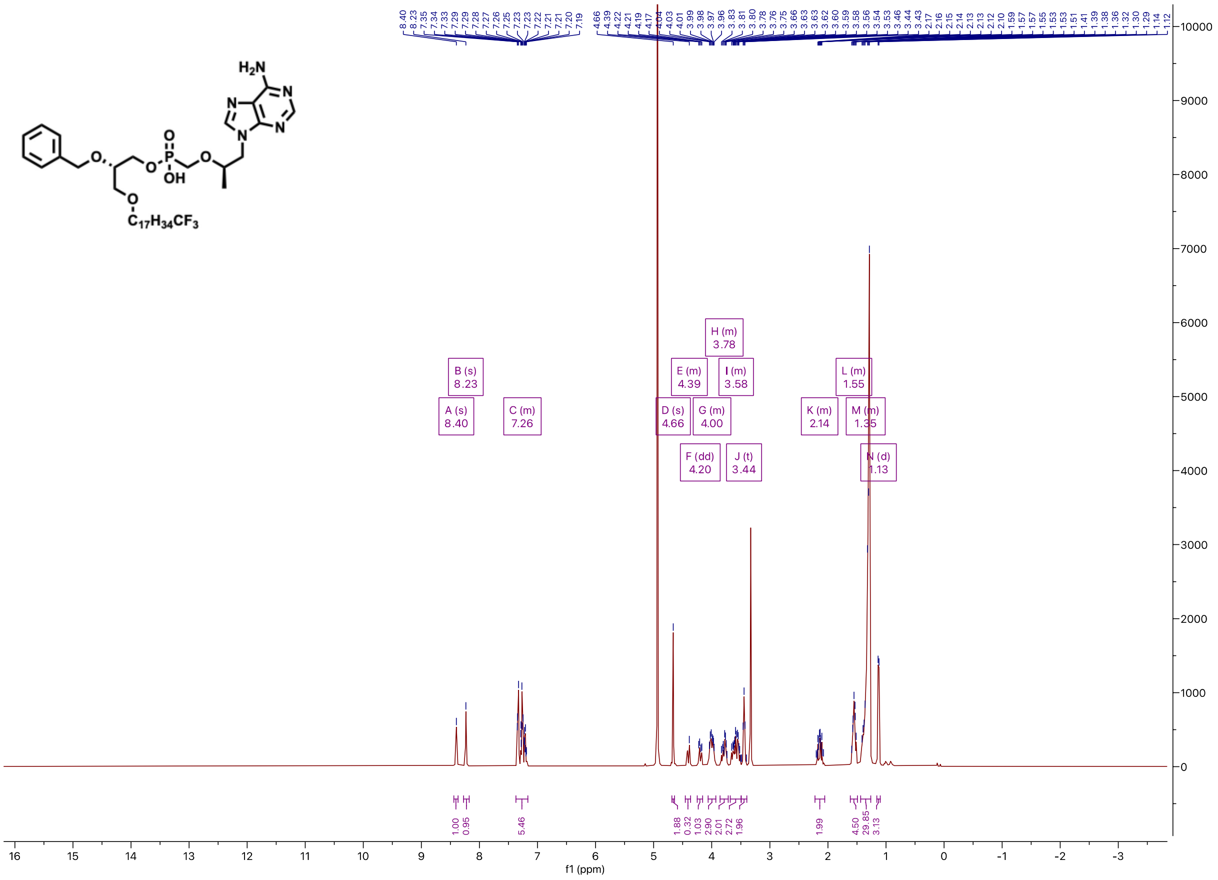

Figure S25. 21b ^{1}H NMR (400 MHz, CD_3OD).

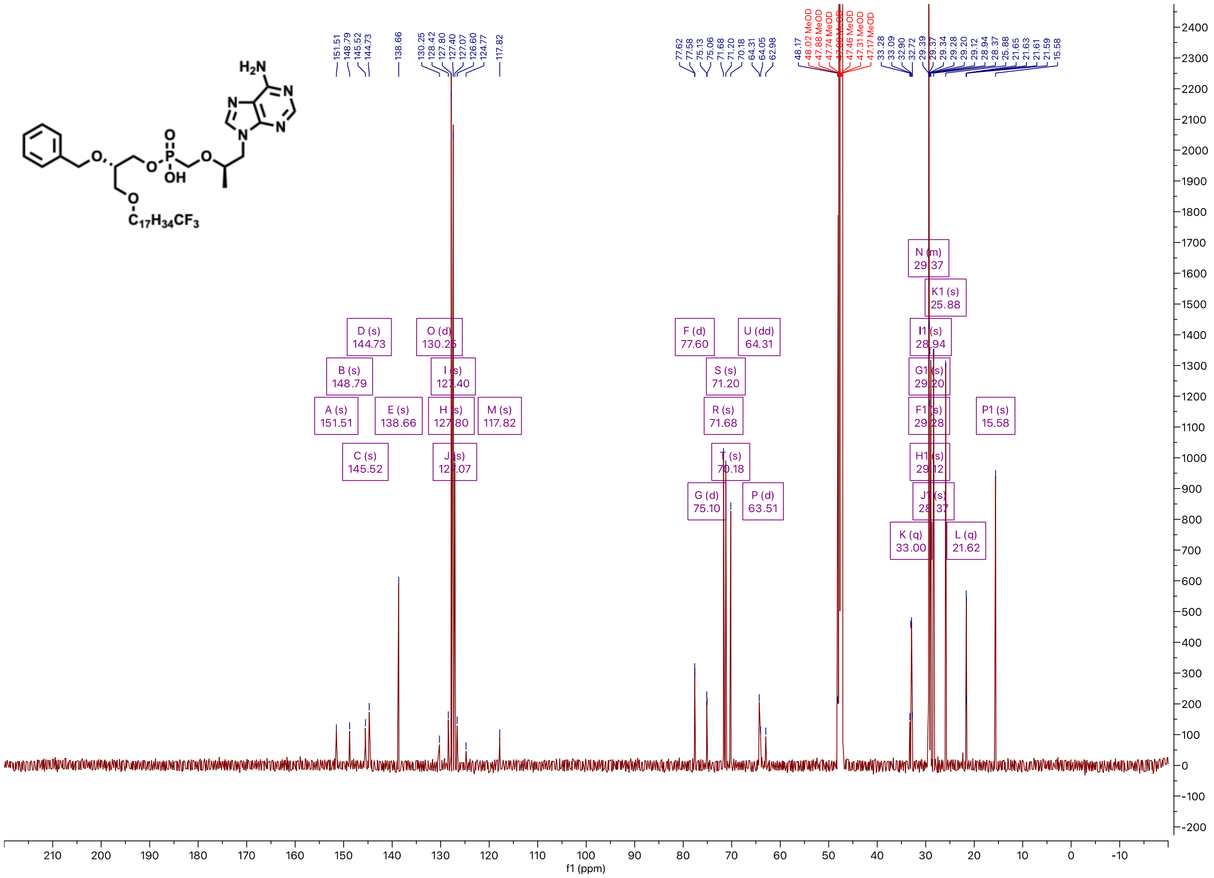

Figure S26. 21b ^{13}C NMR (151 MHz, CD_3OD).

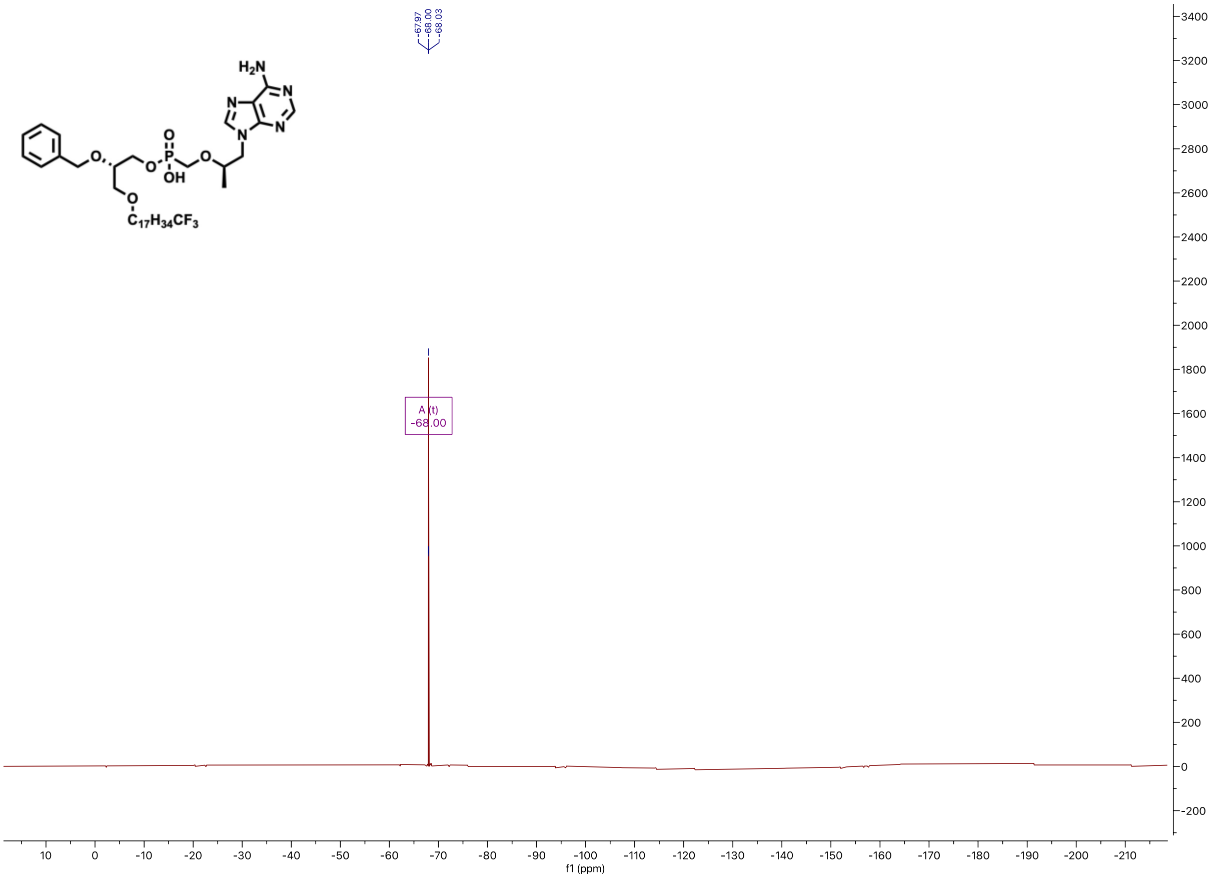

Figure S27. 21b ^{19}F NMR (376 MHz, CD_3OD).

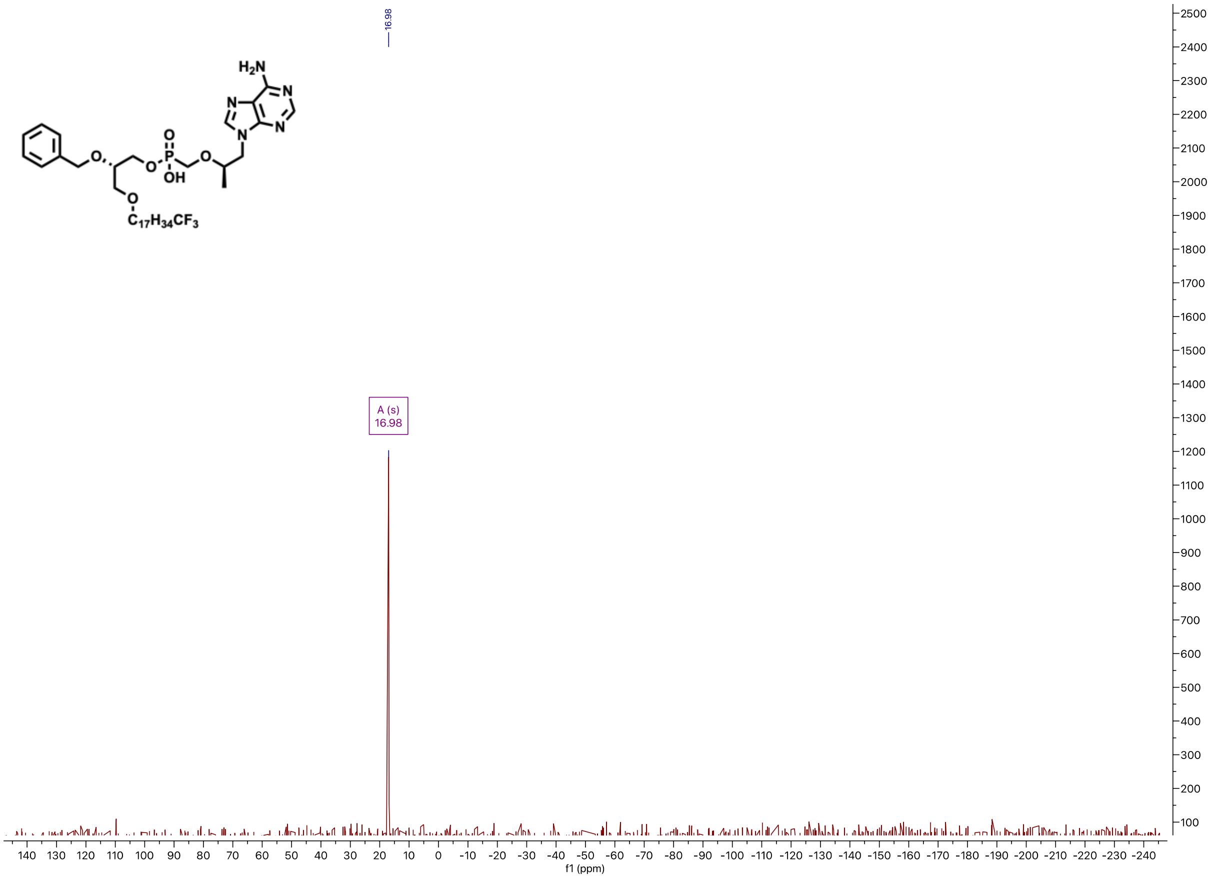

Figure S28. 21b ^{31}P NMR (162 MHz, CD_3OD).

FINAL COMPOUND LC-MS TRACES

Method: 75-95% ACN in H_2O over 6 min (254 nm).

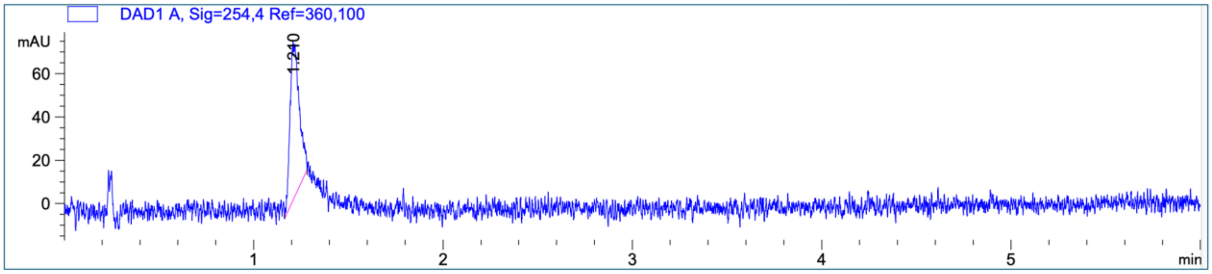

Peak #	RetTime [min]	Type	Width [min]	Area [mAU*s]	Height [mAU]	Area %
1	1.210	BB	0.0440	243.09232	71.88111	100.0000
Totals :				243.09232	71.88111	

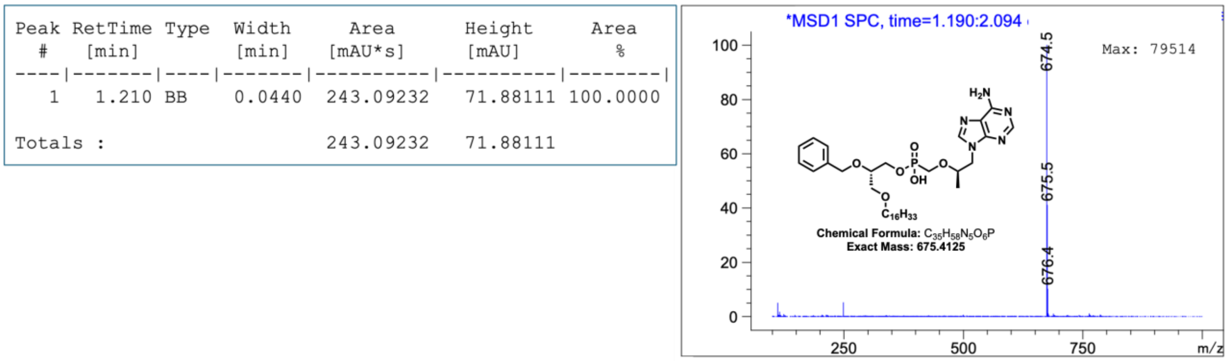

Figure S29. LC-MS trace of compound **8a**.

Method: 60-95% MeOH in H_2O over 6 min (254 nm).

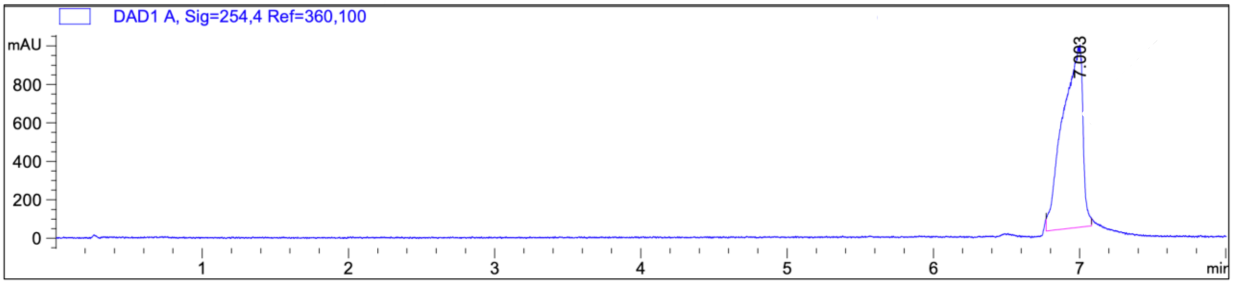

Signal 1: DAD1 A, Sig=254,4 Ref=360,100

Peak #	RetTime [min]	Type	Width [min]	Area [mAU*s]	Height [mAU]	Area %
1	7.003	MM	0.1578	8990.44824	949.56818	100.0000
Totals :				8990.44824	949.56818	

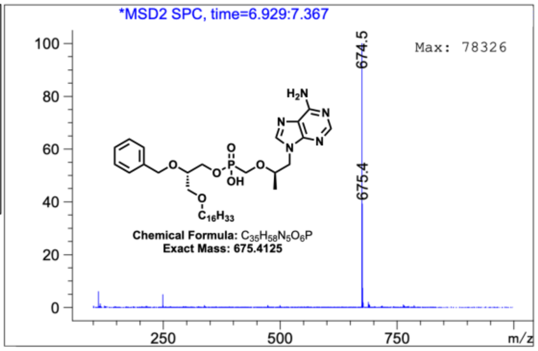

Figure S30. LC-MS trace of compound **8a**.

Method: 50-95% ACN in H_2O w. 0.1% formic acid over 6 min (254 nm).

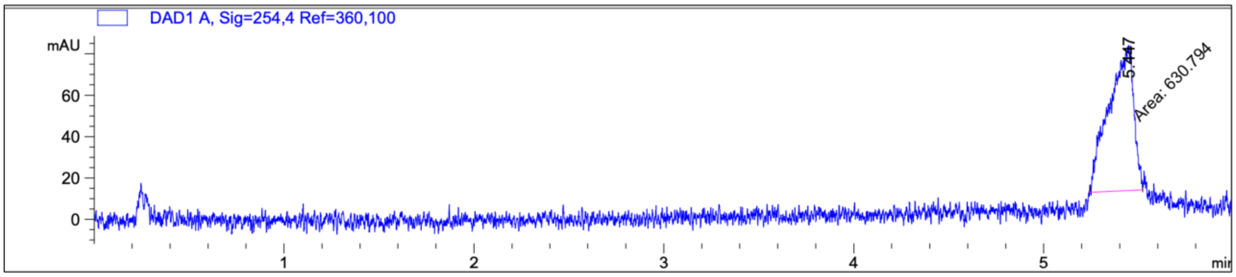

Peak #	RetTime [min]	Type	Width [min]	Area [mAU*s]	Height [mAU]	Area %
1	5.447	MM	0.1498	630.79413	70.16754	100.0000
Totals :				630.79413	70.16754	

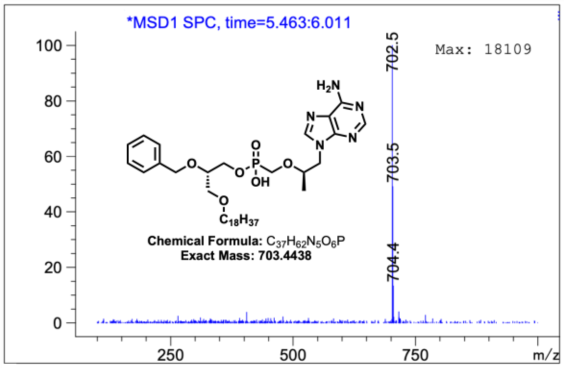

Figure S31. LC-MS trace of compound **8b**.

Method: 75-95% ACN in H_2O w. 0.1% formic acid over 6 min (254 nm).

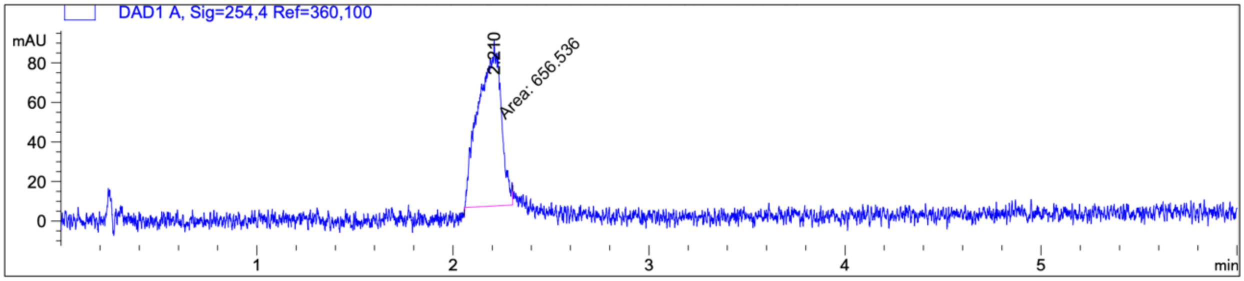

Peak #	RetTime [min]	Type	Width [min]	Area [mAU*s]	Height [mAU]	Area %
1	2.210	MM	0.1308	656.53613	83.66019	100.0000
Totals :				656.53613	83.66019	

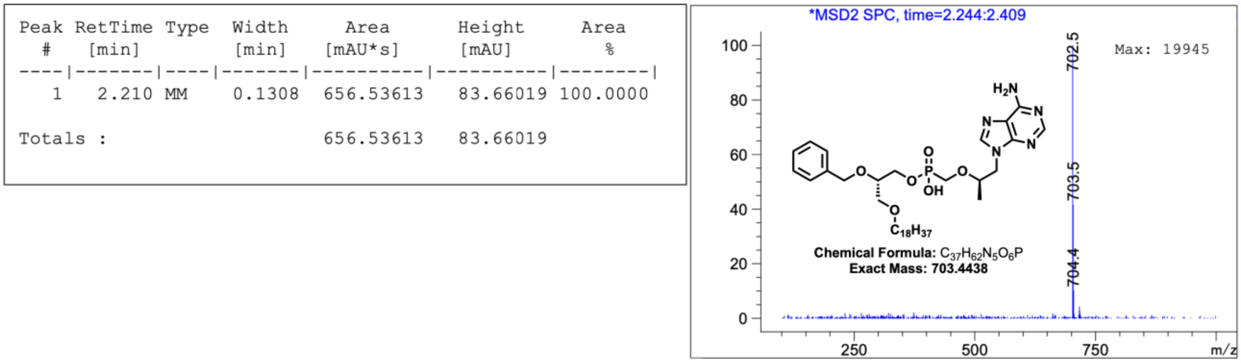

Figure S32. LC-MS trace of compound **8b**.

Method: 75-95% ACN in H_2O w. 0.1% formic acid over 6 min (254 nm).

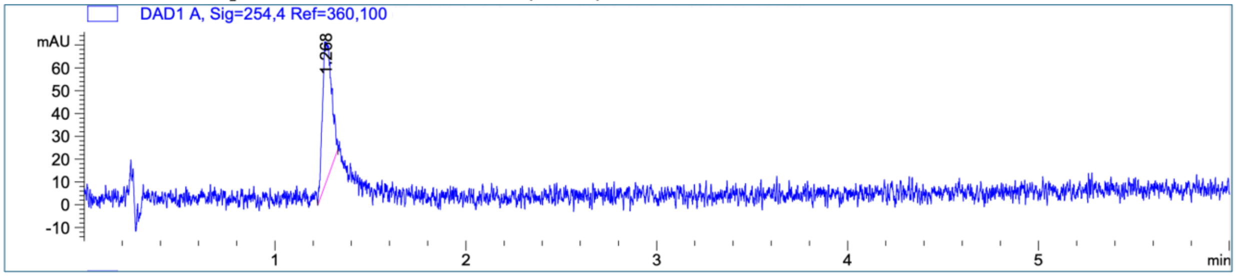

Peak #	RetTime [min]	Type	Width [min]	Area [mAU*s]	Height [mAU]	Area %
1	1.268	BB	0.0421	201.43010	60.95642	100.0000
Totals :				201.43010	60.95642	

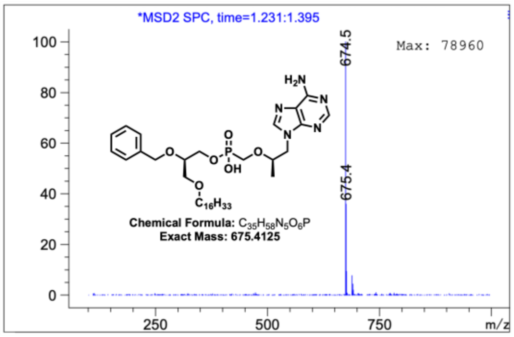

Figure S33. LC-MS trace of compound **8c.**

Method: 50-95% ACN in H_2O w. 0.1% formic acid over 6 min (254 nm).

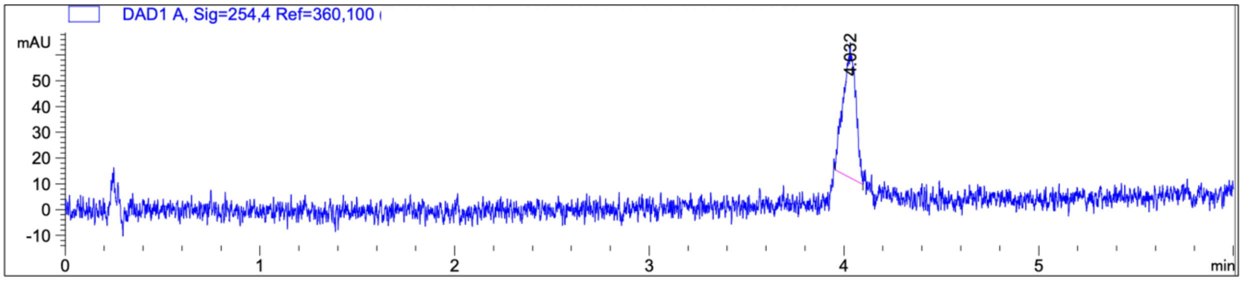

Peak #	RetTime [min]	Type	Width [min]	Area [mAU*s]	Height [mAU]	Area %
1	4.032	BB	0.0531	228.17345	51.07177	100.0000
Totals :				228.17345	51.07177	

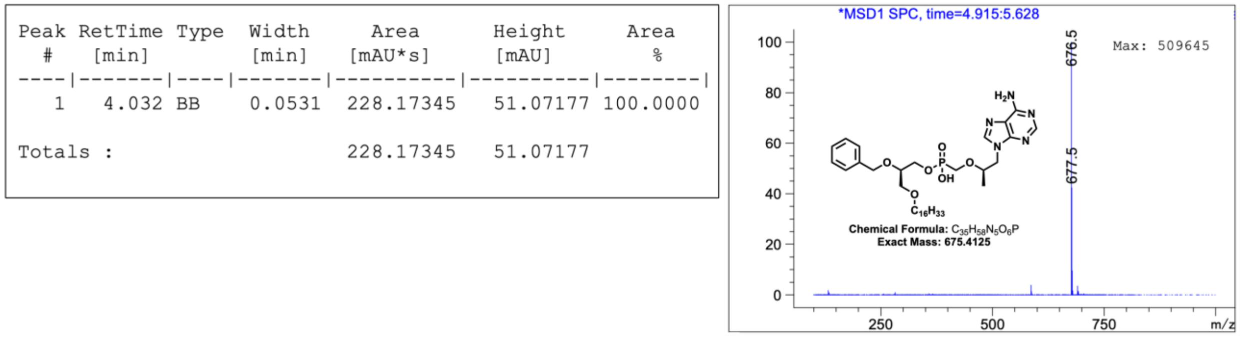

Figure S34. LC-MS trace of compound **8c**.

Method: 55-95% ACN in H_2O w. 0.1% formic acid over 6 min (254 nm).

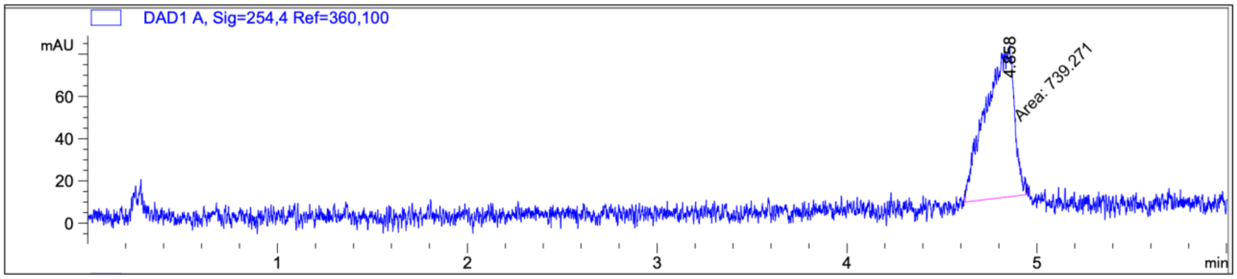

Peak #	RetTime [min]	Type	Width [min]	Area [mAU*s]	Height [mAU]	Area %
1	4.858	MM	0.1720	739.27136	71.61868	100.0000
Totals :				739.27136	71.61868	

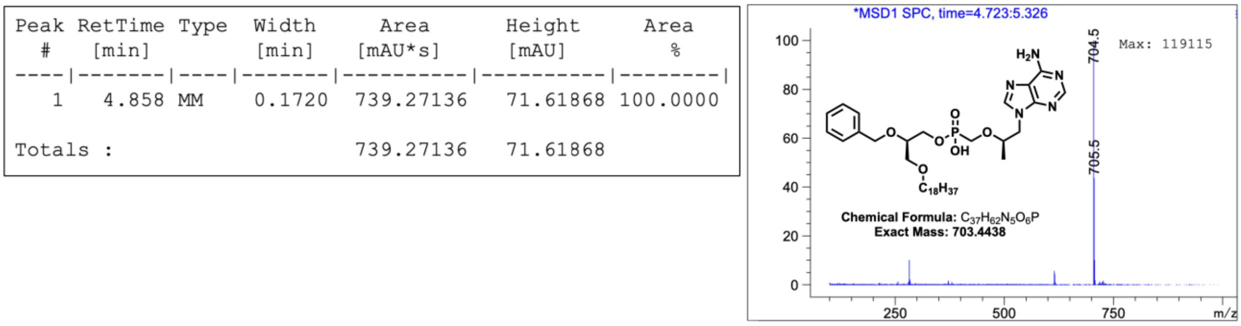

Figure S35. LC-MS trace of compound **8d**.

Method: 85-95% ACN in H_2O w. 0.1% formic acid over 6 min (254 nm).

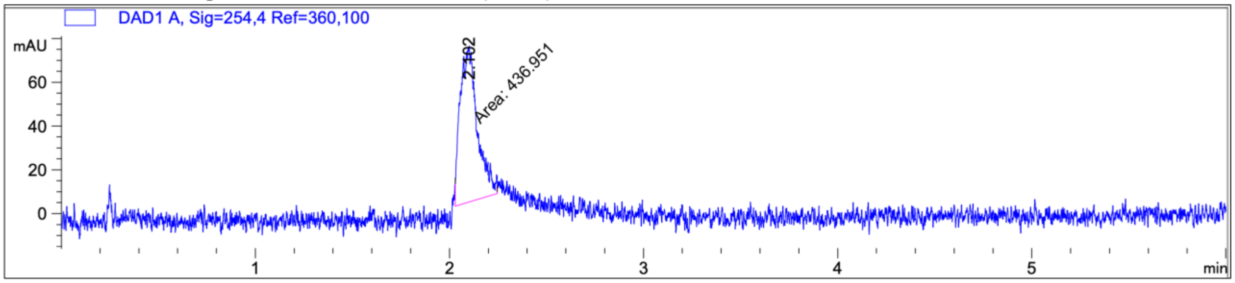

Peak #	RetTime [min]	Type	Width [min]	Area [mAU*s]	Height [mAU]	Area %
1	2.102	MM	0.1023	436.95117	71.19336	100.0000
Totals :				436.95117	71.19336	

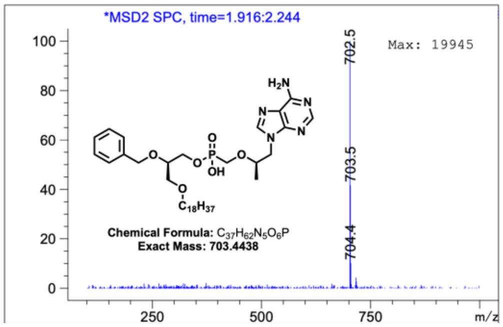

Figure S36. LC-MS trace of compound **8d**.

Method: 45-95% ACN in H_2O w. 0.1% formic acid over 6 min (254 nm).

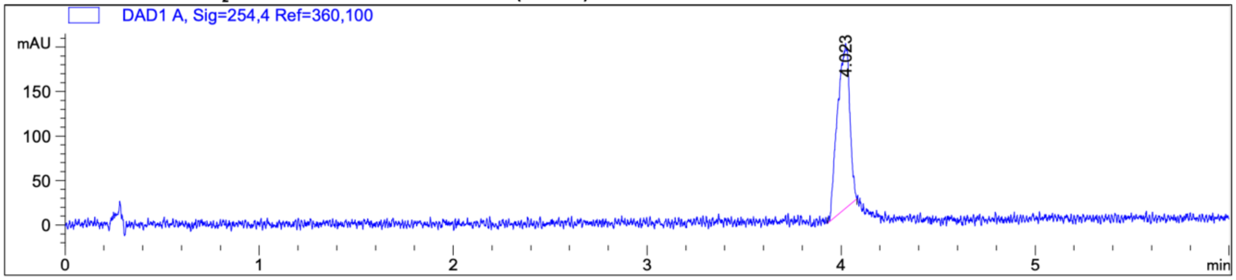

Peak #	RetTime [min]	Type	Width [min]	Area [mAU*s]	Height [mAU]	Area %
1	4.023	BB	0.0530	817.08344	183.24585	100.0000
Totals :				817.08344	183.24585	

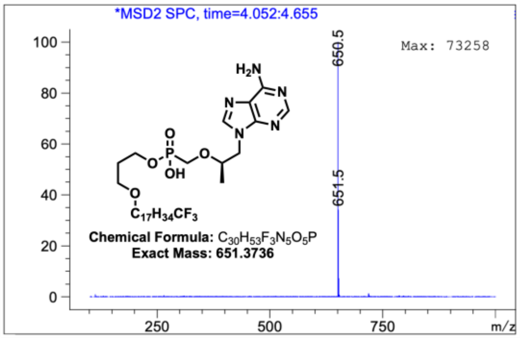

Figure S37. LC-MS trace of compound **15b**.

Method: 65-95% ACN in H_2O w. 0.1% formic acid over 6 min (254 nm).

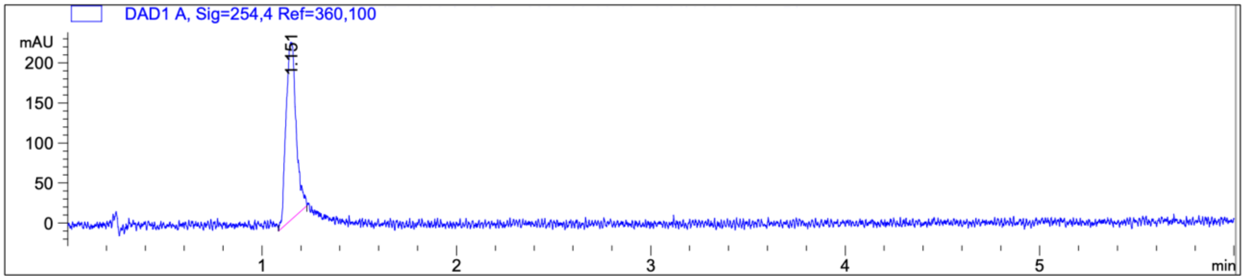

Peak #	RetTime [min]	Type	Width [min]	Area [mAU*s]	Height [mAU]	Area %
1	1.151	BB	0.0510	803.19495	220.65352	100.0000
Totals :				803.19495	220.65352	

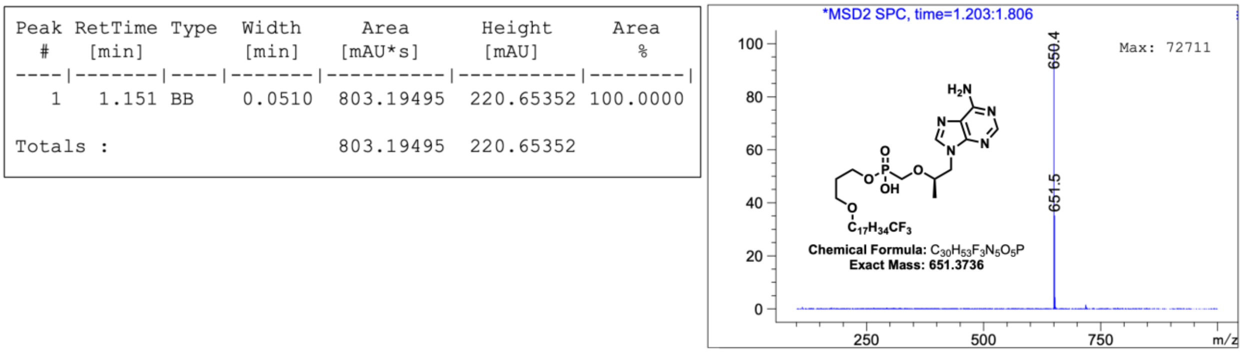

Figure S38. LC-MS trace of compound **15b**.

Method: 60-95% MeOH in H_2O over 6 min (254 nm).

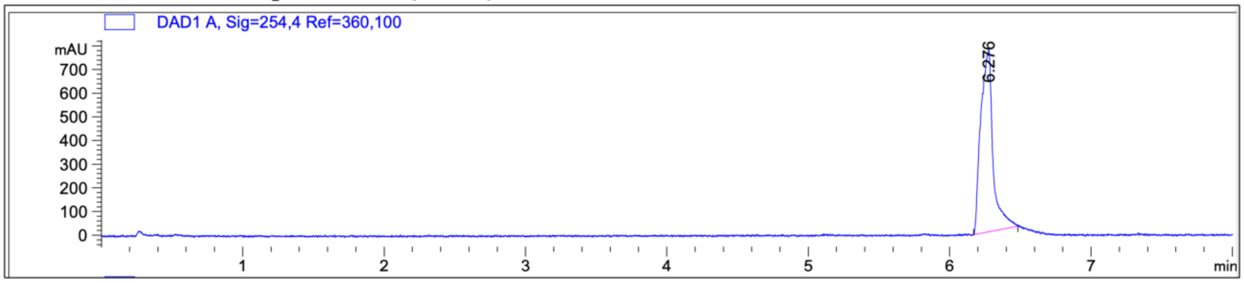

Peak #	RetTime [min]	Type	Width [min]	Area [mAU*s]	Height [mAU]	Area %
1	6.276	BB	0.0725	4618.72754	770.15326	100.0000
Totals :				4618.72754	770.15326	

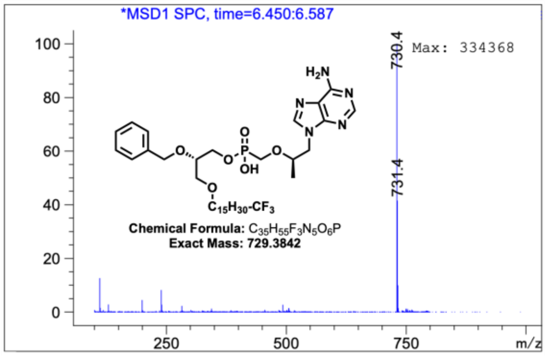

Figure S39. LC-MS trace of compound **21a**.

Method: 75-95% MeOH in H_2O over 6 min (254 nm).

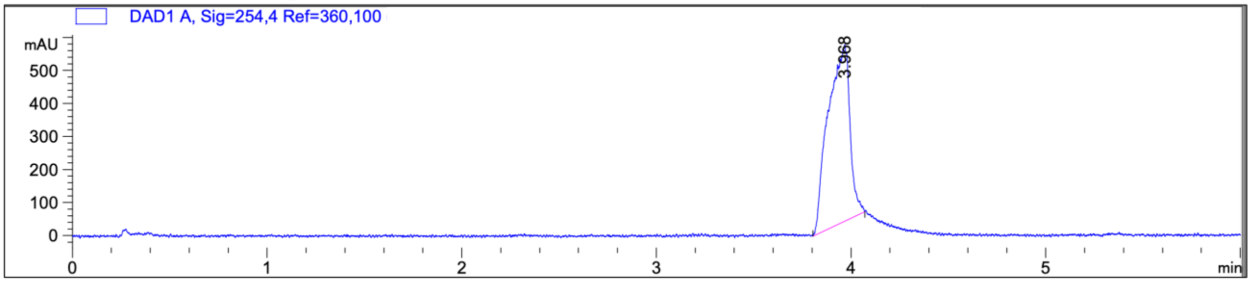

Peak #	RetTime [min]	Type	Width [min]	Area [mAU*s]	Height [mAU]	Area %
1	3.968	BB	0.0933	4170.49268	533.11230	100.0000
Totals :				4170.49268	533.11230	

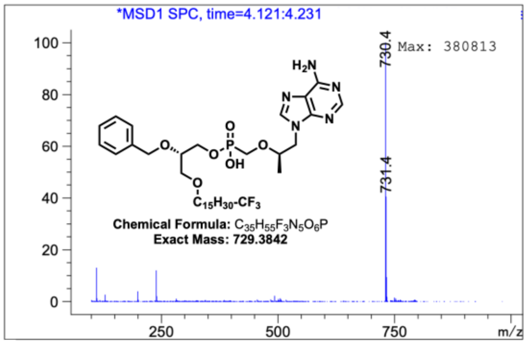

Figure S40. LC-MS trace of compound **21a**.

Method: 50-95% ACN in H_2O w. 0.1% formic acid over 6 min (254 nm).

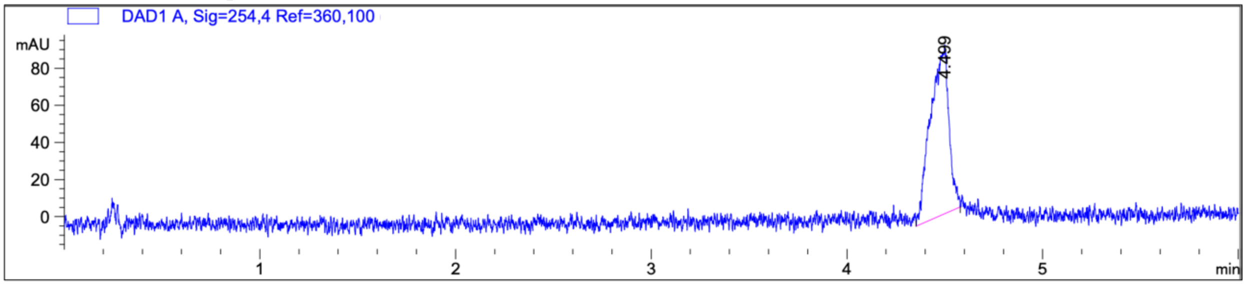

Peak #	RetTime [min]	Type	Width [min]	Area [mAU*s]	Height [mAU]	Area %
1	4.499	BB	0.0772	595.55975	91.21854	100.0000
Totals :				595.55975	91.21854	

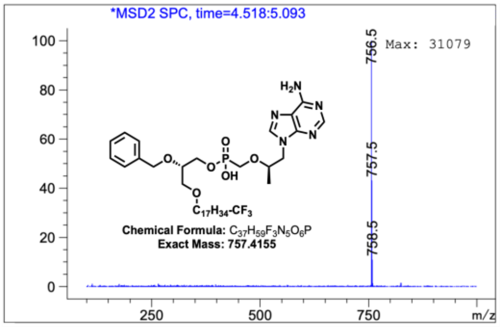

Figure S41. LC-MS trace of compound **21b**.

Method: 75-95% ACN in H_2O w. 0.1% formic acid over 6 min (254 nm).

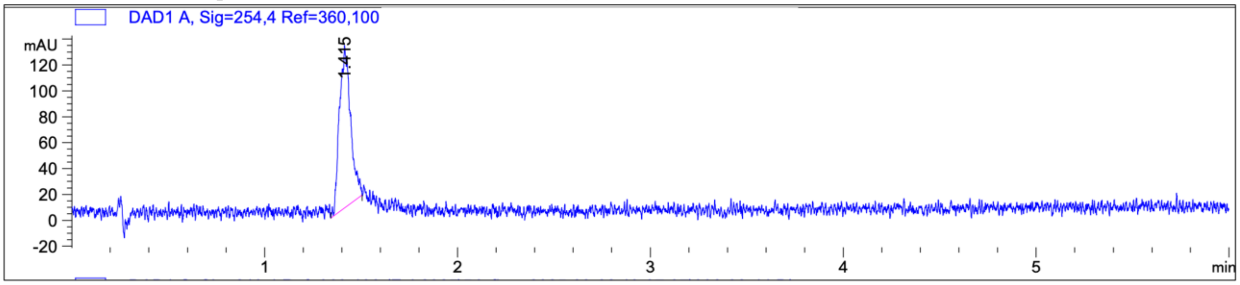

Peak #	RetTime [min]	Type	Width [min]	Area [mAU*s]	Height [mAU]	Area %
1	1.415	BB	0.0480	499.46149	125.43281	100.0000
Totals :				499.46149	125.43281	

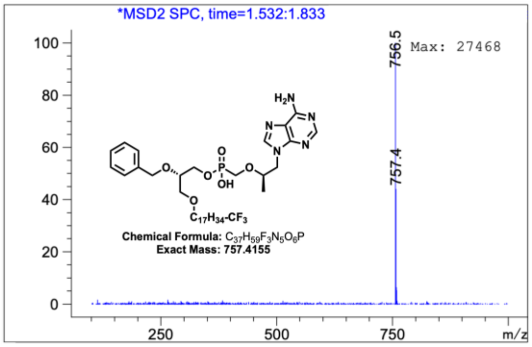

Figure S42. LC-MS trace of compound **21b**.

(1) Pribut, N.; D'Erasmo, M.; Dasari, M.; Giesler, K. E.; Iskandar, S.; Sharma, S. K.; Bartsch, P. W.; Raghuram, A.; Bushnev, A.; Hwang, S. S.; et al. ω-Functionalized Lipid Prodrugs of HIV NtRTI Tenofovir with Enhanced Pharmacokinetic Properties. *J Med Chem* **2021**, *64* (17), 12917-12937. DOI: 10.1021/acs.jmedchem.1c01083 From NLM.

(2) Derdeyn, C. A.; Decker, J. M.; Sfakianos, J. N.; Wu, X.; O'Brien, W. A.; Ratner, L.; Kappes, J. C.; Shaw, G. M.; Hunter, E. Sensitivity of human immunodeficiency virus type 1 to the fusion inhibitor T-20 is modulated by coreceptor specificity defined by the V3 loop of gp120. *J Virol* **2000**, *74* (18), 8358-8367. DOI: 10.1128/jvi.74.18.8358-8367.2000 From NLM.

(3) Wei, X.; Decker, J. M.; Liu, H.; Zhang, Z.; Arani, R. B.; Kilby, J. M.; Saag, M. S.; Wu, X.; Shaw, G. M.; Kappes, J. C. Emergence of resistant human immunodeficiency virus type 1 in patients receiving fusion inhibitor (T-20) monotherapy. *Antimicrob Agents Chemother* **2002**, *46* (6), 1896-1905. DOI: 10.1128/aac.46.6.1896-1905.2002 From NLM.

(4) Iordanskiy, S.; Bukrinsky, M. Reverse transcription complex: the key player of the early phase of HIV replication. *Future Virol* **2007**, *2* (1), 49-64. DOI: 10.2217/17460794.2.1.49 From NLM.

(5) Kratz, F. Albumin as a drug carrier: Design of prodrugs, drug conjugates and nanoparticles. *Journal of Controlled Release* **2008**, *132* (3), 171-183. DOI: https://doi.org/10.1016/j.jconrel.2008.05.010.

(6) Bowman, C. M.; Benet, L. Z. An examination of protein binding and protein-facilitated uptake relating to in vitro-in vivo extrapolation. *Eur J Pharm Sci* **2018**, *123*, 502-514. DOI: 10.1016/j.ejps.2018.08.008 From NLM.
